# Rare‐Earth Multivariate Metal–Organic Frameworks: Cationic Radius Biased Compositional Control and Property Tuning

**DOI:** 10.1002/anie.5287849

**Published:** 2026-09-26

**Authors:** Adnan Ishaq, Joseph O. Ogar, Asif Raza, Musa M. Mahmud, Jacob L. Brownlee, Danielle E. Schier, Stephen P. Argent, Lucy Clark, Soumya Mukherjee, Neil R. Champness

**Affiliations:** ^1^ School of Chemistry University of Birmingham Edgbaston Birmingham UK; ^2^ School of Chemistry University of Nottingham Nottingham UK; ^3^ Bernal Institute and Research Ireland Centre for Pharmaceuticals (SSPC), Department of Chemical Sciences University of Limerick Limerick Ireland

**Keywords:** metal–organic frameworks, multivariate frameworks, porous materials, structural diversity

## Abstract

The synthesis of a multivariate metal–organic framework (MTV‐MOF) family, **[Ln(DNNDI)_1.5_]_n_** (**UoB‐116**), is reported (DNNDI: *N*,*N'*‐bis(dinicotinate)‐1,4,5,8‐naphthalenetetracarboxydiimide). **UoB‐116(Ln)** is formed by 15 different metals (Ln = Y, La, Ce, Pr, Nd, Sm, Eu, Gd, Tb, Dy, Ho, Er, Tm, Yb, and Lu), all of which were shown to be isoreticular. The uniformity of the framework structure facilitated the synthesis of MTV‐MOFs with 2, 4, 12, 15, or 16 (including In(III)) different metals. Single crystal structures of each unimetallic MOFs and MTV‐MOFs with 12 or 16 metals were determined, confirming the isoreticular nature of this family of MOFs. Energy Dispersive X‐ray (EDX) analysis confirmed distribution of each metal throughout crystals of the MTV‐MOFs and compositional analysis indicated increased concentration of lanthanides with smaller cationic radius. A protocol was developed to control relative composition in bimetallic systems, **UoB‐116(La_1‐x_Lu_x_)** and **UoB‐116(Dy_1‐x_La_x_)** (*x* = 0–1). In the case of **UoB‐116(Dy_1‐x_La_x_)** it is shown that the porosity, magnetism and UV–visible properties can be tuned through metal cation composition.

## Graphical abstract

A family of multivariate metal–organic frameworks, **UoB‐116(Ln)**, can be prepared using up to 16 different metals, revealing the ability to tune properties through compositional control.

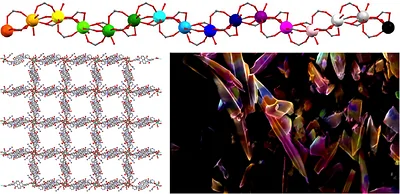

## 1 Introduction

Metal–organic frameworks (MOFs) containing *f*‐block metals are of great interest to researchers due to their ability to combine structural tunability, framework porosity, and the unique electronic and magnetic properties of the lanthanide (Ln) metals inaccessible to their d‐block counterparts [1]. Although it is indeed possible to synthesize MOFs based on *s*, *p*, *d*, and *f* block metals, *f*‐block metals perform well in a large range of applications. A particular advantage of incorporating lanthanide metals into MOFs stems from the increased flexibility of a lanthanide center's coordination geometry and the potential for higher coordination numbers, in comparison to *d*‐block metals. The flexibility of lanthanides can lead to far greater structural diversity in Ln‐MOFs as evidenced by our previous studies [2, 3, 4, 5] and examples such as those reported by Oliver et al. who produced four distinctly different frameworks from the same linker combined with different lanthanide metals [6]. Ln‐MOFs are also investigated for a range of applications, such as heterogeneous catalysis, sensing, bio‐imaging, as single‐molecule magnets and gas storage/separation among many others [7, 8, 9, 10, 11].

Mixed‐metal MOFs are a subset of multivariate (MTV) MOFs which have increased in popularity in recent times due to their ability to add an additional dimension of complexity to an already structurally versatile family of materials with relative ease. Chemists are now able to vary the relative distribution of two or more metals over the same node which allows for the fine tuning of properties such as band gaps, open coordination site chemistry, or magnetic properties, among others, along a gradient [12, 13, 14]. The potential for the emergence of synergistic effects is also possible through mixing multiple metals into structures with well‐defined metal clusters to enable new and interesting chemistry. For example, increasing the nickel composition of MIL‐100 led to far greater catalytic performance for the Prins condensation of β‐pinene to pinol [15]. Futhermore, it has been shown that the addition of further metals can lower the free energy of the framework and improve the chemical and thermal stability of the overall material [16, 17].

Despite the aforementioned rise in popularity for mixed‐metal MTV‐MOF research, most systems in the literature tend to combine five or fewer metals, with the vast majority of research on *d*‐block metal MOFs. Little research has been done toward developing a fundamental understanding of the factors that affect the incorporation of one metal in comparison to another in Ln‐based MTV‐MOFs. One study revealed preferential incorporation of rare earth metals with smaller ionic radii in a Ln‐MOF from bimetallic mixtures, including those with adjacent lanthanides (e.g. Yb/Tm) [18]. However, this study focused on ionic radii effect for bimetallic MOFs, rather than for MTV‐MOFs that incorporate larger numbers of different metal cations. In an elegant recent study, Sava Gallis and coworkers have produced a MOF incorporating 15 different rare earth metals, distributed in Ln_9_ clusters, and bridged by tetracarboxylate ligands [19]. In the current study, we have added to this by producing a MOF with every available rare earth metal, plus indium. This highlights the flexibility of the MOF to accommodate a metal from the *p*‐block, with indium to be incorporated into an otherwise *f*‐block framework. Additionally, to our knowledge, there are no reported MOFs containing metals from the *d*, *p*, and *f* block as constituents within the same framework. A thorough understanding of the energetic preference for the inclusion of one metal cation versus another metal cation in a framework material will prove key to producing MOFs of a given relative metal composition for future applications.

NDI‐based (NDI: 1,4,5,8‐naphthalenetetracarboxydiimide) ligands are a suitable building block in MOF design due to their highly planar π‐electron‐deficient core which combines a low energy LUMO suitable for redox activity, with the inherent desire for NDI connectors to arrange in a π‐stacking manner, or to π‐stack with guests within the cavity of the MOF [20, 21]. A large library of NDI based ligands can be synthesized with ease by the reaction of naphthalene dianhydride with an amine of choice to produce a ligand with targeted properties, structure, or connectivity [22]. Additionally, synergies between the energy levels of the lanthanide cations and NDIs are well researched in the literature, originally observed in lanthanide‐NDI complexes but later harnessed in MOFs [23, 24]. For example, Bai et al. synthesized a series of Ln NDI MOFs which exhibit strong guest‐responsive *f*‐*f* emission, which is credited to a π* to Ln energy transfer [25].

Herein we synthesize a series of lanthanide‐based NDI MOFs, **UoB‐116(Ln)**, based upon an original ligand (H_2_DNNDI) which presents a ditopic carboxylate‐based coordination mode, despite the presence of two pyridine donor atoms being present in terminal positions. We find that this framework is extremely receptive to accommodating the entire lanthanide series, as well as yttrium, a *d*‐block rare earth metal. In small quantities, indium, a *p*‐block metal can also be accommodated, representing the first MOF with *f*‐, *d*‐, and *p*‐block metals. We have successfully synthesized and characterized 15 single‐metal analogues of this MOF by single crystal X‐ray diffraction (SCXRD). In addition, we have been able to produce MOFs with up to 16 different metals, adding a *p*‐block metal to the Ln‐mixture, with uniform metal distribution confirmed by EDX. In this study, we find that even though equimolar amounts of each metal salt were used in the MTV‐MOF syntheses, the framework favors selection of those metals with smaller ionic radius during synthesis. We have been able to harness this effect to produce a La/Lu bimetallic analogue of specific composition. Additionally, to produce a bimetallic core‐shell type structure, a postsynthetic doping of additional metals has been shown to be possible. Ultimately our studies demonstrate a strategy for preparing Ln‐based MTV‐MOFs with the largest number of different metals, by the introduction of a *p*‐block metal, but importantly introduce a method for controlling the composition by understanding the influence of metal ionic radius on incorporation in the MTV‐MOFs. Further, we demonstrate that the metal cation composition of the MTV‐MOFs can be used to tune the properties of the resulting framework material.

## 2 Results and Discussion

### 2.1 Synthesis and Characterisation of Multivariate MOFs

The linker (*N*,*N'*‐bis(dinicotinic acid)‐1,4,5,8‐naphthalenetetracarboxydiimide: H_2_DNNDI) (Figure 1a) was synthesized by a simple one‐step condensation reaction between 1,4,5,8‐naphthalenetetracarboxylic dianhydride (NTCDA) and 5‐aminonicotinic acid, a prominent synthetic route to producing functional NDIs [22]. Perhaps unsurprisingly, we found that the purity of this ligand is crucial in producing crystals of quality for SCXRD analysis and so the crude was thoroughly purified (see experimental section).

**FIGURE 1:**
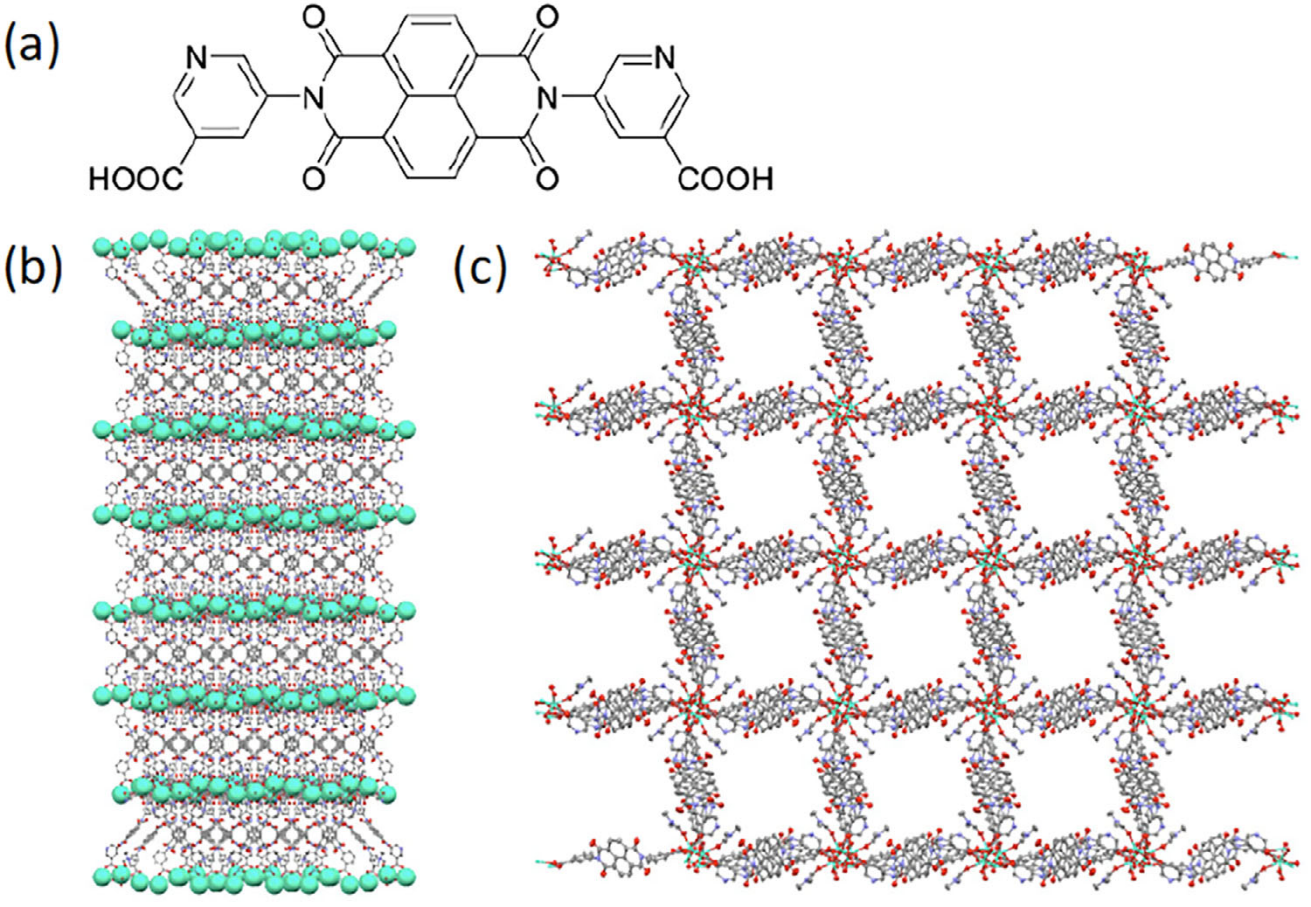
(a) Structure of the ligand H_2_DNNDI; (b, c) crystal structure of **UoB‐116(Tb)**, a representative example of the MOFs studied, noting that the entire series of frameworks exhibits the same structure. (b) View highlighting the chains of lanthanides cations; (c) the square‐shaped pores in the extended **UoB‐116** structure. (Tb, turquoise; O, red; C, black; N, light blue).

The series of **UoB‐116(Ln)** was prepared as yellow/orange needle shaped crystals from the reaction of Ln(NO_3_)_3_ (where Ln = Y, La, Ce, Pr, Nd, Sm, Eu, Gd, Tb, Dy, Ho, Er, Tm, Yb, and Lu) with DNNDI in 3.0 mL DMF. We found that the specific volume, 3.0 mL, yielded larger crystals than solutions with lower volumes, and higher concentrations. SCXRD data were collected for single crystals of these materials at 100 K using Cu Kα radiation. We were able to grow single crystals of MTV‐MOFs incorporating either 12 or 16 metals. In the case of these multimetallic crystals, synchrotron radiation was required to allow collection of data of sufficient quality for SCXRD analysis. The majority of the structures were found to crystallize in the space group *I*4_1_/*a*, however, there were a few outliers which crystallize in *C*2/*c*, and one in *I*2/*a*. This has been determined to be related to the ordering of DMF within the pores during the SCXRD data collection, and not a difference in the structure of the framework itself. The unit cell parameters do not change significantly based upon the metal in the framework (see Table S1) and powder X‐ray diffraction (PXRD) confirms the isoreticular nature of the **UoB‐116** family (Figure S5). In some of the MOFs, (M = Yb, Tm, and Dy), small splitting of the main PXRD reflections is observed, suggesting the presence of a MOF with slightly contracted dimensions.

The general structure of **UoB‐116(Ln)** comprises metal‐carboxylate chains extending along the crystallographic *c* direction (for *I*4_1_/*a* structures). Each lanthanide ion is bridged in a *syn*‐*syn* fashion to the next metal in the chain by four DNNDI^2−^ ligands, two in each direction along the chain, one DNNDI^2−^ ligand chelates the metal ion but also bridges to the next lanthanide through one of its carboxylate oxygens, and one DNNDI^2−^ ligand does the opposite—chelating the previous lanthanide but bridging to the original. Finally, one DMF molecule coordinates to each metal center through its carbonyl oxygen. The overall formula for the lanthanide chains is *catena*‐{[(DMF)tetrakis(μ_2_‐κO:κO′‐DNNDI^2^^−^)‐*bis*‐(μ_2_‐κ^2^O,O′:κO′‐DNNDI^2^^−^)lanthanide(III)]}_n_. The overall coordination gives rise to square‐shaped pores which can accommodate DMF solvent molecules (Figure 1). A related MOF has been reported combining *N*,*N*′‐bis(3‐benzoic acid)naphthalene diimide and Eu(III) which exhibits the same framework structure as the **UoB‐116(Ln)** structure [26].

Owing to the isostructural nature of the monometallic MOFs, a series of MTV‐MOFs of the same structure was synthesized, starting first with a bimetallic La/Lu system, **UoB‐116(La_1‐*x*_Lu_x_)** (*x* = 0–1). The mixed‐metal La/Lu MOF was characterized by EDX and was found to have a relative atomic composition of approximately 25%:75% respectively. A 4‐metal MTV‐MOF, **UoB‐116(Nd, Gd, Tb, Er)** was also synthesized. EDX maps were collected for both the 2‐ and 4‐ metal MTV‐MOFs (Figure 2) and, in both cases, showed an overall uniform distribution of each metal in individual crystals at the resolution of the measurement. This was the case both per‐crystal, and between different crystals. This contrasts with the findings of Yaghi et al. who postulate that in their 10‐metal system, each crystal has a different composition [27].

**FIGURE 2:**
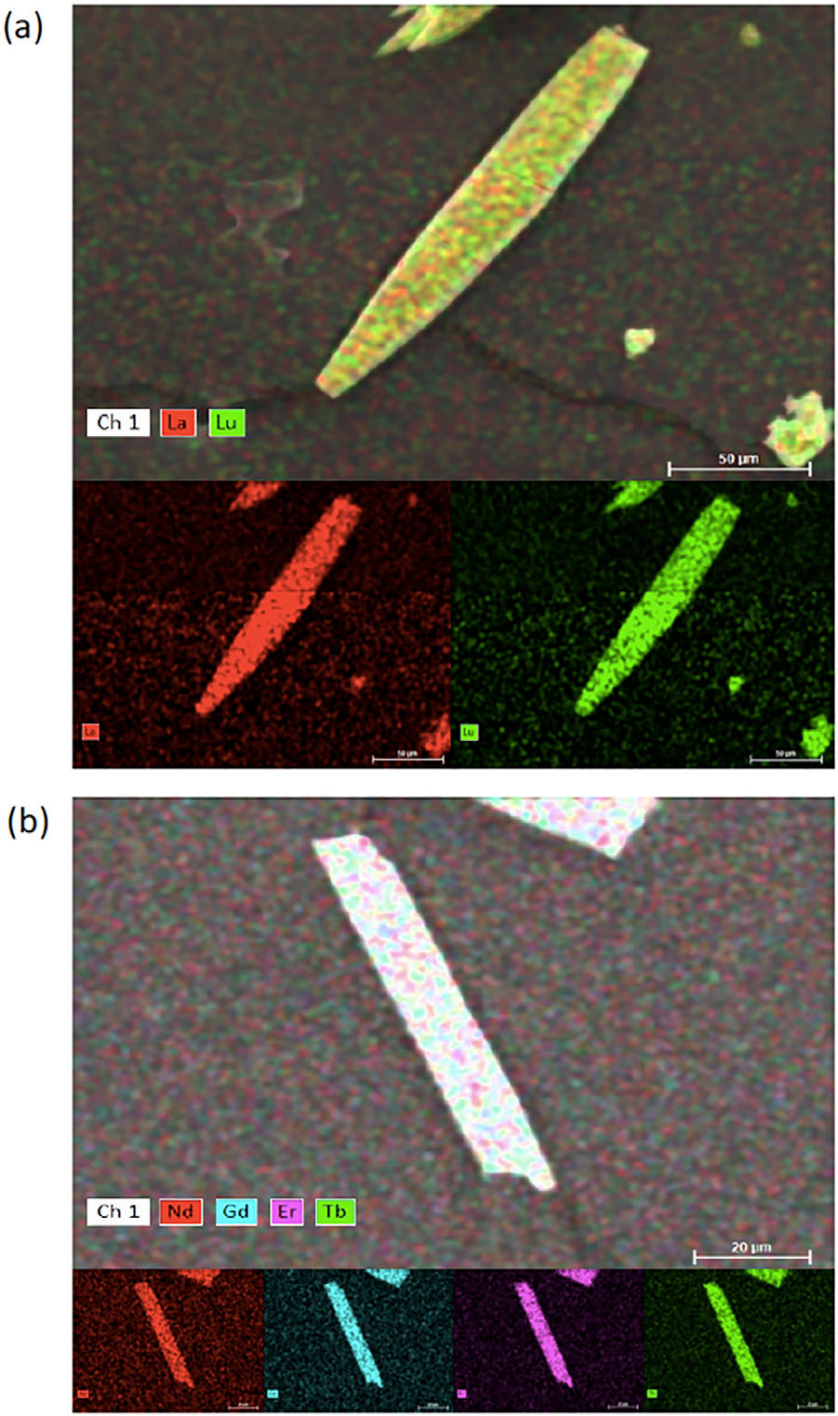
EDX maps showing the distribution of constituent metals in crystals of (a) **UoB‐116(La_0.25_Lu_0.75_)** and (b) **UoB‐116(Nd_0.11_Gd_0.27_Tb_0.37_Er_0.25_)**. Scale bar represents 20 µm.

Following the success of these syntheses, a 12‐metal MTV‐MOF, **UoB‐116(La, Ce, Pr, Nd, Eu, Gd, Tb, Dy, Ho, Er, Yb,** and **Lu)**, was synthesised and to confirm the accuracy of EDX measurements, inductively coupled plasma mass spectrometry (ICP‐MS) was used to evaluate the metal‐composition in the resulting material, which required digestion of the material. We found that the compositions determined by EDX and ICP‐MS were in agreement, further suggesting that the composition of the crystals are indeed homogenous and that the EDX measurements did not exhibit potential surface bias. The measurements from a ground sample were compared with whole crystals of the same material, the relative compositions determined by EDX were not significantly different, again establishing the homogeneity of the mixed metal systems. Although EDX confirms homogeneity at the micrometer level, we cannot exclude nanoscale compositional heterogeneity. An overlaid EDX map was acquired for the 12‐metal material on a SEM‐TF Apreo 2S HiVac instrument (Figure 3) which highlights the presence of every metal in the crystal. A SCXRD structure was collected for this 12‐metal material which indicated that no long‐range order of the occupancy of the Ln cation site.

**FIGURE 3:**
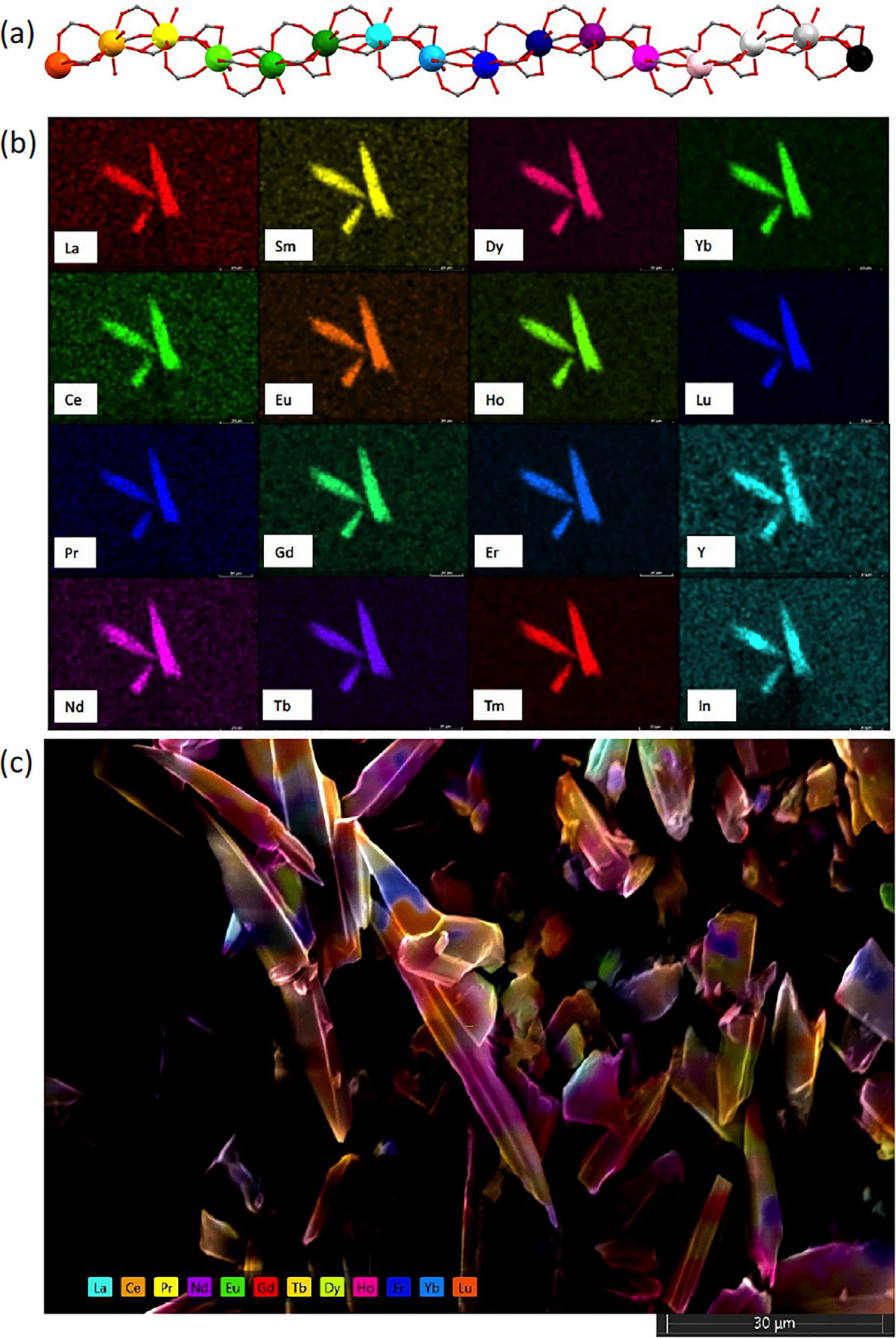
(a) Representation of one possible configuration of 16 different metals along the Ln‐carboxylate chain in **UoB‐116(Y, In, La, Ce, Pr, Nd, Sm, Eu, Gd, Tb, Dy, Ho, Er, Tm, Yb,** and **Lu)**, each colored sphere representing a different metal. We note that although this example shows one cation of each distinct metal this is not necessarily the case in any given fragment of the MOF, indeed each cationic site could be occupied by any of the metals in a random fashion. (b) EDX maps showing the distribution of constituent metals in crystals of **UoB‐116(Y, In, La, Ce, Pr, Nd, Sm, Eu, Gd, Tb, Dy, Ho, Er, Tm, Yb,** and **Lu)**, scale bar represents 20 µm. (c) Overlaid EDX maps showing the presence of all 12 metals in the crystals of **UoB‐116(La, Ce, Pr, Nd, Eu, Gd, Tb, Dy, Ho, Er, Yb,** and **Lu)**. The colors illustrate the most abundant metal at a given location, but analysis of the overall data confirm uniform distribution of metals throughout the sample.

An MTV‐analogue containing all 15 (purchasable) rare earth metals (lanthanides plus Y(III)), **UoB‐116(Y, La, Ce, Pr, Nd, Sm, Eu, Gd, Tb, Dy, Ho, Er, Tm, Yb,** and **Lu)**, was synthesized and characterized by EDX, confirming the presence of all 15 cations. This led us to explore whether a *p*‐block metal could also be included in the MTV‐MOF and In(III) was chosen as its ionic radius approaches the range of the lanthanide series (see Figure 5a). Thus, an MTV‐MOF with all 15 plus In(III) was synthesised, **UoB‐116(Y, In, La, Ce, Pr, Nd, Sm, Eu, Gd, Tb, Dy, Ho, Er, Tm, Yb,** and **Lu)**, which to our knowledge, contains the greatest number of metals in a single MOF, 16, with the previous record standing at 15 [19]. To our knowledge, this is the only MOF of its kind containing metals from the *d*, *p*, and *f*‐blocks. The composition was confirmed by EDX, and a SCXRD structure was collected for the 16‐metal analogue which confirmed the structure of the MOF was maintained despite the inclusion of 16 different cations within the same framework.

The current study is distinct from most previous reports of MTV‐MOFs using Ln(III) cations. To date, many Ln‐based MTV‐MOFs have focused on MOFs that incorporate discrete secondary building units (SBUs), in a number of cases with clusters that incorporate nine Ln(III) cations [2, 19, 28, 29, 30, 31, 32, 33, 34, 35]. In the current study, the Ln‐based SBU consists of a Ln‐carboxylate one‐dimensional chain, related to that observed in the previous study by Li et al. [36]. Chains provide a significantly greater number of variations in terms of possible combinations and sequences of different metals, due the potential absence of local symmetry, and hence increased structural diversity, see Supporting Information for further discussion. The complexity of varying the components of a chain is illustrated by previous studies [37, 38, 39], notably a seminal contribution from Yaghi and coworkers who established the sequencing of metals within the chain secondary SBU of bimetallic MOF‐74 [37]. Yaghi's study illustrates the complexity observed even with a bimetallic multivariate MOF which incorporates a chain SBU. In our study, the complexity of the chain is significantly amplified by using more metals, up to 16 different metals, and hence increasing the number of possible sequences. We conclude that the structural diversity of multimetallic MTV‐MOFs is enhanced by studying systems containing chain SBUs in comparison to cluster SBUs.

### 2.2 Multivariate Compositional Control

Our studies demonstrated that although equimolar amounts of each metal were being used in the synthesis, the relative metal compositions of the frameworks do not mirror the initial reaction stoichiometry. Analyzing the EDX data, we found that the relative metal composition in the frameworks was inversely related to the ionic radius of the metal (Figure 5) [40]. That is to say that the smaller cations of the later lanthanides are present in a greater percentage in the final product probably due to their harder Lewis acidity. These results are in line with what was observed by Sikma et al. in their 15‐metal high entropy MOF containing Y and the entire lanthanide series (minus Pm) [19].

The relationship between reaction stoichiometry and relative composition was scrutinized in more detail by synthesizing the binary La/Lu system with varying ratios of La(NO_3_)_3_/Lu(NO_3_)_3_ in the reaction solutions, and EDX measurements of the relative compositions of the resulting MOFs (Figure 4). It was found that the smaller Lu ion has a three times greater likelihood for inclusion in the MOF as determined by the relative gradients of the lines (prior to 50% target input molar %), see Figure 4b. Thus, through a series of experiments we are able to determine a desired relative composition in a binary MOF and our approach was successfully demonstrated through the synthesis of a binary La/Lu MOF with 50:50% composition, **UoB‐116(La_0.5_Lu_0.5_)**, which requires a 3:1 La/Lu ratio in the reaction solution (see blue/green triangles in Figure 4b).

**FIGURE 4:**
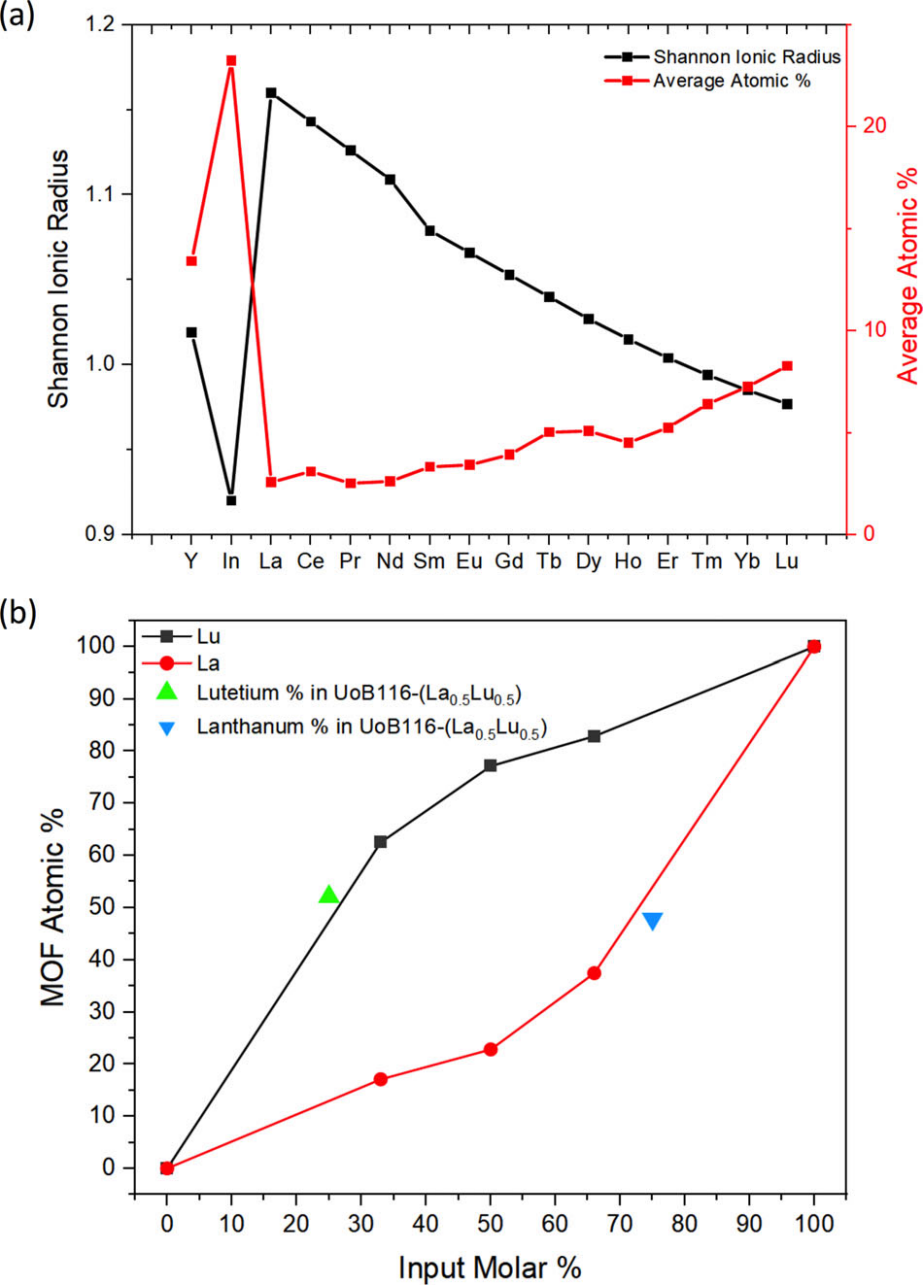
(a) The inverse relationship between the ionic radius and its percentage in the composition of the 16‐metal MTV‐MOF; (b) the effect of varying the input molar % of La(NO_3_)_3_ and Lu(NO_3_)_3_ on the relative metal composition of **UoB‐116(La_1‐x_Lu_x_)**. Blue and green triangles illustrate La/Lu concentrations required to prepare a 1:1 binary mixture in the resulting MTV‐MOF, **UoB‐116(La_0.5_Lu_0.5_)**.

In order to generalize our approach, we have found that a competitive binding isotherm approach can be used to control the relative metal composition. Assuming that the two metals compete for equivalent metal sites in the growing crystal, one can derive Equation (1). Where *X*_MOF_ represents the molar fraction of metal A in the MOF, *X*_sol_ likewise represents the molar fraction of metal A in the reaction solution and α represents the relative “binding affinity” of metal A relative to metal B which is determined empirically as demonstrated in Figure 4b. Upon determining the value of α, for the La/Lu system is ∼0.33, we were able to synthesize an approximately 50:50 composition bimetallic MOF by rearranging the design equation form, see Equation (2):

(1) $$X_{\mathrm{MOF}}=\frac{\alpha \;X_{\mathrm{sol}}}{1+\left(\alpha -1\right)X_{\mathrm{sol}}}$$

(2) $$X_{\mathrm{sol}}=\frac{X_{\mathrm{MOF}}}{\alpha -\left(\alpha -1\right)X_{\mathrm{MOF}}}$$

This equation reveals that a 3:1 ratio was required in the synthesis, and as such we were able to prepare a MOF with 52:48 La:Lu composition (Figure 4b). More broadly we believe this represents a methodology for the preparation of MTV‐MOFs of a desired metal composition and believe that it can extend to n metals through the transitivity property of related fractional quantities. That is to say that if one knows α for metal X and metal Y, and metal Y and metal Z, the α value for X relative to Z can be derived and empirical determination is not necessary in an ideal system. We are developing this methodology for the preparation of ternary and higher order MOFs with targeted compositions.

We probed the role of reaction time in determining MOF composition and assessed whether the composition is static once the framework has formed. In order to assess the effect of reaction time, a series of experiments were conducted in which the reaction time was varied between 24 and 192 h. It was found that the metal composition of the MOF did not change significantly despite the change in the length of reaction. This observation suggested that either, once formed, metals are unable to exchange with the MOF or that the composition of the MOF after 24 h is the thermodynamically favored product.

Thus, experiments were conducted to evaluate whether metal exchange was possible in this family. The monometallic La analogue was heated in 3 mL of a 16 mmol dm^−3^ solution of Lu(NO_3_)_3_ in DMF to investigate postsynthetic metal exchange. After 24 h of heating of the mixture, EDX was conducted on the dried material (Figure 5) and showed that Lu(III) was present particularly around cracks in the crystal of **UoB‐116(La_1‐x_Lu_x_)**, suggesting that Lu(III) is incorporated preferentially via particular faces of the MOF. Thus, it can be concluded that metals are exchangeable in this MTV‐MOF when introducing metals that lead to a thermodynamic product. It is also concluded that the composition observed from the initial 24 h reaction, is indeed the thermodynamic product for this family of MOFs.

**FIGURE 5:**
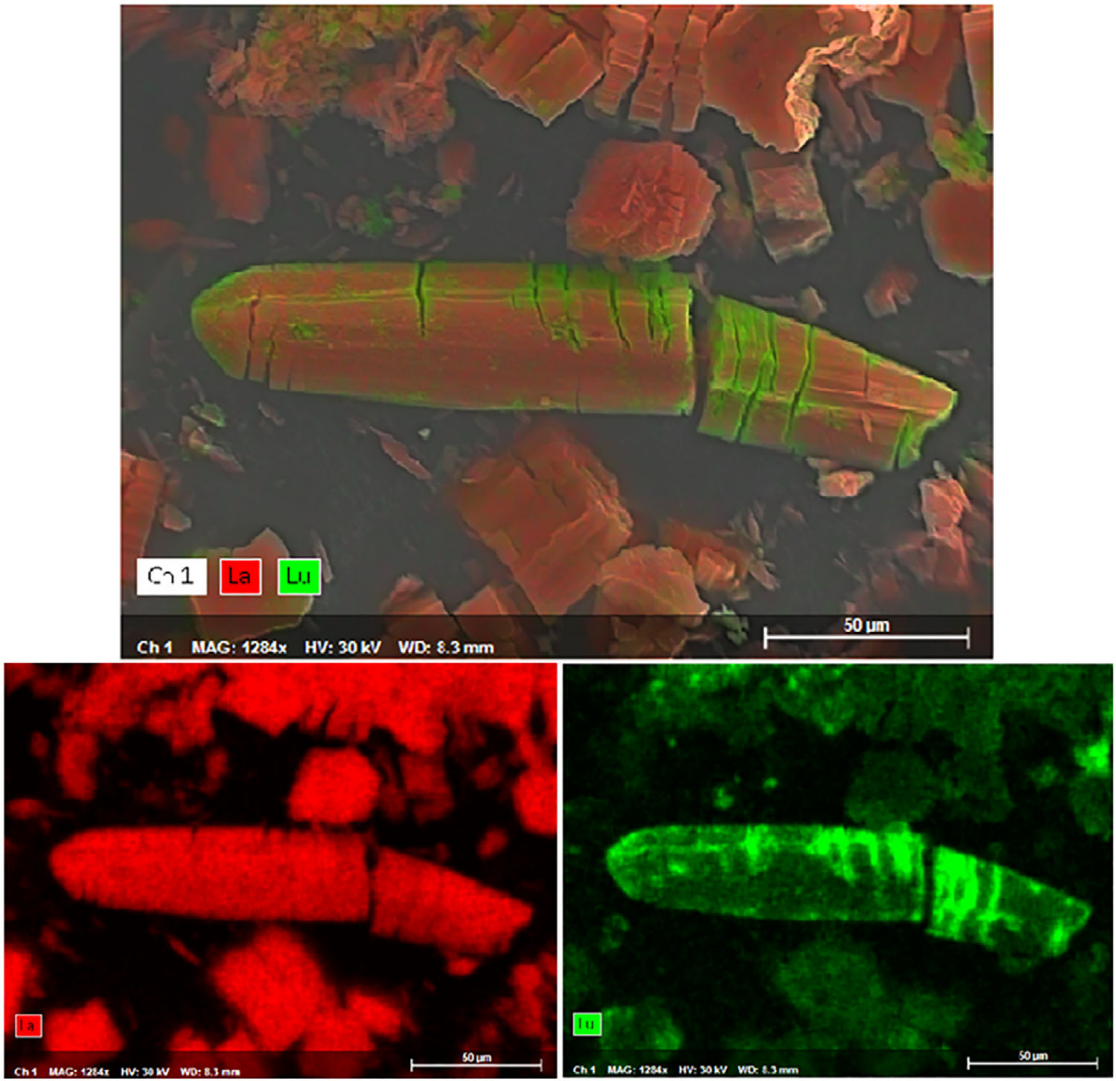
EDX maps of **UoB‐116(La)** following heating in a solution of Lu(NO_3_)_3_ confirming the presence of Lu in the MOF, particularly concentrated on the surface of the crystals. (La, red; Lu, green).

### 2.3 Impact of Multivariate Composition on Material Properties

Using the strategy to control the relative metal composition of **UoB‐116**, we prepared mixed **UoB‐116(Dy_1‐x_La_x_)** (where *x* = 0–1) MOFs, **UoB‐116(Dy_1_La_0_)**, **UoB‐116(Dy_0.82_La_0.18_)**, **UoB‐116(Dy_0.64_La_0.36_)**, **UoB‐116(Dy_0.36_La_0.64_)**, and **UoB‐116(Dy_0_La_1_)** (see Supporting Information), in order to probe the effect of composition on properties. As Dy content increases we see an increase in the intensity of an absorption in the NIR region at 805 nm, which was attributed to the ^6^H_15/2_→^6^F_5/2_ transition of the Dy^3+^ cation. The intensity of this peak can be tuned by Dy content of the material (Figure 6a), and inversely can be hindered by increasing La composition indicating the ability to tune the optical properties in **UoB116. UoB‐116** samples are photochromic (Figures S47 and S48), as anticipated for NDI‐containing framework materials [41, 42], but we found that the response was independent of the metal composition of the material, indicating a ligand‐based photochromism.

**FIGURE 6:**
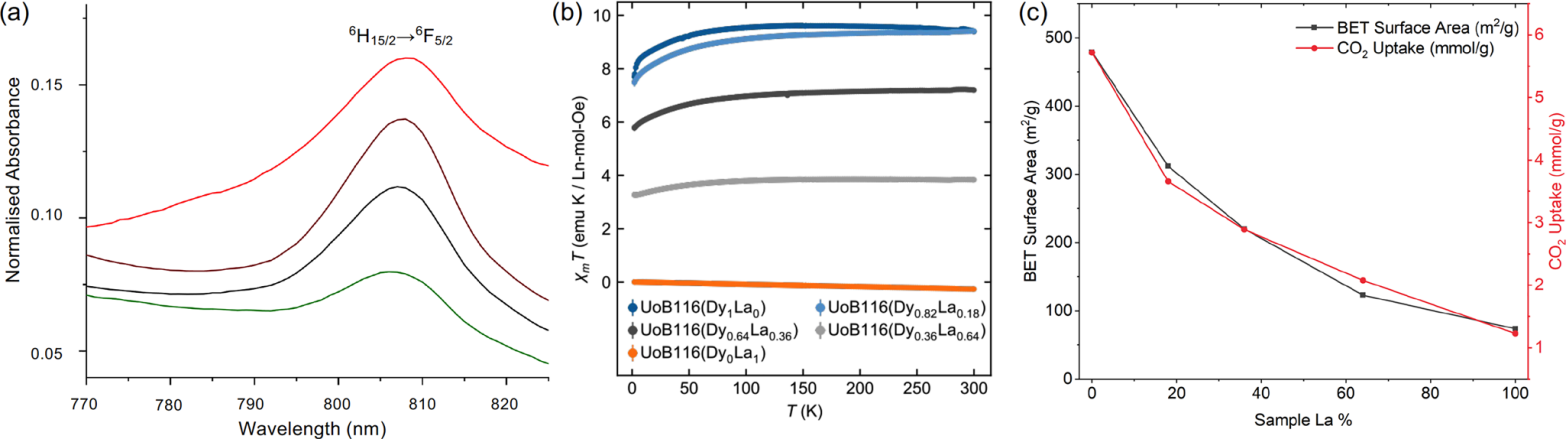
Properties of **UoB‐116(Dy_1‐x_La_x_)** MOFs, **UoB‐116(Dy_1_La_0_)**, **UoB‐116(Dy_0.82_La_0.18_)**, **UoB‐116(Dy_0.64_La_0.36_)**, **UoB‐116(Dy_0.36_La_0.64_)**, and **UoB‐116(Dy_0_La_1_)**. (a) UV–visible spectra showing increasing intensity of the ^6^H_15/2_→^6^F_5/2_ transition with increasing Dy content (red—**UoB‐116(Dy_1_La_0_)**; brown—**UoB‐116(Dy_0.82_La_0.18_)**; blue—**UoB‐116(Dy_0.64_La_0.36_)**; green—**UoB‐116(Dy_0.36_La_0.64_)**); (b) temperature dependence of the magnetic susceptibility of the **UoB‐116(Dy_1‐x_La_x_)** MOFs measured under an applied magnetic field of 100 Oe, product of magnetic susceptibility and temperature, *χ*_m_ *T*, normalized per mole of total lanthanide (Ln); (c) changes in BET surface area (red) and CO_2_ uptake (black) (195 K, 1 bar) as a function of La doping level).

The magnetic properties of the **UoB‐116(Dy_1‐x_La_x_)** MOFs were also investigated by temperature‐dependent magnetic susceptibility measurements. As the proportion of Dy in **UoB‐116** increases the magnetic susceptibility of the MOF increases (Figure 6b) such that the calculated effective magnetic moments, *μ*_calc_, increase **UoB‐116(Dy_0_La_1_)** < **UoB‐116(Dy_0.36_La_0.64_)** < **UoB‐116(Dy_0.64_La_0.36_)** < **UoB‐116(Dy_0.82_La_0.18_)** < **UoB‐116(Dy_1_La_0_)** (Table S2). These results indicate that controlling the metal content of **UoB‐116** MOFs can be used to directly tune the magnetic properties of the MOF.

To investigate the effects of metal composition on the porosity of **UoB‐116**, we systematically investigated the five **UoB‐116(Dy_1‐x_La_x_)** analogues, described above, with varying levels of La doping (*x* = percentage of La substitution). Thermogravimetric analysis confirmed that the occluded DMF molecules can be removed at 60°C under N_2_ flow (Figures S7–S16). Variable temperature PXRD patterns confirmed that the MOFs remain crystalline, retaining the same framework structure, upon heating to this activation temperature, 60°C (Figures S17–S21). MOFs containing Dy show some changes in the observed reflections when heated above 60°C, although it is notable that a temperature over 200°C is required for **UoB‐116(Dy_0_La_1_)** to undergo changes in the diffraction pattern. This behavior suggests a degree of structural flexibility at elevated temperatures for the UoB‐116 family, with the behavior dependent on the metal content of the specific MOF. Importantly, the activated sorbents, evacuated at 60°C, retain the respective parent structures. The activated samples exhibited a Type‐I CO_2_ adsorption isotherm at 195 K. The CO_2_ uptake at 1 bar decreased progressively with increasing La content, from 5.72, 3.66, 2.88, and 2.08 to 1.23 mmolg^−1^ for **UoB‐116(Dy_1_La_0_)**, **UoB‐116(Dy_0.82_La_0.18_)**, **UoB‐116(Dy_0.64_La_0.36_)**, **UoB‐116(Dy_0.36_La_0.64_)**, and **UoB‐116(Dy_0_La_1_)** respectively (Figures S22–S26). A similar trend was observed for the Brunauer–Emmett–Teller (BET) surface areas, which decreased from 479 m^2^ g^−1^ for **UoB‐116(Dy_1_La_0_)** (Figure S27) to 312, 220, 123, and 74 m^2^ g^−1^ for **UoB‐116(Dy_0.82_La_0.18_)**, **UoB‐116(Dy_0.64_La_0.36_)**, **UoB‐116(Dy_0.36_La_0.64_)**, and **UoB‐116(Dy_0_La_1_)** respectively (Figures S28–S31). To further probe the CO_2_–framework interactions, CO_2_ adsorption isotherms were recorded at 273, 283, and 298 K (Figures S32–S36), and isosteric enthalpies of adsorption (*Q*_st_) were computed using the Clausius–Clapeyron equation (Figures S37–S41). The low coverage *Q*_st_ values were determined to be 20.6, 23.5, 22.4, 26.6, and 22.5 kJ mol^−1^ for **UoB‐116(Dy_1_La_0_)**, **UoB‐116(Dy_0.82_La_0.18_)**, **UoB‐116(Dy_0.64_La_0.36_)**, **UoB‐116(Dy_0.36_La_0.64_)**, and **UoB‐116(Dy_0_La_1_)** respectively, indicating moderate CO_2_‐framework interactions (Figures S42–S46). Overall, these results demonstrate that La doping provides an effective strategy for fine‐tuning the porosity and CO_2_ adsorption properties of **UoB‐116(Dy_1‐x_La_x_)** series, enabling systematic modulation of both BET surface area and CO_2_ affinity (Figure 6c).

## 3 Conclusions

In conclusion, a family of MTV‐MOFs has been prepared with a wide variety of metal compositions. In addition to 15 monometallic analogues of **UoB‐116(Ln)** (where Ln = Y, La, Ce, Pr, Nd, Sm, Eu, Gd, Tb, Dy, Ho, Er, Tm, Yb, and Lu) which were prepared and shown to be isoreticular, MTV‐MOFs with 2, 4, 12, 15, and 16 (including In(III)) different metals were synthesized. SCXRD data were collected for the 12 and 16 metal MTV‐MOFs which confirmed the same structure as the monometallic MOFs. EDX analysis confirmed the composition of the MTV‐MOFs and revealed preferential incorporation of metal cations with smaller ionic radii. This observation was leveraged to produce bimetallic MOFs of a targeted composition, **UoB‐116(La_x_Lu_y_)**, and **UoB‐116(Dy_x_La_y_)**, a strategy which could be extended, in the future, to other bimetallic systems of this MOF, or even to control the composition of systems with more than two metals. Further, a systematic study of five **UoB‐116(Dy_1‐x_La_x_)** analogues establish La doping as a simple and effective means of systematically fine‐tuning microporosity and CO_2_ adsorption, as well as magnetic properties. The preferential incorporation of metals with smaller ionic radii can be further used for the development of postsynthetically modified materials with introduction of a secondary metal on the surface of MOF crystals.

Our study not only demonstrates the synthesis of MTV‐MOFs with the highest yet reported number of different metals, 16, but also incorporates metals from the *f*‐, *d*‐, and *p*‐blocks, to our knowledge the first example of such a system. The ability to control the relative composition of bimetallic MOFs introduces the potential for compositional control for larger numbers of different metals from across the periodic table and also for postsynthetic metalation to create MTV‐MOFs. Furthermore, we demonstrate that combining different metal cations in **UoB‐116** results in the ability to tune the properties (porosity, magnetism, and UV–visible absorption) of the resulting MOFs, confirming that the potential of using the **UoB‐116** multivariate MOF family as a tuneable platform.

## Author Contributions

The synthesis of materials and other experimental studies were conducted by A.I., J.O.O., and M.M.M. A.I., J.O.O., M.M.M., D.E.S., and S.P.A. collected and analyzed the SCXRD data. J.L.B. and L.C. recorded and analyzed magnetic data. A.R. and S.M. recorded and analyzed porosity, variable‐temperature diffraction, and thermal stability data. The project was conceived and supervised by N.R.C. All authors discussed the results, contributed to and have given approval to the final version of the manuscript.

## Conflicts of Interest

The authors declare no conflicts of interest.

## Supporting information




**Supporting File**: Data supporting the conclusions drawn in the main text are contained in the Supporting Information file.

## Data Availability

The authors declare that all characterization data generated in this study are provided in the Supporting Information or within the main manuscript. The x‐ray crystallographic coordinates for structures reported in this study have been deposited at the Cambridge Crystallographic Data Centre (CCDC), under deposition numbers 2458698 (Pr), 2458741 (Eu), 2458813 (Gd), 2458829 (Tb), 2458871 (Tm), 2472917 (Dy), 2472918 (Ho), 2472919 (Ce), 2472920 (Nd), 2472921 (La), 2472926 (Er), 2472927 (Lu), 2472928 (Sm), 2472929 (Yb), 2472931 (Y), 2472932 (Eu/Tb), 2472933 (16 Metal), 2472983 (12 Metal). These data can be obtained free of charge from The Cambridge Crystallographic Data Centre via www.ccdc.cam.ac.uk/data_request/cif. Crystallographic information files (CIF). All other data supporting the findings of this study are available from the article and the supplemental information or from the lead contact upon reasonable request.
