# Supplementary material for: Rare‐Earth Multivariate Metal–Organic Frameworks: Cationic Radius Biased Compositional Control and Property Tuning
Source: Angew Chem Int Ed Engl. 2026 Sep 26;65(46):e5287849. doi: 10.1002/anie.5287849 (PMC13749303; doi:10.1002/anie.5287849)
Supplement: Supplementary file 1 [file ANIE-65-e5287849-s001.pdf]

*Supplementary Information for:*

# Rare-earth Multivariate Metal-organic Frameworks : Cationic Radius Biased Compositional Control and Property Tuning

Adnan Ishaq,[a] Joseph O. Ogar,[b] Asif Raza,[c] Musa M. Mahmud,[a] Jacob L. Brownlee,[a] Danielle E. Schier,[a] Stephen P. Argent,[b] Lucy Clark,[a] Soumya Mukherjee,[c] and Neil R. Champness[a]*

[a] [a] Adnan Ishaq, Dr. Musa M. Mahmud, Jacob L. Brownlee, Dr. Danielle E. Schier, Prof. Lucy Clark, Prof. Neil R. Champness
School of Chemistry, University of Birmingham, Edgbaston, Birmingham, B15 2TT, UK.
E-mail: n.champness@bham.ac.uk

[b] Dr. Joseph O. Ogar, Stephen P. Argent,
School of Chemistry, University of Nottingham, University Park, Nottingham, NG7 2RD, UK.

[c] Dr. Asif Raza, Prof. Soumya Mukherjee
Bernal Institute and Research Ireland Centre for Pharmaceuticals (SSPC), Department of Chemical Sciences, University of Limerick, Limerick V94 T9PX, Ireland.

**Supporting Information Table of Contents**

Experimental Details including Synthesis Page 2

EDX Characterisation Page 4

Single-Crystal X-ray Diffraction studies Page 8

Composition of UoB-116($Dy_xLa_y$) MOFs Page 11

Stability and Porosity Studies Page 12

UV-visible Spectroscopy and Photochromism Studies Page 39

Magnetism Studies Page 40

Cluster vs. Chains Page 42

References Page 42

## Experimental

### Materials

5-aminonicotinic acid, naphthalenetetracarboxylic dianhydride and all metal salts were purchased from either Sigma Aldrich, Alpha Aesar or Fluorochem and were used without further purification.

*Synthesis of N,N'-bis(dinicotinic acid)-1,4,5,8-naphthalenetetracarboxydiimide ($H_2$DNNDI)*

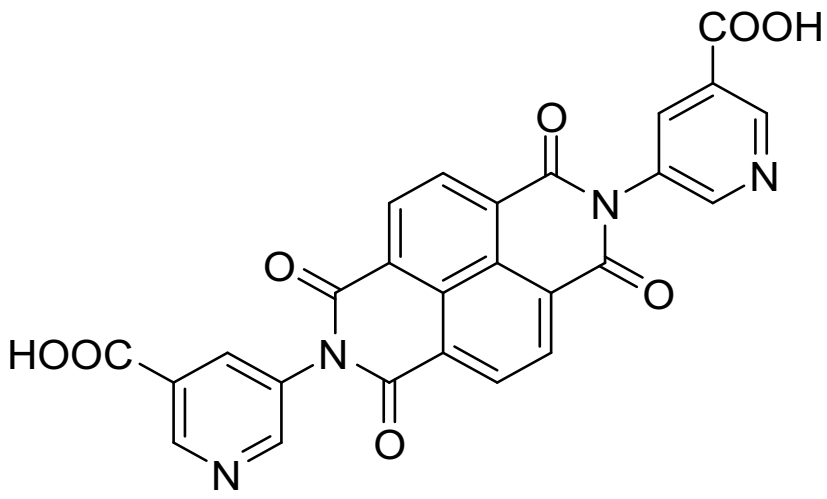


$H_2$DNNDI was synthesised by the following procedure. To a 50 mL round bottom flask containing 20 mL DMF, 5-aminonicotinic acid (915 mg, 7.5 mmol) was added and stirred at 100 °C for 15 minutes. To this suspension, 1,4,5,8-naphthalenetetracarboxylic dianhydride (NTCDA) (800 mg, 3.0 mmol) was added which gives rise to a brown solution as the products react. After 24 hours the reaction was allowed to cool to room temperature, and the solid component was filtered under vacuum. The beige powder collected was subjected to 3 boiling filtrations in DMF to remove any residual impurities which is an essential step for the subsequent MOF synthesis. After this, the material was washed with acetone and diethyl ether to obtain an off-white powder with a yield of 65% and with purity sufficient for the facile synthesis of MOF crystals suitable for SCXRD characterisation. (0.99 g, 1.95 mmol). $^1$H NMR (400 MHz; DMSO-$d_6$) δ=13.71 (s, 2H), 9.19 (d, J=1.8,2H), 8.92 (d, J=2.3, 2H)8.78 (s, 4H)8.52(m,2H),7.96(s,2H)

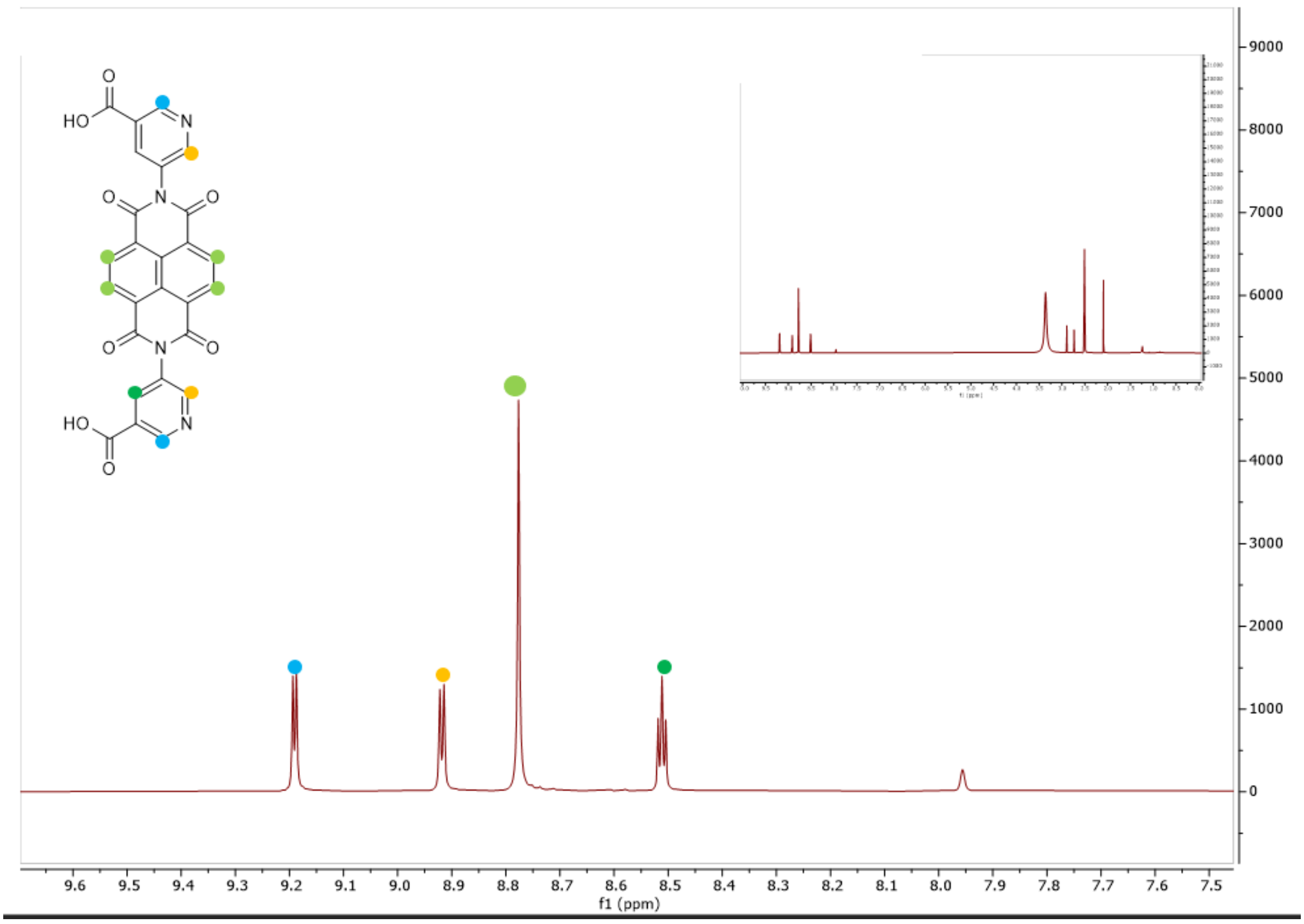


**Figure S1**. $^{1}H$ NMR spectrum of $H_2DNNDI$ in DMSO-$d_6$.

*General synthesis of **UoB-116(Ln)***

All single metal examples of **UoB-116(Ln)** were synthesised by the following procedure. Before any reagents were added, a 20mL disposable scintillation vial was siliconized by applying Sigmacote which was then allowed to evaporate. Any remaining HCl by-product was washed out with 10mL deionised water and 3 x 10 mL acetone before drying for 30 minutes in an oven set to 60 °C. To the vial 0.034 mmol of $Ln(NO_3)_3$ was added followed by 17mg (0.034 mmol) $H_2DNNDI$ ligand and 3 mL DMF. The reaction mixture was sonicated for 90 seconds before being placed in an oven set to 100 °C for 24 hours. Upon removal, large needle shaped crystals suitable for SCXRD analysis were obtained and washed 3 times with 10 mL DMF.

*Synthesis of **UoB-116($La_{0.25}Lu_{0.75}$)***

**UoB-116($La_{0.25}Lu_{0.75}$)** was synthesised in the same fashion described in the general synthesis. 5.5 mg of $La(NO_3)_3$ and 6.2 mg $Lu(NO_3)_3$ was added to the reaction vial.

*Synthesis of* ***UoB-116(La$_{0.5}$Lu$_{0.5}$)***

**UoB-116(La$_{0.5}$Lu$_{0.5}$)** was synthesised in the same fashion described in the general synthesis. 8.3 mg (0.0255 mmol) of La($NO_3$)$_3$ and 3.1 mg, (0.0085 mmol) Lu($NO_3$)$_3$ was added to the reaction vial.

*Synthesis of* ***UoB-116(La,Ce,Pr,Nd,Eu,Gd,Tb,Dy,Ho,Er,Yb,Lu)***

**UoB-116(La,Ce,Pr,Nd,Eu,Gd,Tb,Dy,Ho,Er,Yb,Lu)** was synthesised in the same fashion described in the general synthesis however, 1/12$^{th}$ of an equivalent of each rare earth metal nitrate was added to the reaction vial (0.00283 mmol). Below are the EDX spectra confirming presence of all metals in the framework.

*Synthesis of* ***UoB-116(Y,La,Ce,Pr,Nd,Sm,Eu,Gd,Tb,Dy,Ho,Er,Tm,Yb,Lu)***

**UoB-116(Y,La,Ce,Pr,Nd,Sm,Eu,Gd,Tb,Dy,Ho,Er,Tm,Yb,Lu)** was synthesised in the same fashion described in the general synthesis however, 1/15$^{th}$ of an equivalent of each rare earth metal nitrate was added to the reaction vial (0.00267 mmol). Below are the EDX spectra confirming presence of all metals in the framework.

*Synthesis of* ***UoB-116(Y,In,La,Ce,Pr,Nd,Sm,Eu,Gd,Tb,Dy,Ho,Er,Tm,Yb,Lu)***

**UoB-116(Y,In,La,Ce,Pr,Nd,Sm,Eu,Gd,Tb,Dy,Ho,Er,Tm,Yb,Lu)** was synthesised in the same fashion as described in the general synthesis however, 1/16$^{th}$ of an equivalent of each rare earth metal nitrate salt was added to the reaction vial (0.00213mmol). Indium (III) chloride was used as the source of indium$^{3+}$ (0.00213 mmol, 0.417 mg). Below are the EDX spectra confirming presence of all metals in the framework.

*Synthesis of* ***UoB-116(Dy$_x$La$_y$)***

La/Dy samples were synthesised by using varying ratios (100%:0%, 75%:25%, 50%:50%, 25%:75% and 0%:100%) of La($NO_3$)$_3$ and Dy($NO_3$)$_3$ on a 5x larger scale synthesis compared to the usual synthesis conditions described above. 20 mL scintillation vials were replaced with 50 mL MOF synthesis jars for these larger scale batches.

**EDX Characterisation**

Energy dispersive X-ray spectroscopy (EDX) was used to characterise the relative metal compositions of the mixed-metal MOFs produced. A JEOL-6060 microscope was used with Oxford Instruments EDX detector running on Oxford INCA software. Crystals were dried by vacuum desiccation before being mounted on carbon-conductive tabs uncoated. Spectra were collected at an accelerating voltage of 30 kV and spot size of 76 with a working distance of 10 mm. Acquisition live times of between 300 and 600 seconds were used to ensure appropriate signal to noise ratios which was particularly important for the mixed-metal systems with larger numbers of metals. Each composition was measured on at least 3 different crystals.

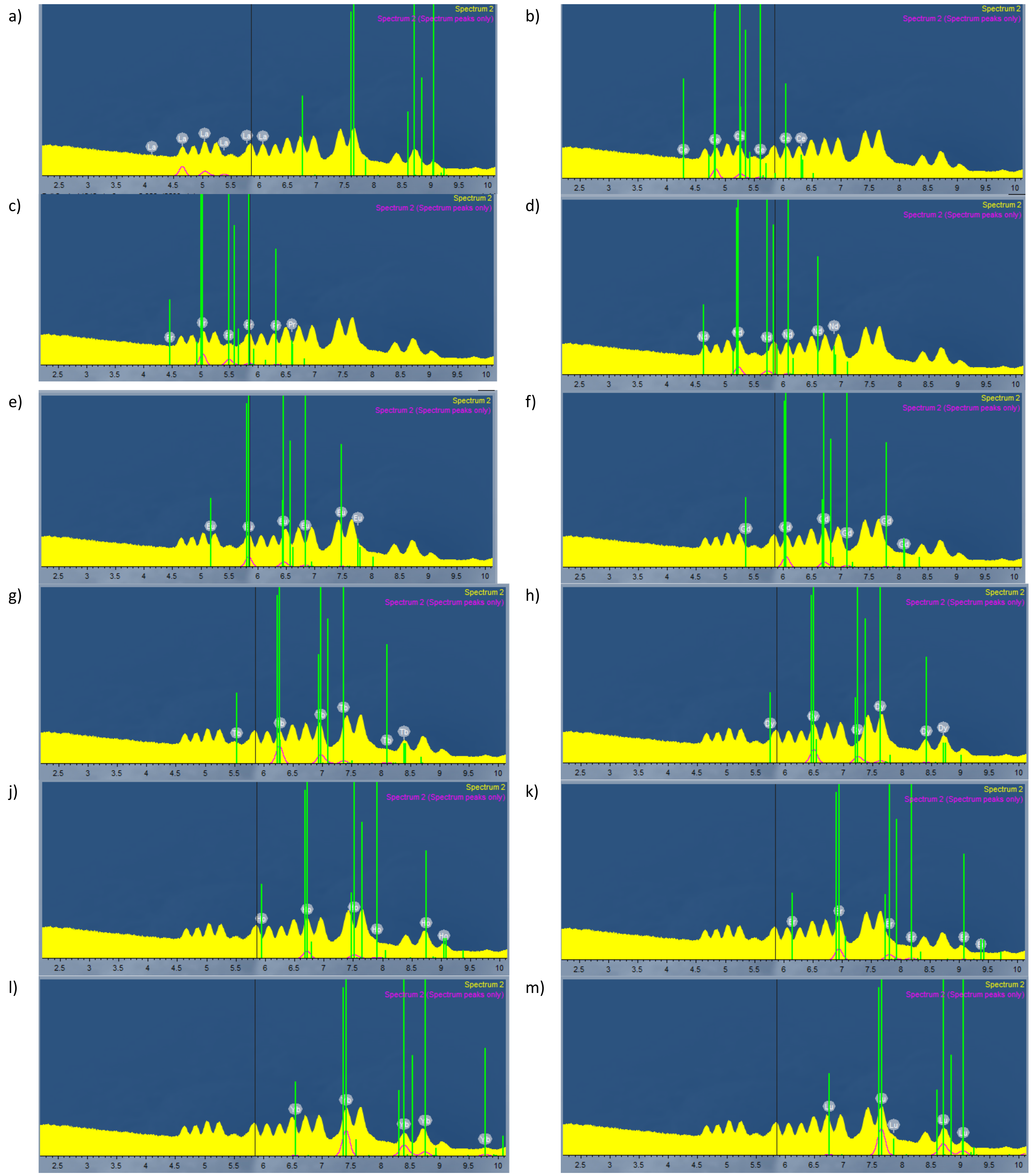


**Figure S2**. EDX Spectra for **UoB-116(La,Ce,Pr,Nd,Eu,Gd,Tb,Dy,Ho,Er,Yb,Lu)**; a) La, b) Ce, c) Pr, d) Nd, e) Eu, f) Gd, g) Tb, h) Dy, j) Ho, k) Er, l) Yb, m) Lu.

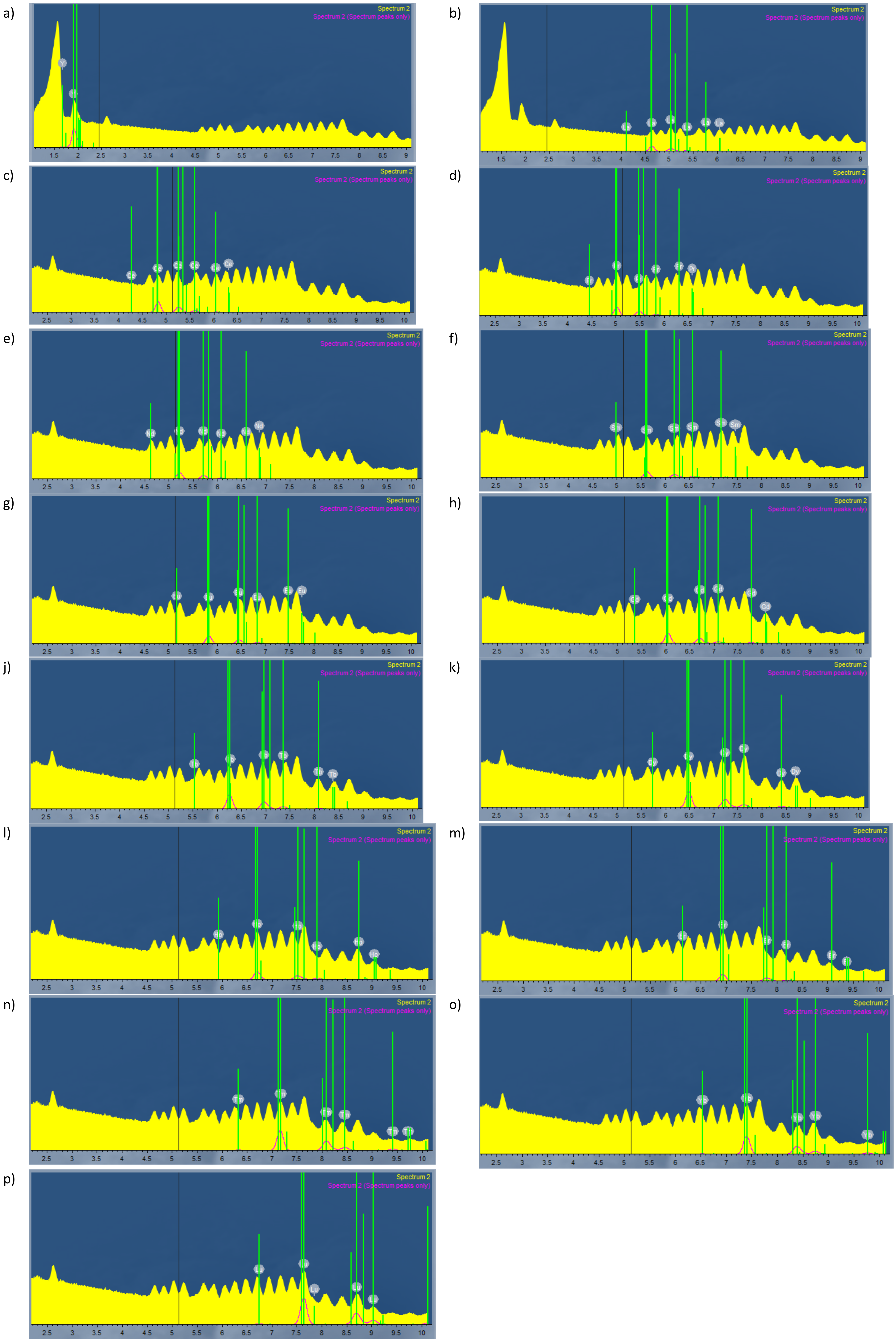

**Figure S3**. EDX Spectra for **UoB-116(Y,La,Ce,Pr,Nd,Sm,Eu,Gd,Tb,Dy,Ho,Er,Tm,Yb,Lu)**; a) Y, b) La, c) Ce, d) Pr, e) Nd, f) Sm, g) Eu, h) Gd, j) Tb, k) Dy, l) Ho, m) Er, n) Tm, o) Yb, p) Lu.

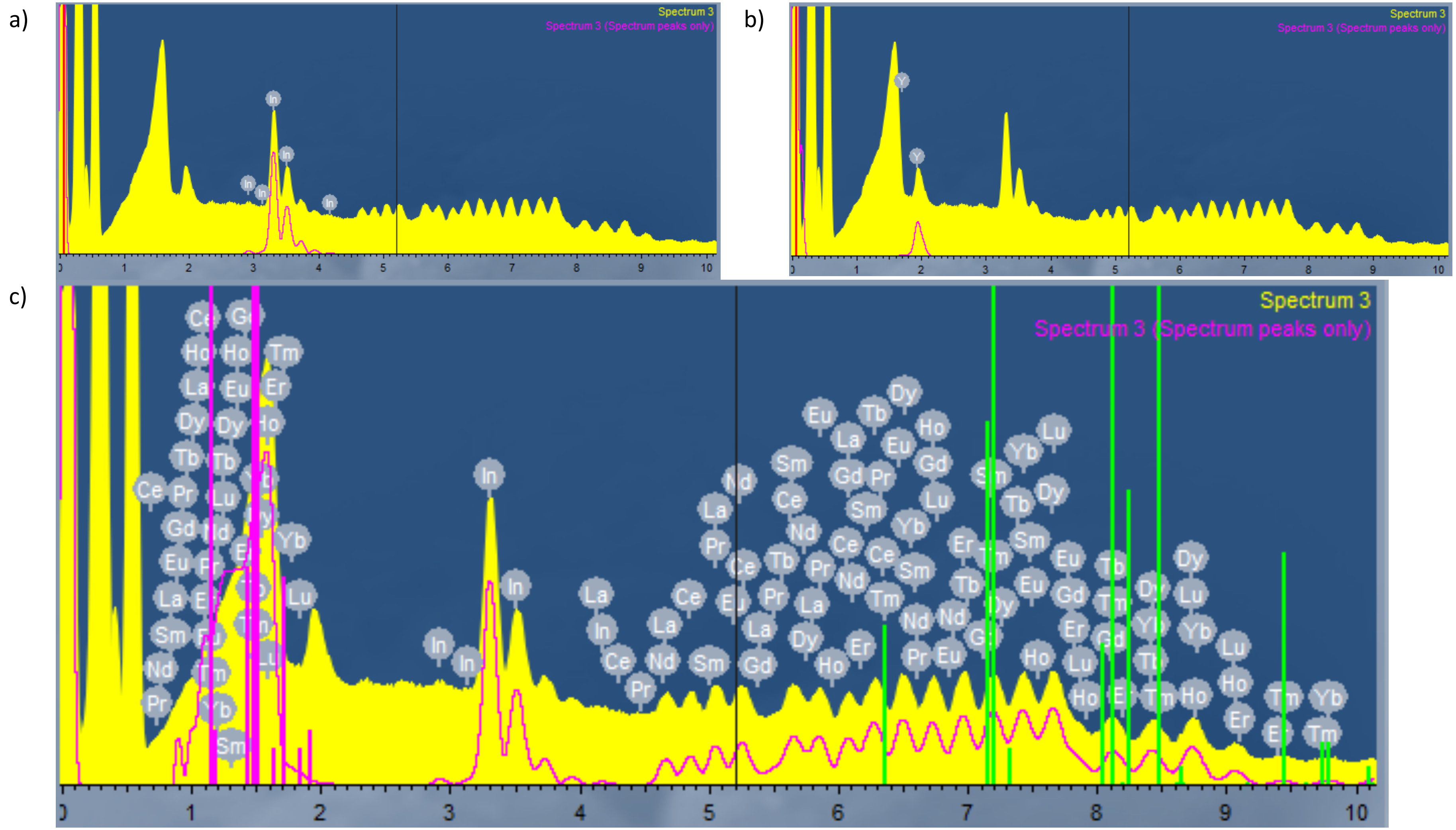


**Figure S4**. EDX Spectra for **UoB-116(Y,In,La,Ce,Pr,Nd,Sm,Eu,Gd,Tb,Dy,Ho,Er,Tm,Yb,Lu)**; a) In, b) Y, c) full spectrum, Tm highlighted.

**Single-Crystal X-ray Diffraction studies**

For **UoB-116(Pr)**, **UoB-116(Nd)**, **UoB-116(Eu)**, **UoB-116(Gd)**, **UoB-116(Tb)**, **UoB-116(Dy)**, **UoB-116(Er)**, **UoB-116(Tm)**, **UoB-116(Yb)**, **UoB-116(Lu)**, **UoB-116(Eu,Tb)** single-crystal diffraction data for structure determination were collected on a Rigaku XtaLAB Synergy-S X-ray diffractometer equipped with a HyPix-6000 hybrid photon counting detector and a Mo (or Cu for **UoB-116(Lu)**) microfocus source. Single crystal X-ray diffraction experiments were performed on a XtalLAB PRO MM007, PILATUS3 R 200K diffractometer for **UoB-116(Ce)**, on a Rigaku Oxford Diffraction SuperNova GV1000 diffractometer using mirror monochromated Cu Kα radiation and Titan S2 CCD detector for **UoB-116(Ho)**, and a Rigaku R-AXIS SPIDER iP diffractometer using Mo Kα radiation for **UoB-116(Y)** and **UoB-116(Sm).** In each case a suitable crystal was isolated and mounted on a MiTeGen loop in a droplet of a perfluoropolyether oil before being mounted onto the goniometer under a 100 K cryostream.

For **UoB-116(La)**, **UoB-116(La,Ce,Pr,Nd,Eu,Gd,Tb,Dy,Ho,Er,Yb,Lu)** and **UoB-116(Y,In,La,Ce,Pr,Nd,Sm,Eu,Gd,Tb,Dy,Ho,Er,Tm,Yb,Lu)** single crystal diffraction data were collected in EH1 of Diamond Light Source beamline I19. The data were collected at a wavelength of 0.6889 Å on a Fluid Film Devices 3-circle fixed-chi diffractometer using a Dectris Pilatus 2M detector. The collected frames were integrated using XIA26 software or CrysAlisPro software (1.171.39.46 Rigaku Oxford Diffraction, 2018). The crystals were mounted on MiTeGen micromounts using a perfluoropolyether oil, where they were cooled for data collection by a Cryostream nitrogen-gas stream. The data was processed using CrysAlisPro software (1.171.39.46 Rigaku Oxford Diffraction, 2018) and the corrected for absorption empirically using spherical harmonics, implemented in SCALE3 ABSPACK. The crystals were kept at 100(2) K during data collection.

Using Olex2,[S1] the structures were solved with the ShelXT[S2] structure solution program using Intrinsic Phasing and refined with the ShelXL[S3] refinement package using Least Squares minimisation. In the cases of the single crystal data for **UoB-116(Eu,Tb)**, **UoB-116(La,Ce,Pr,Nd,Eu,Gd,Tb,Dy,Ho,Er,Yb,Lu)** and **UoB-116(Y,In,La,Ce,Pr,Nd,Sm,Eu,Gd,Tb,Dy,Ho,Er,Tm,Yb,Lu)**, the metal site was refined as a fully occupied Gd(III) cation which represented the average atomic mass of the metals used in each MOF. In all cases a solvent mask was used to allow for pore solvent molecules, although in some instances DMF solvent molecules could be located and modelled in the MOF pores. For specific details of modelling, see the CIF entries for each structure.

**Table S1**.

| MOF | No. of Metals | Space Group | a | b | c | α | β | γ | r1 | wr2 | CCDC No. |
|---|---|---|---|---|---|---|---|---|---|---|---|
| **UoB-116(Y)** | **1** | **$I4_1/a$** | 38.6590(2) | 38.6590(2) | 16.0809(1) | 90° | 90° | 90° | 5.15 | 14.60 | 2472931 |
| **UoB-116(La)** | **1** | **I2/a** | 39.0913(3) | 16.5724(1) | 39.2585(7) | 90° | 90° | 90° | 9.34 | 28.23 | 2472921 |
| **UoB-116(Ce)** | **1** | **$I4_1/a$** | 38.3333(6) | 38.3333(6) | 16.3408(4) | 90° | 90° | 90° | 6.71 | 20.09 | 2472919 |
| **UoB-116(Pr)** | **1** | **$I4_1/a$** | 38.7750(8) | 38.7750(8) | 16.3445(5) | 90° | 90° | 90° | 5.26 | 14.75 | 2458698 |
| **UoB-116(Nd)** | **1** | **C2/c** | 57.821(11) | 16.3961(5) | 38.836(7) | 90° | 137.74(3)° | 90° | 10.40 | 30.60 | 2472920 |
| **UoB-116(Sm)** | **1** | **I2/a** | 39.1295(6) | 16.3070(1) | 38.8870(5) | 90° | 95.293(1)° | 90° | 9.37 | 27.02 | 2472928 |
| **UoB-116(Eu)** | **1** | **$I4_1/a$** | 38.3617(3) | 38.3617(3) | 16.1710(2) | 90° | 90° | 90° | 5.57 | 14.14 | 2458741 |
| **UoB-116(Gd)** | **1** | **C2/c** | 52.3661(9) | 16.26957(10) | 39.2319(7) | 90° | 132.224(3)° | 90° | 3.96 | 11.13 | 2458813 |
| **UoB-116(Tb)** | **1** | **$I4_1/a$** | 38.4213(2) | 38.4213(2) | 16.0164(1) | 90° | 90° | 90° | 4.17 | 11.85 | 2458829 |
| **UoB-116(Dy)** | **1** | **$I4_1/a$** | 38.6806(3) | 38.6806(3) | 16.1012(1) | 90° | 90° | 90° | 6.34 | 17.31 | 2472917 |
| **UoB-116(Ho)** | **1** | **$I4_1/a$** | 38.5733(5) | 38.5733(5) | 16.0429(3) | 90° | 90° | 90° | 4.93 | 11.46 | 2472918 |
| **UoB-116(Er)** | **1** | **$I4_1/a$** | 38.5487(3) | 38.5487(3) | 16.03609(18) | 90° | 90° | 90° | 6.12 | 16.00 | 2472926 |
| **UoB-116(Tm)** | **1** | **$I4_1/a$** | 38.5851(5) | 38.5851(5) | 16.1216(3) | 90° | 90° | 90° | 5.46 | 17.24 | 2458871 |
| **UoB-116(Yb)** | **1** | **$I4_1/a$** | 38.6305(2) | 38.6305(2) | 16.0212(1) | 90° | 90° | 90° | 3.66 | 9.45 | 2472929 |
| **UoB-116(Lu)** | **1** | **$I4_1/a$** | 38.8124(5) | 38.8124(5) | 16.0032(3) | 90° | 90° | 90° | 4.93 | 13.86 | 2472927 |
| **UoB-116(Eu,Tb)** | **2** | **$I4_1/a$** | 39.0280(12) | 39.0280(12) | 16.2441(7) | 90° | 90° | 90° | 8.61 | 26.27 | 2472932 |
| **UoB-116(Ln)** | **12** [a] | **$I4_1/a$** | 38.306 | 38.306 | 16.111 | 90° | 90° | 90° | 3.18 | 9.47 | 2472983 |
| **UoB-116(Ln)** | **16** [b] | **C2/c** | 54.1792(13) | 16.1560(3) | 38.9622(13) | 90° | 133.840(2)° | 90° | 11.35 | 30.87 | 2472933 |

[a] where Ln = La, Ce, Pr, Nd, Eu, Gd, Tb, Dy, Ho, Er, Yb, Lu; [b] where Ln = In, Y, La, Ce, Pr, Nd, Sm, Eu, Gd, Tb, Dy, Ho, Er, Tm, Yb, Lu

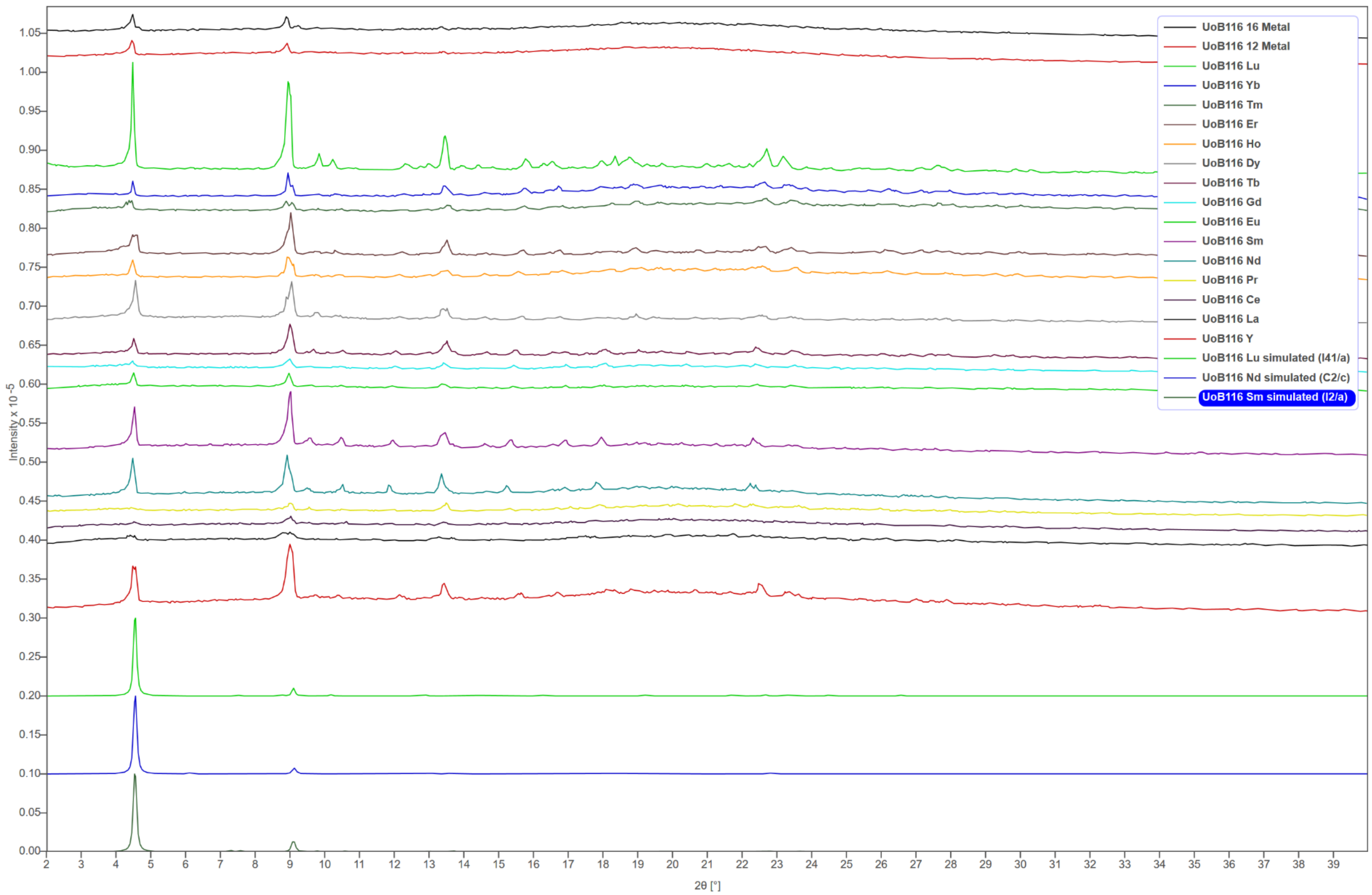


**Figure S5**. Powder X-ray diffractograms for all **UoB-116** samples including, **UoB-116(La,Ce,Pr,Nd,Eu,Gd,Tb,Dy,Ho,Er,Yb,Lu)** and **UoB-116(Y,In,La,Ce,Pr,Nd,Sm,Eu,Gd,Tb,Dy,Ho,Er,Tm,Yb,Lu)**.

**Composition of UoB-116($Dy_{1-x}La_x$) MOFs.**

Samples of **UoB-116($Dy_{1-x}La_x$)** (x = 0-1) were prepared as described above. Employing our competitive binding isotherm approach, described in the main text, we were able to control the relative metal composition of **UoB-116($Dy_{1-x}La_x$)**. Figure S6 shows the effect of varying the input molar % of $La(NO_3)_3$ and $Dy(NO_3)_3$ on the relative metal composition of **UoB-116($Dy_{1-x}La_x$)**. From the plot it can be seen that the relatively similar cationic radii of $Dy^{3+}$ and $La^{3+}$ (in comparison to the larger difference between the cationic radii of $La^{3+}$ and $Lu^{3+}$) results in a smaller thermodynamic driving force to incorporate one metal over the other in **UoB-116($Dy_{1-x}La_x$)**.

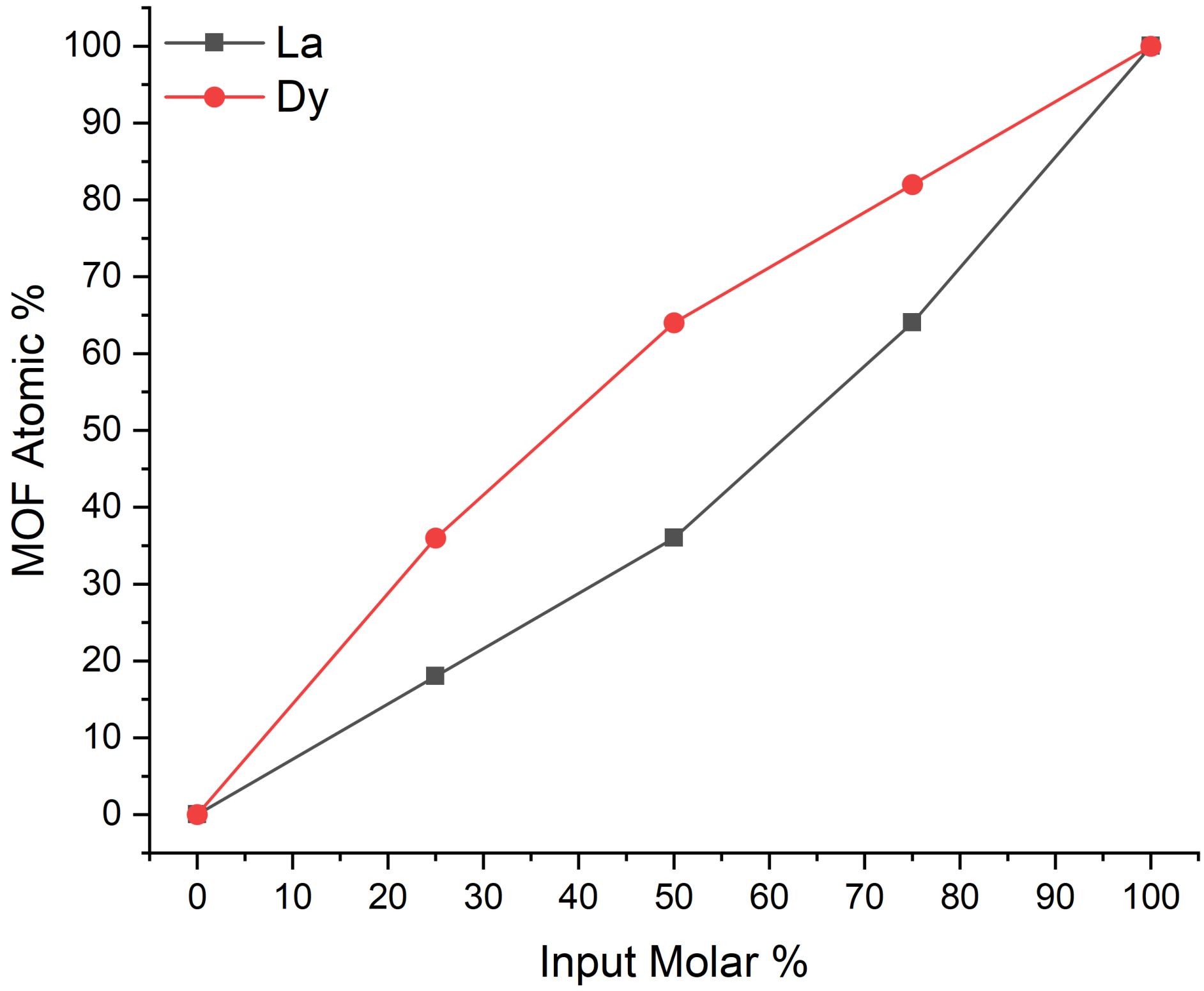


**Figure S6**. The effect of varying the input molar % of $La(NO_3)_3$ and $Dy(NO_3)_3$ on the relative metal composition of **UoB-116($Dy_{1-x}La_x$)**.

**Stability and Porosity Studies**

**Thermogravimetric analysis (TGA)**

Thermograms were recorded under nitrogen using TGA instrument Discovery TGA 55 of Waters™. Aluminium pans and a flow rate of 40 $cm^3$ $min^{-1}$ for $N_2$ gas were used for the experiments. The data was collected in the High-Resolution Dynamic mode with a sensitivity of 1.0, a resolution of 4.0, and a temperature ramp of 2 °C $min^{-1}$ up to 550 °C. The data was evaluated using the T.A. Universal Analysis suite.

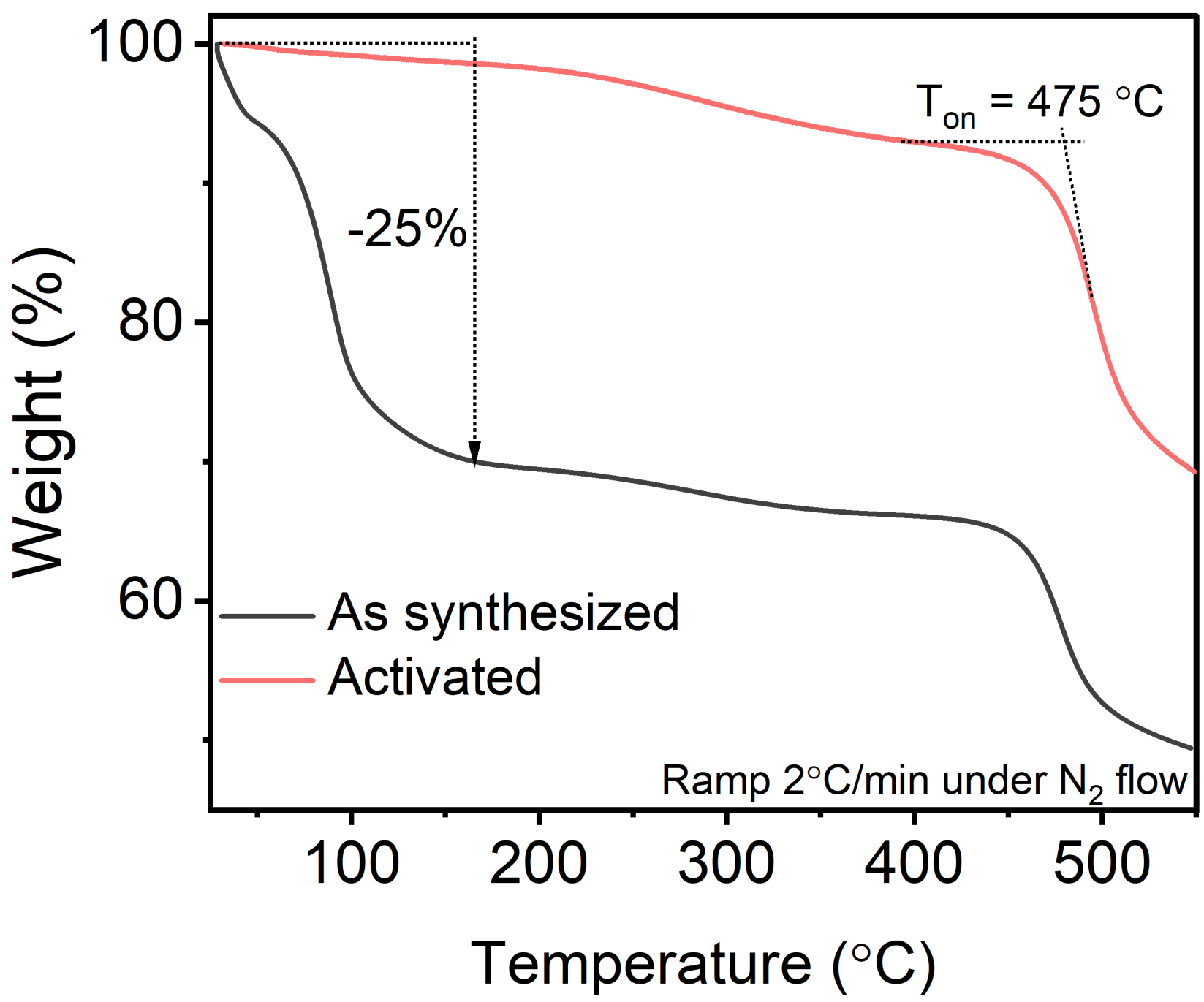


**Figure S7**: Thermograms of the as synthesized and activated (80 °C under dynamic vacuum for 8 hours) samples of **UoB-116($Dy_1La_0$)** under flowing $N_2$ (heating rate: 2 °C $min^{-1}$). The as-synthesized material undergoes an initial mass loss of approximately 25 weight% (wt%) below ≈160 °C, corresponding to the removal of occluded solvent molecules. By contrast, the activated sample exhibits negligible mass loss prior to framework decomposition, with an onset decomposition temperature ($T_{on}$) of 475 °C, confirming excellent thermal stability.

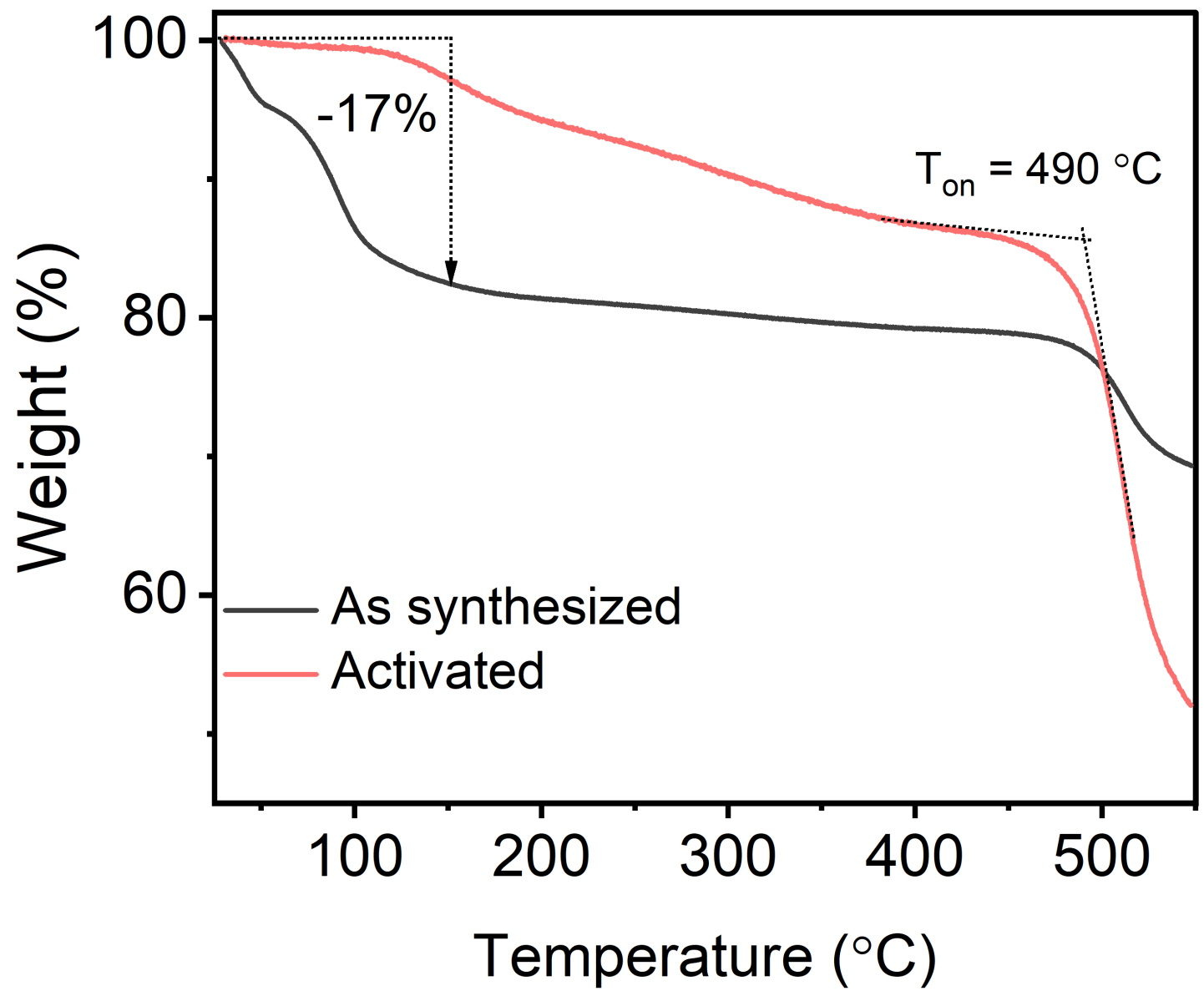


**Figure S8:** Thermograms of the as synthesized and activated (80 °C under dynamic vacuum for 8 hours) samples of **UoB-116($Dy_{0.82}La_{0.18}$)** under flowing $N_2$ (heating rate: 2 °C $min^{-1}$). The as-synthesized material undergoes an initial mass loss of approximately 17 weight% (wt%) below ≈160 °C, corresponding to the removal of occluded solvent molecules. By contrast, the activated sample exhibits negligible mass loss prior to framework decomposition, with an onset decomposition temperature ($T_{on}$) of 490 °C, confirming excellent thermal stability.

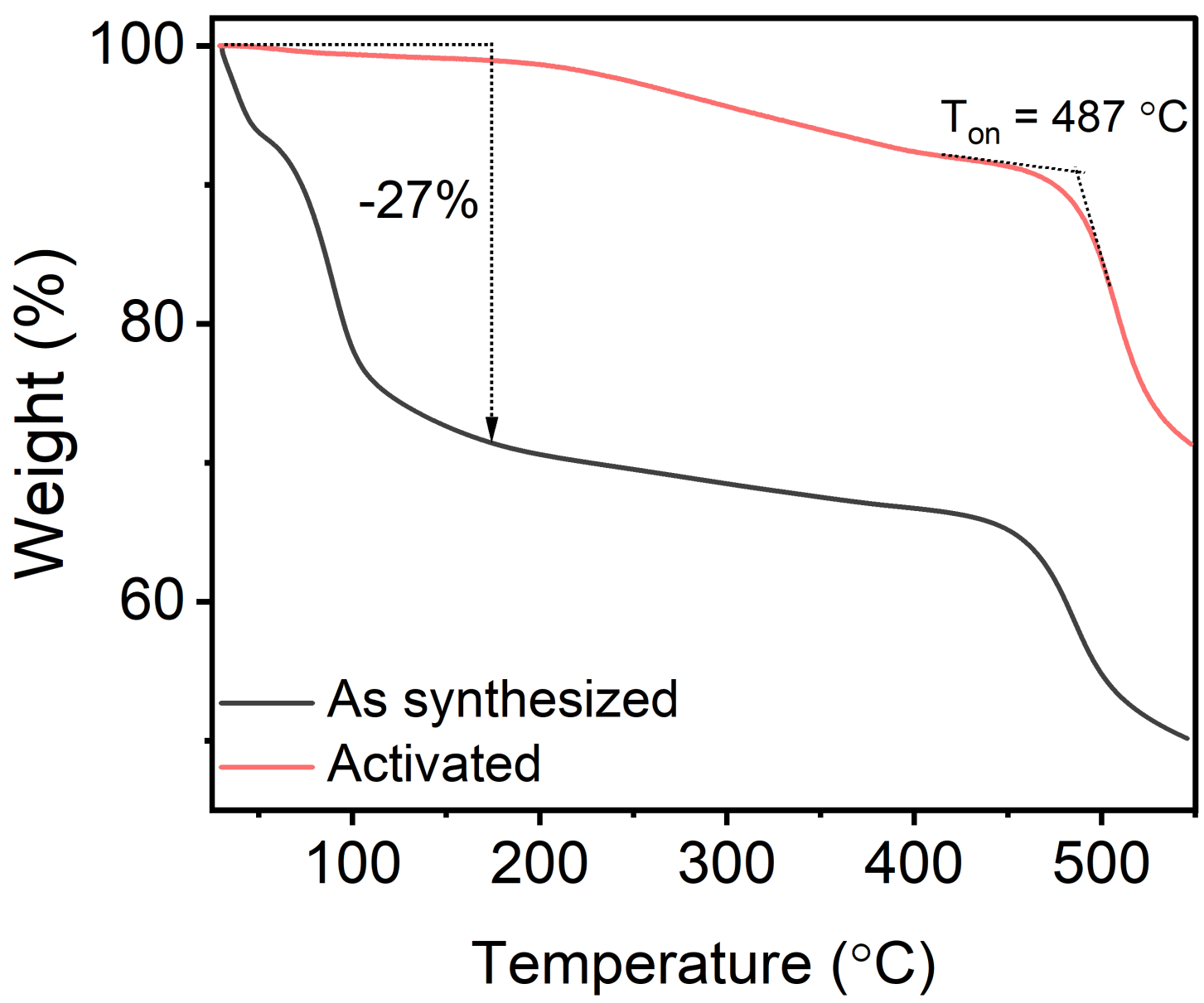


**Figure S9:** Thermograms of the as synthesized and activated (80 °C under dynamic vacuum for 8 hours) samples of **UoB-116($Dy_{0.64}La_{0.36}$)** under flowing $N_2$ (heating rate: 2 °C $min^{-1}$). The as-synthesized material undergoes an initial mass loss of approximately 27 weight% (wt%) below ≈170 °C, corresponding to the removal of occluded solvent molecules. In contrast, the activated sample exhibits negligible mass loss prior to framework decomposition, with an onset decomposition temperature ($T_{on}$) of 487 °C, confirming excellent thermal stability.

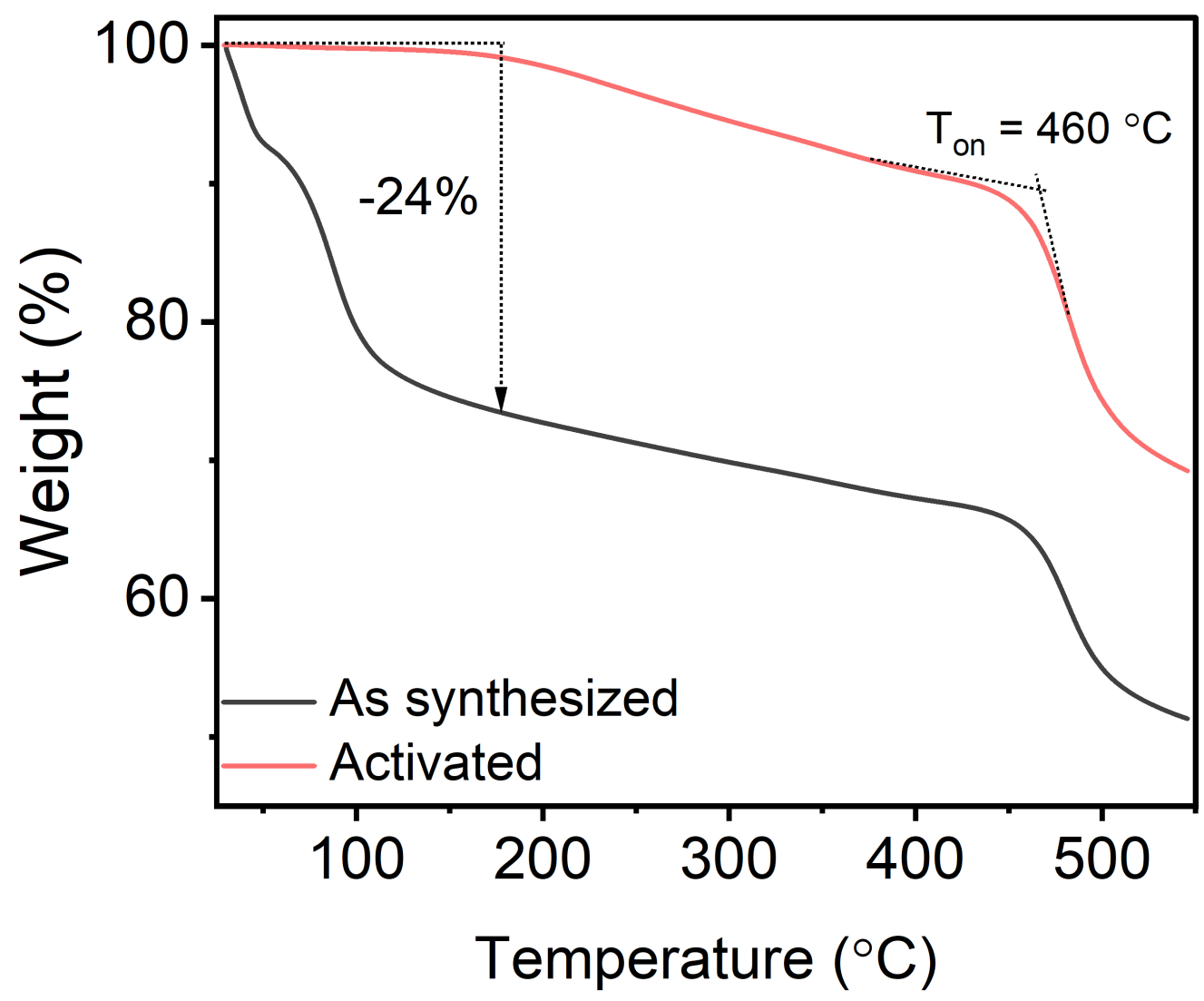


**Figure S10:** Thermograms of the as synthesized and activated (80 °C under dynamic vacuum for 8 hours) samples of **UoB-116($Dy_{0.36}La_{0.64}$)** under flowing $N_2$ (heating rate: 2 °C $min^{-1}$). The as-synthesized material undergoes an initial mass loss of approximately 24 weight% (wt%) below ≈180 °C, corresponding to the removal of occluded solvent molecules. In contrast, the activated sample exhibits negligible mass loss prior to framework decomposition, with an onset decomposition temperature (Ton) of 460 °C, confirming excellent thermal stability.

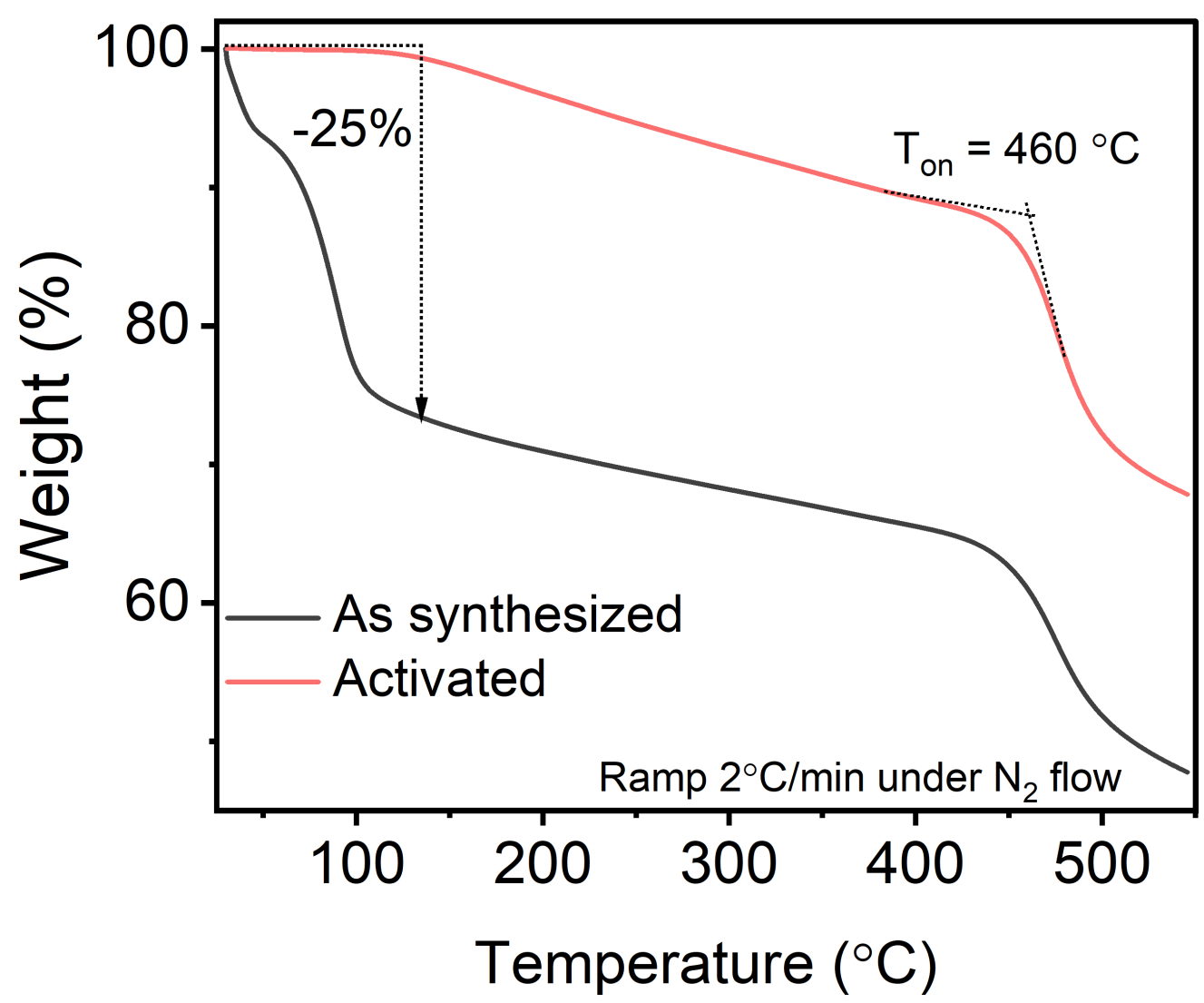


**Figure S11:** Thermograms of the as synthesized and activated (80 °C under dynamic vacuum for 8 hours) samples of **UoB-116($Dy_1La_0$)** under flowing $N_2$ (heating rate: 2 °C $min^{-1}$). The as-synthesized material undergoes an initial mass loss of approximately 25 weight% (wt%) below ≈130 °C, corresponding to the removal of occluded solvent molecules. In contrast, the activated sample exhibits negligible mass loss prior to framework decomposition, with an onset decomposition temperature (Ton) of 460 °C, confirming excellent thermal stability.

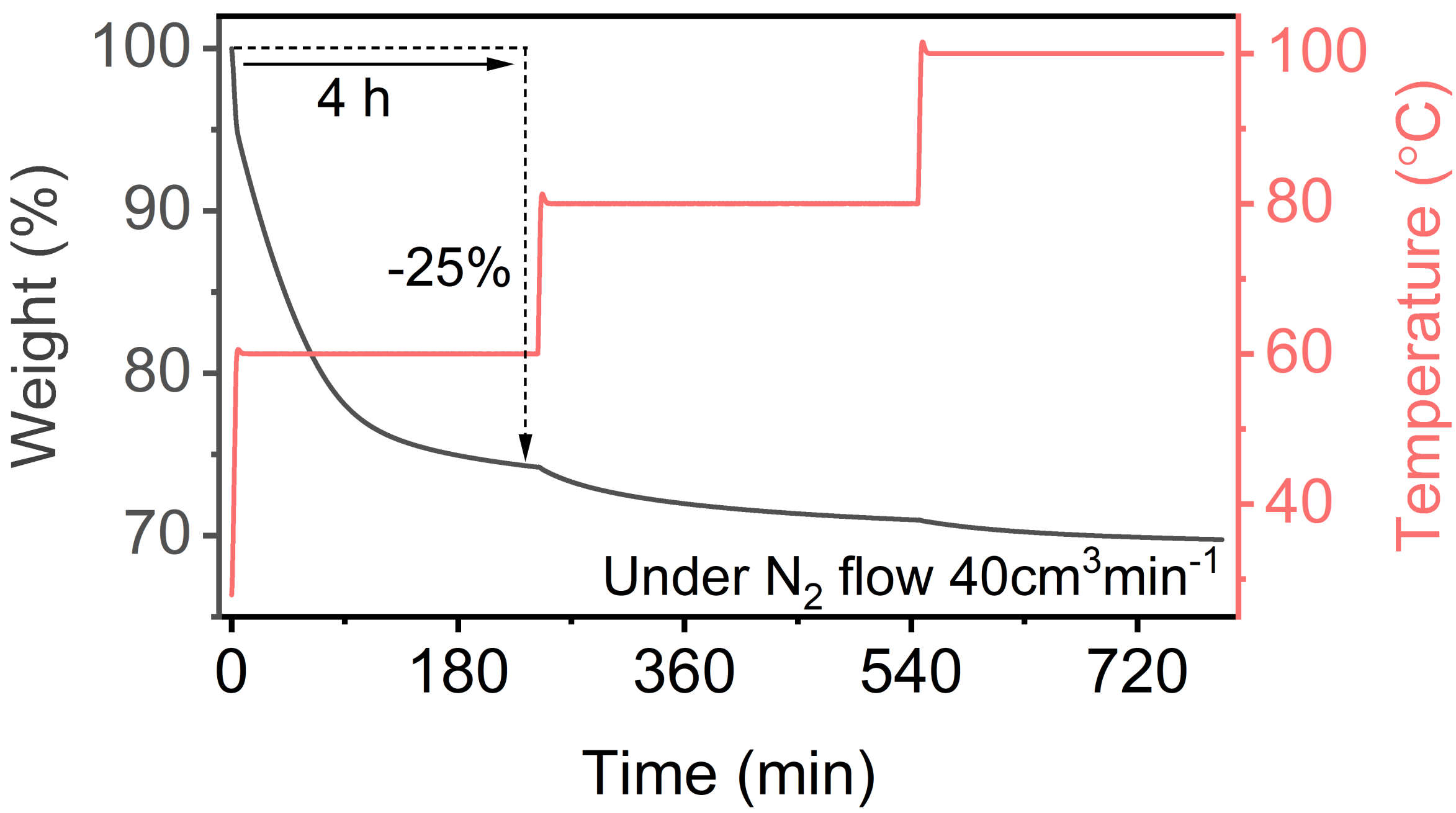


**Figure S12**: Isothermal TGA profile of the as synthesized sample of **UoB-116($Dy_1La_0$)** under $N_2$ flow (40 cm$^3$ min$^{-1}$). Approximately 25 wt% of guest molecules were found to be removed during a 4 h hold at 60 °C. Subsequent heating to 80 and 100 °C produced minimal additional mass loss, demonstrating that complete activation was achieved at 60 °C.

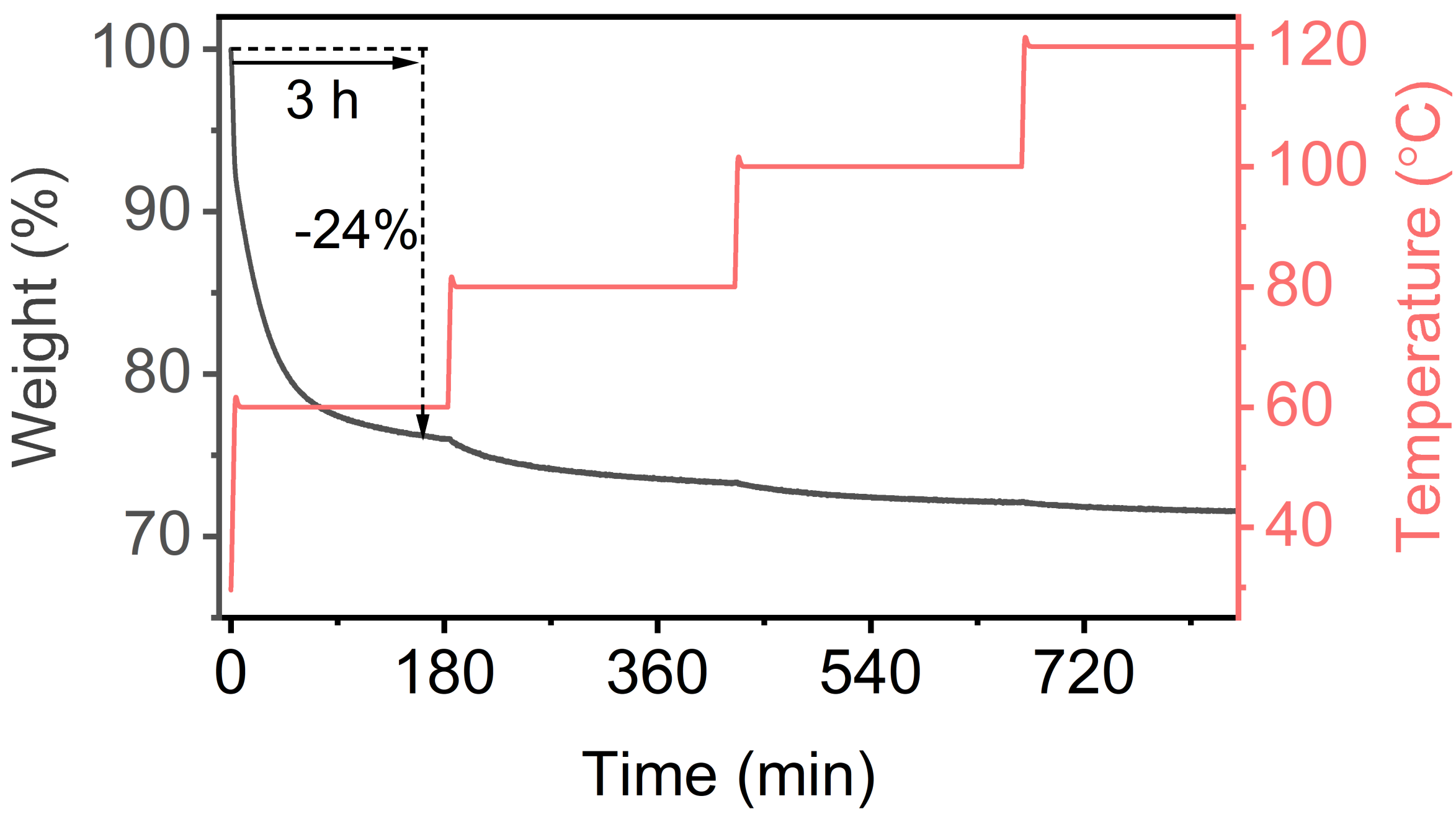


**Figure S13:** Isothermal TGA profile of the as synthesized sample of **UoB-116($Dy_{0.82}La_{0.18}$)** under $N_2$ flow (40 cm$^3$ min$^{-1}$). Approximately 25 wt% of guest molecules were found to be removed during a 4 h hold at 60 °C. Subsequent heating to 80 and 100 °C produced minimal additional mass loss, demonstrating that complete activation was achieved at 60 °C.

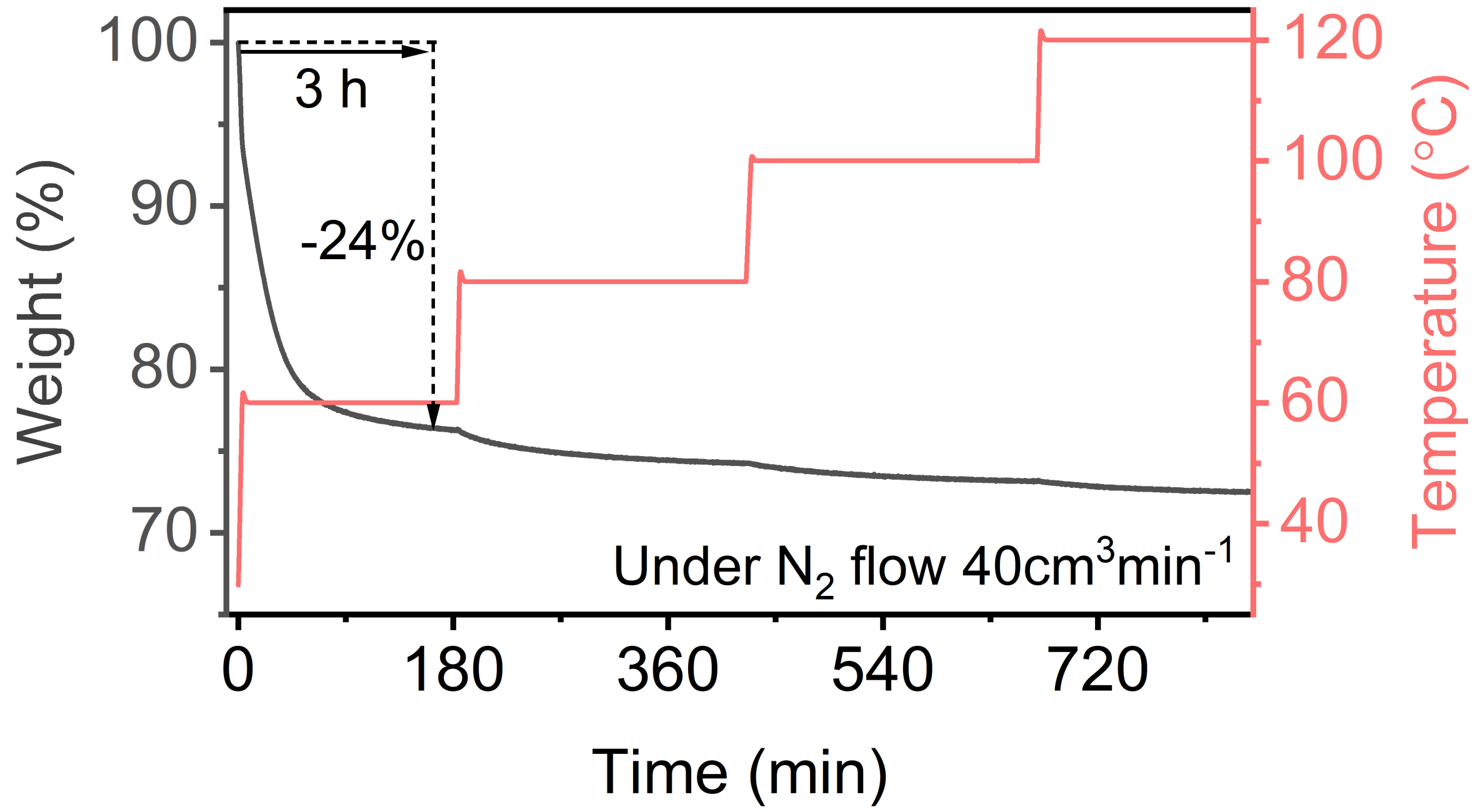


**Figure S14:** Isothermal TGA profile of the as synthesized sample of **UoB-116($Dy_{0.64}La_{0.36}$)** under $N_2$ flow (40 cm$^3$ min$^{-1}$). Approximately 25 wt% of guest molecules were found to be removed during a 4 h hold at 60 °C. Subsequent heating to 80 and 100 °C produced minimal additional mass loss, demonstrating that complete activation was achieved at 60 °C.

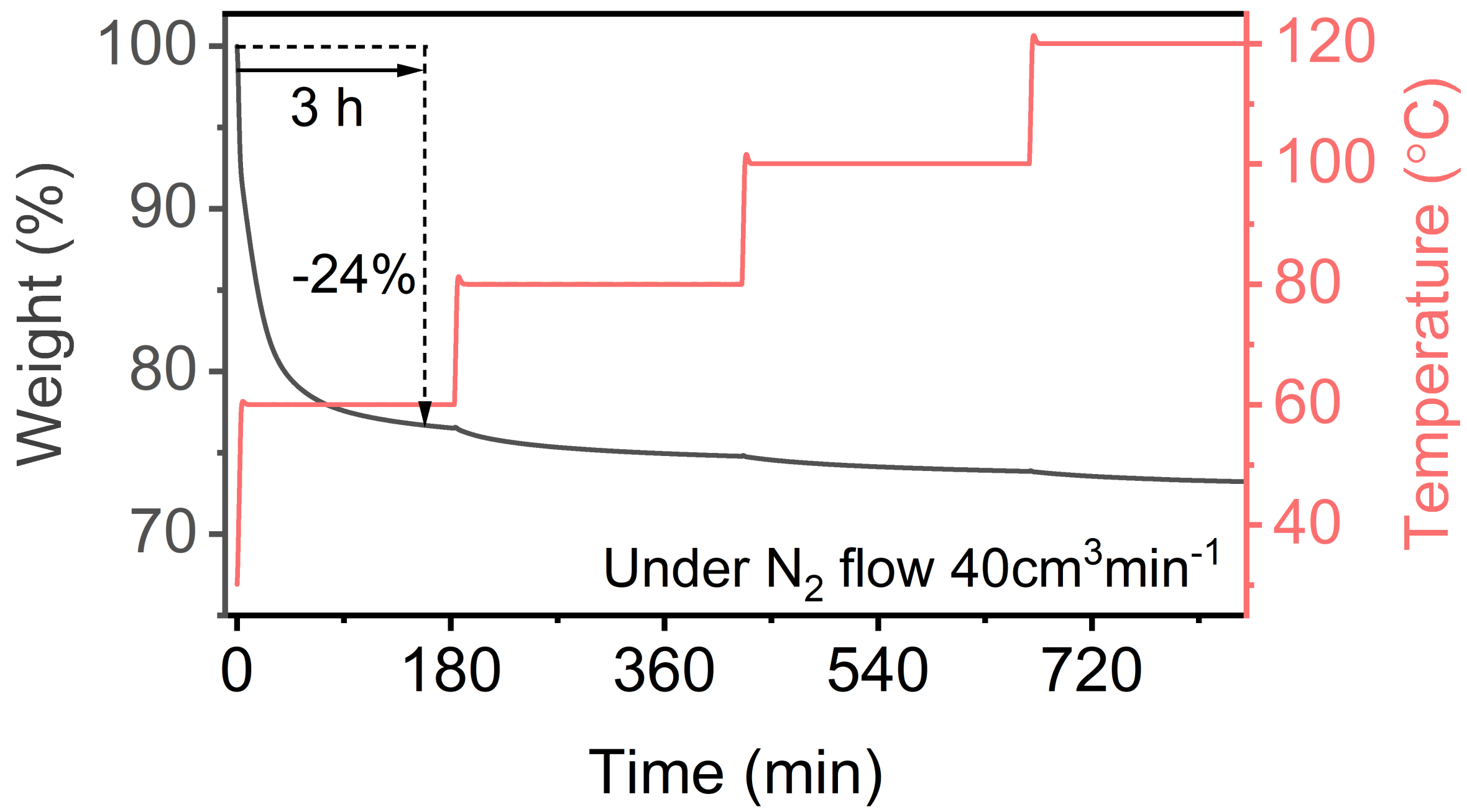


**Figure S15:** Isothermal TGA profile of the as synthesized sample of **UoB-116($Dy_{0.36}La_{0.64}$)** under $N_2$ flow (40 cm$^3$ min$^{-1}$). Approximately 25 wt% of guest molecules were found to be removed during a 4 h hold at 60 °C. Subsequent heating to 80 and 100 °C produced minimal additional mass loss, demonstrating that complete activation was achieved at 60 °C.

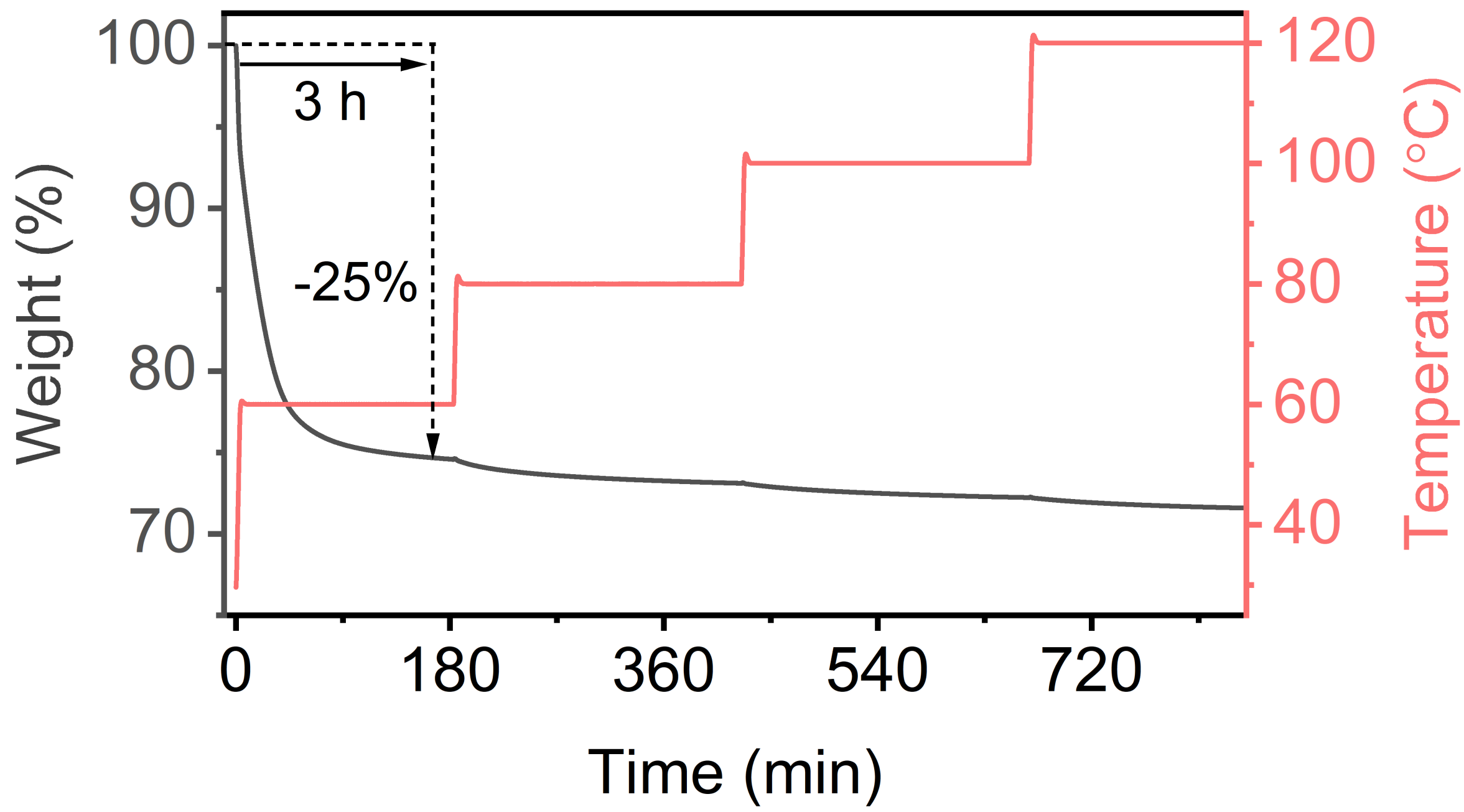


**Figure S16:** Isothermal TGA profile of the as synthesized sample of **UoB-116($Dy_1La_0$)** under $N_2$ flow (40 $cm^3$ $min^{-1}$). Approximately 25 wt% of guest molecules were found to be removed during a 4 h hold at 60 °C. Subsequent heating to 80 and 100 °C produced minimal additional mass loss, demonstrating that complete activation was achieved at 60 °C.

**Variable-temperature Powder X-ray diffraction (VT-PXRD)**

Powder X-ray diffractograms (PXRD) at different temperatures were recorded *in situ*, using an Anton Paar XRDynamic 500: TTK 600 diffractometer equipped with a PIXcel3D detector operating in scanning line detector mode. Anton Paar TTK 450 stage coupled with the Anton Paar TCU 110 Temperature Control Unit was used to record the variable-temperature diffractograms. Continuous scanning mode with the goniometer in the theta-theta orientation was used to collect the data. Incident beam optics included the Fixed Divergences slit, with a 1/4° divergence slit and a Soller slit (0.04 rad). Divergent beam optics included a P7.5 S7 anti-scatter slit, a Soller slit (0.04 rad), and a Ni-β filter. In a typical experiment, ~20 mg of the sample was loaded on a zero-background disc made for Anton Paar TTK 600 chamber. The data was recorded between 4° and 40° (2θ) with a step-size of 0.0167113°, and a scan time of 200 seconds per step. Crude PXRD patterns were analysed using the X'Pert HighScore Plus9 ™ software V 4.1 (PANalytical, The Netherlands).

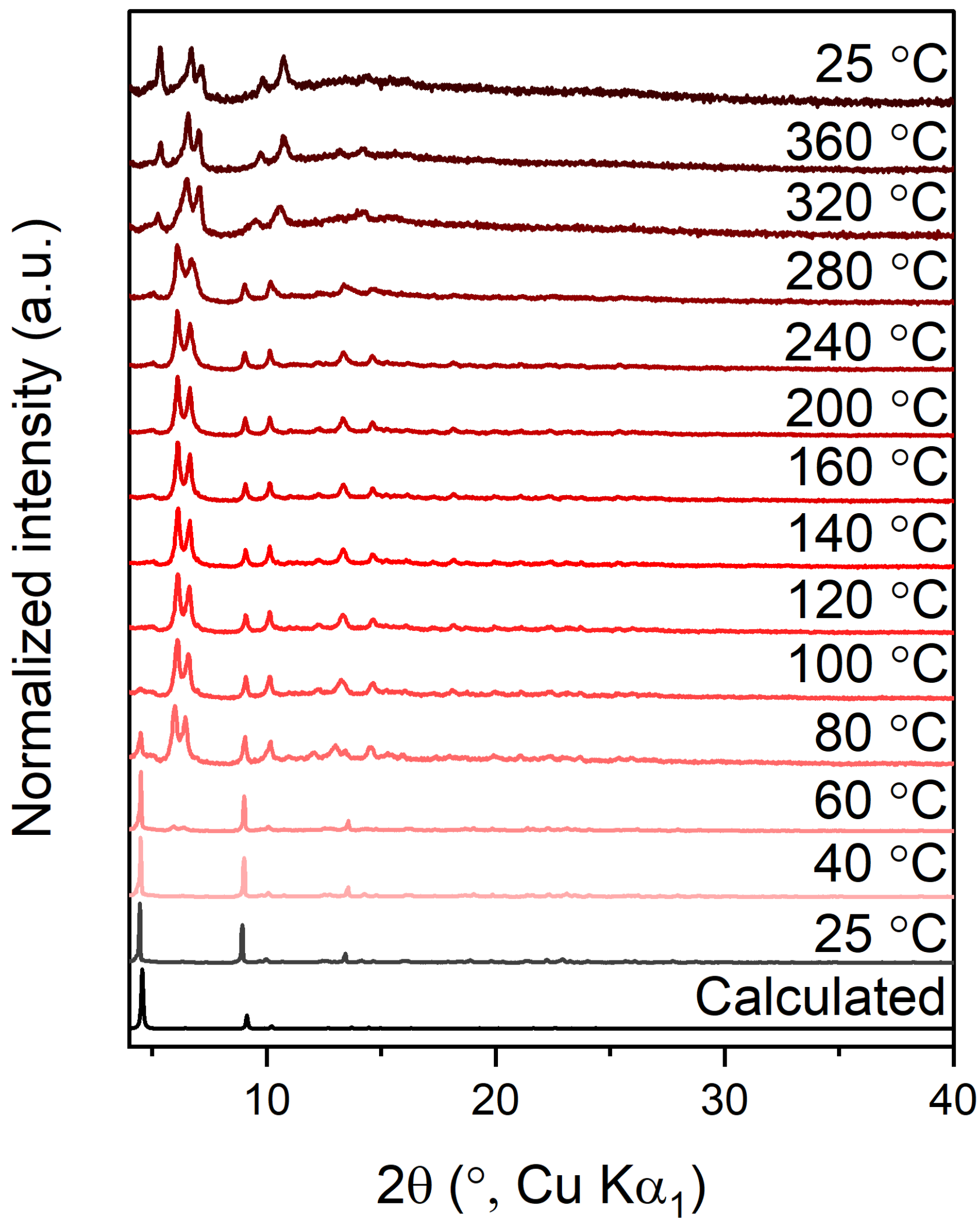


**Figure S17:** Variable-temperature PXRD patterns of as-synthesized **UoB-116($Dy_1La_0$)**, recorded under $N_2$ flow.

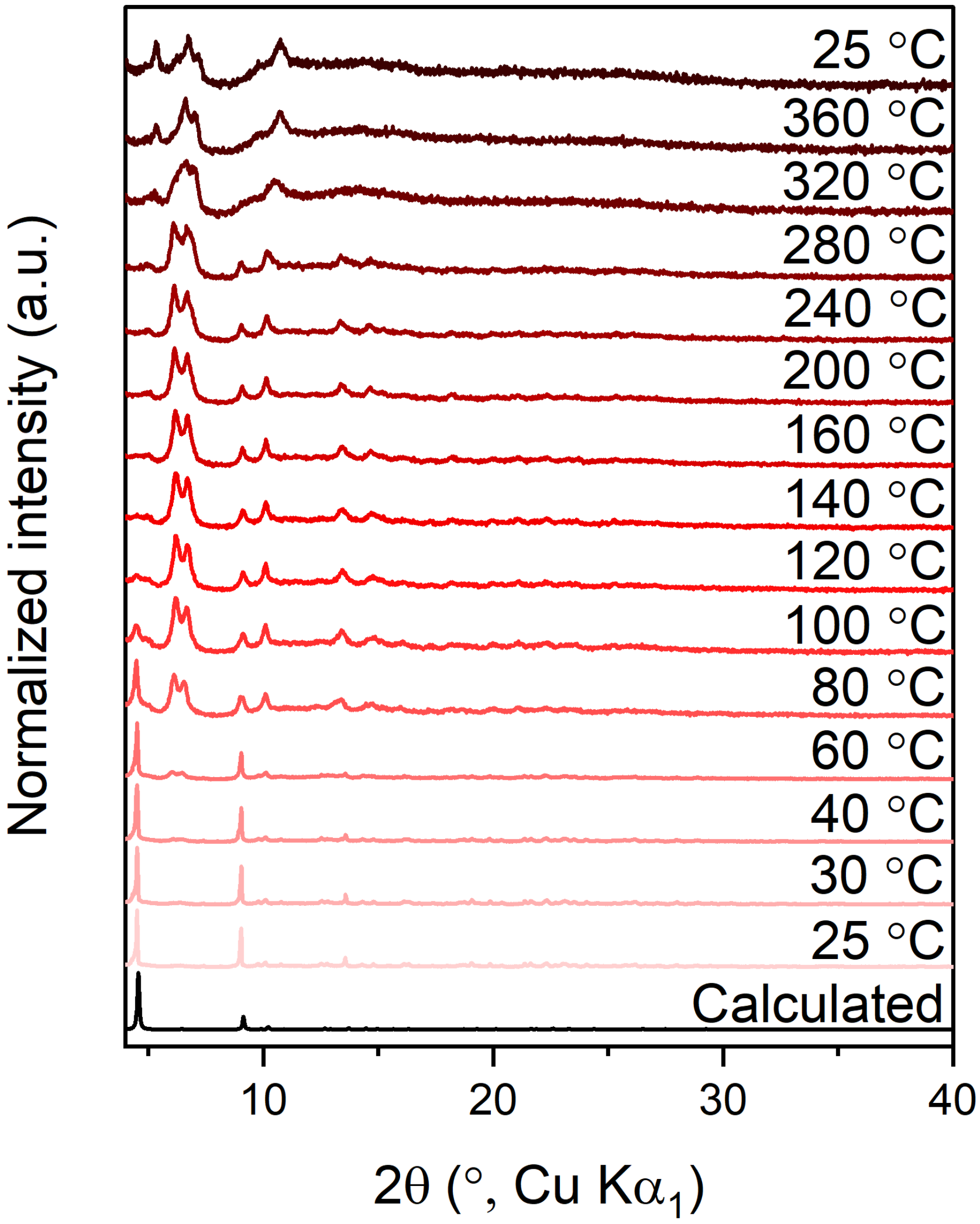


**Figure S18:** Variable-temperature PXRD patterns of as-synthesized **UoB-116($Dy_{0.82}La_{0.18}$)** recorded under $N_2$ flow.

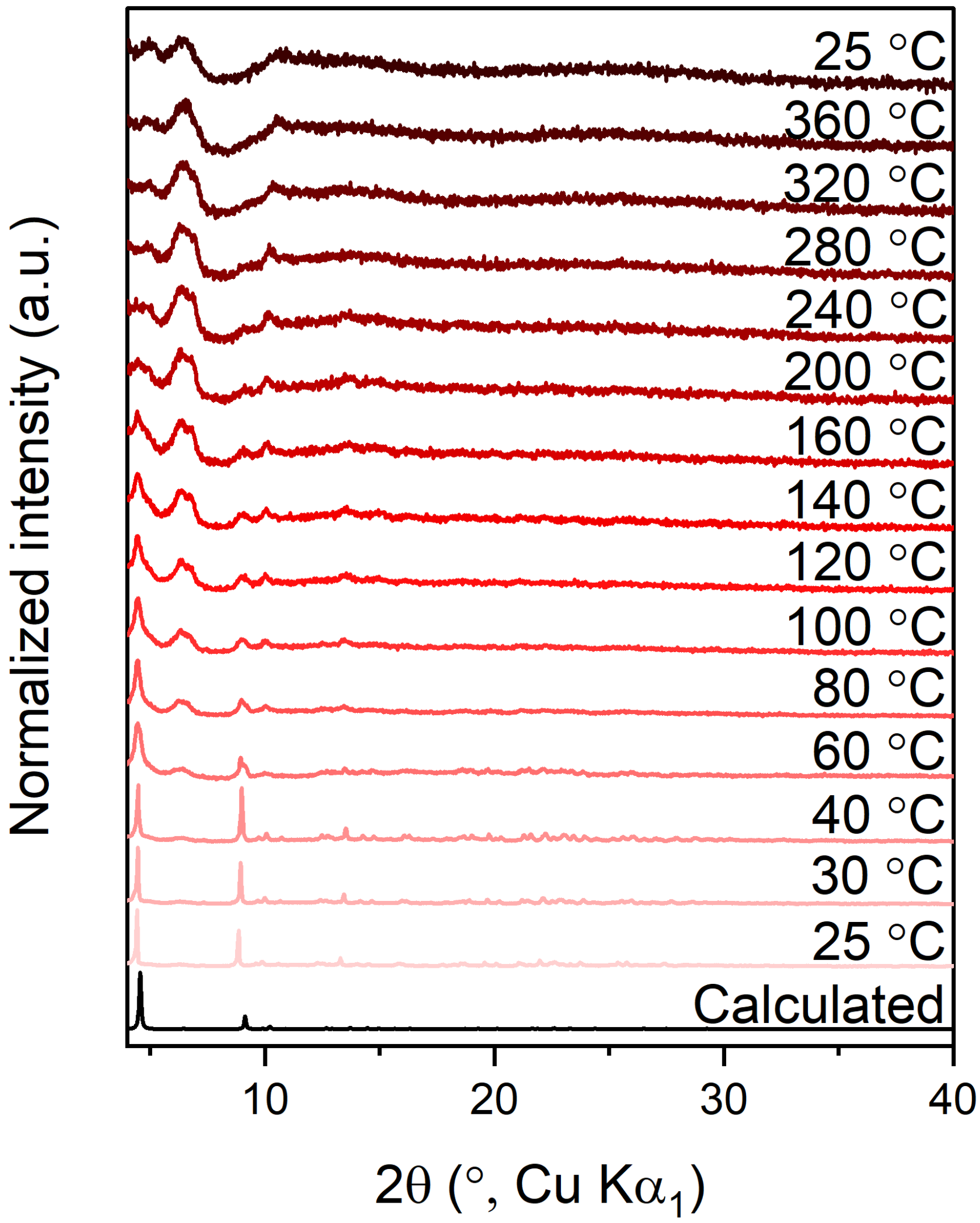


**Figure S19:** Variable-temperature PXRD patterns of as-synthesized **UoB-116($Dy_{0.64}La_{0.36}$)** recorded under $N_2$ flow.

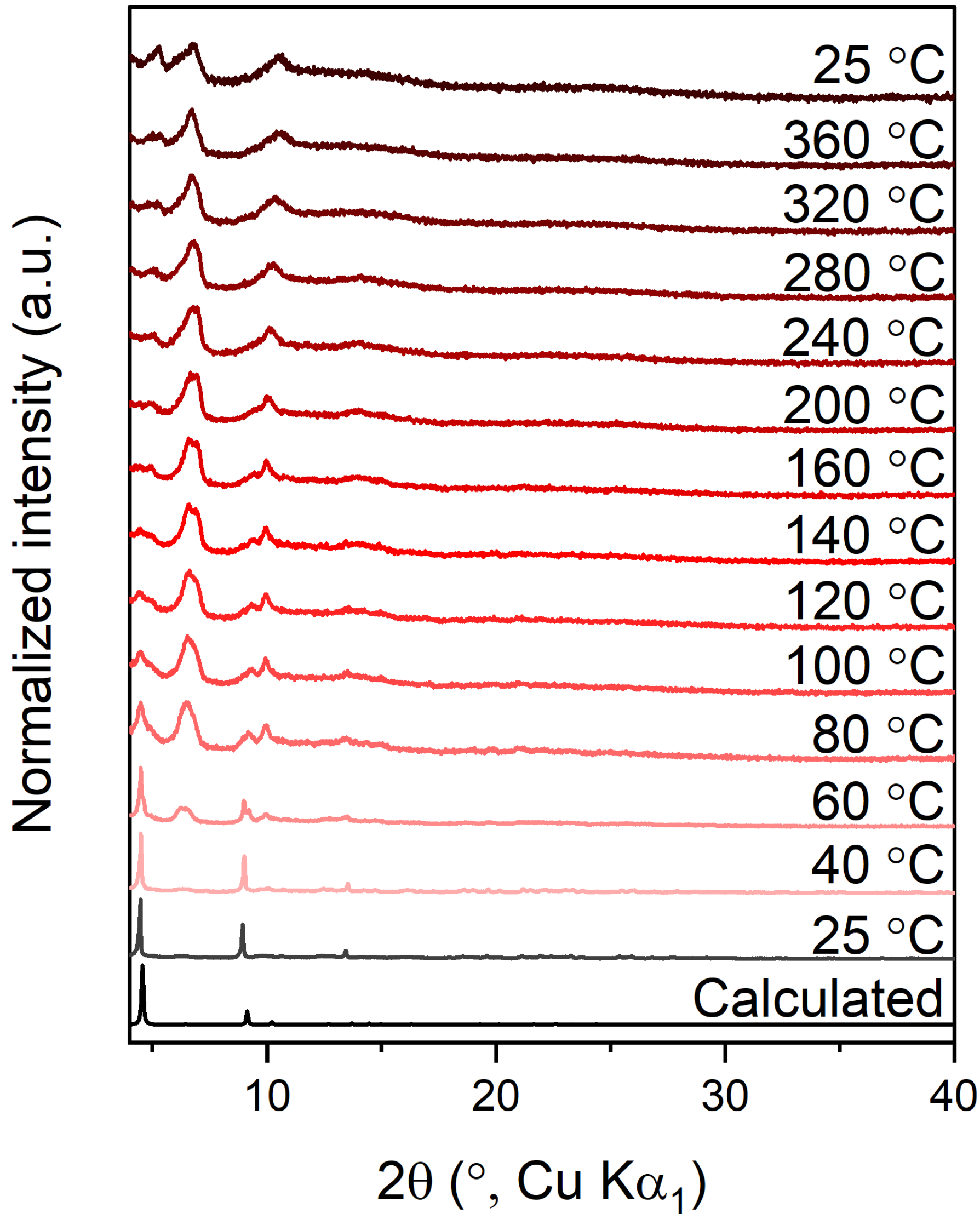


**Figure S20**: Variable-temperature PXRD patterns of as-synthesized **UoB-116($Dy_{0.36}La_{0.64}$)** recorded under $N_2$ flow.

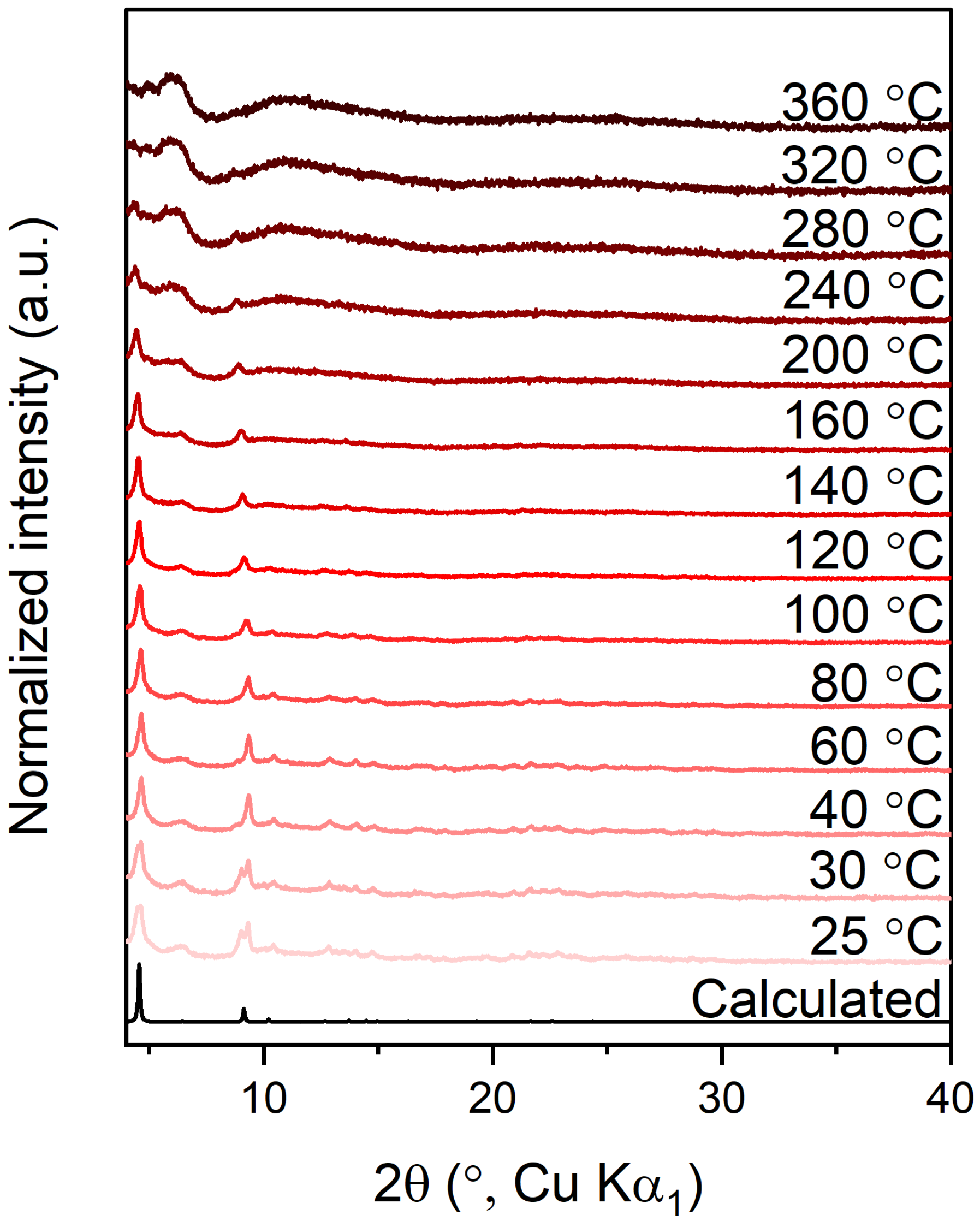


**Figure S21**: Variable-temperature PXRD patterns of as-synthesized **UoB-116(Dy$_0$La$_1$)** recorded under $N_2$ flow.

**Single-component gas sorption experiments**

The samples were activated under high vacuum by a Micromeritics® Smart VacPrep$^{TM}$ at 60 °C for 8 h before gas sorption studies. The $CO_2$ sorption isotherms at 195 K were measured by a Micromeritics® 3Flex adsorption analyser. The 195 K environment was controlled by a 2.75 L dewar vessel containing a bath of acetone and dry ice. 273 K, 283 K and 298 K environment was controlled by a Julabo® temperature controller with the ethylene glycol/water = 1:1 (*v/v*) mixture liquid. Surface areas were calculated from the adsorption data using BET method. The pore-size distribution curves were obtained from the adsorption branches using Horváth–Kawazoe (H-K) method.

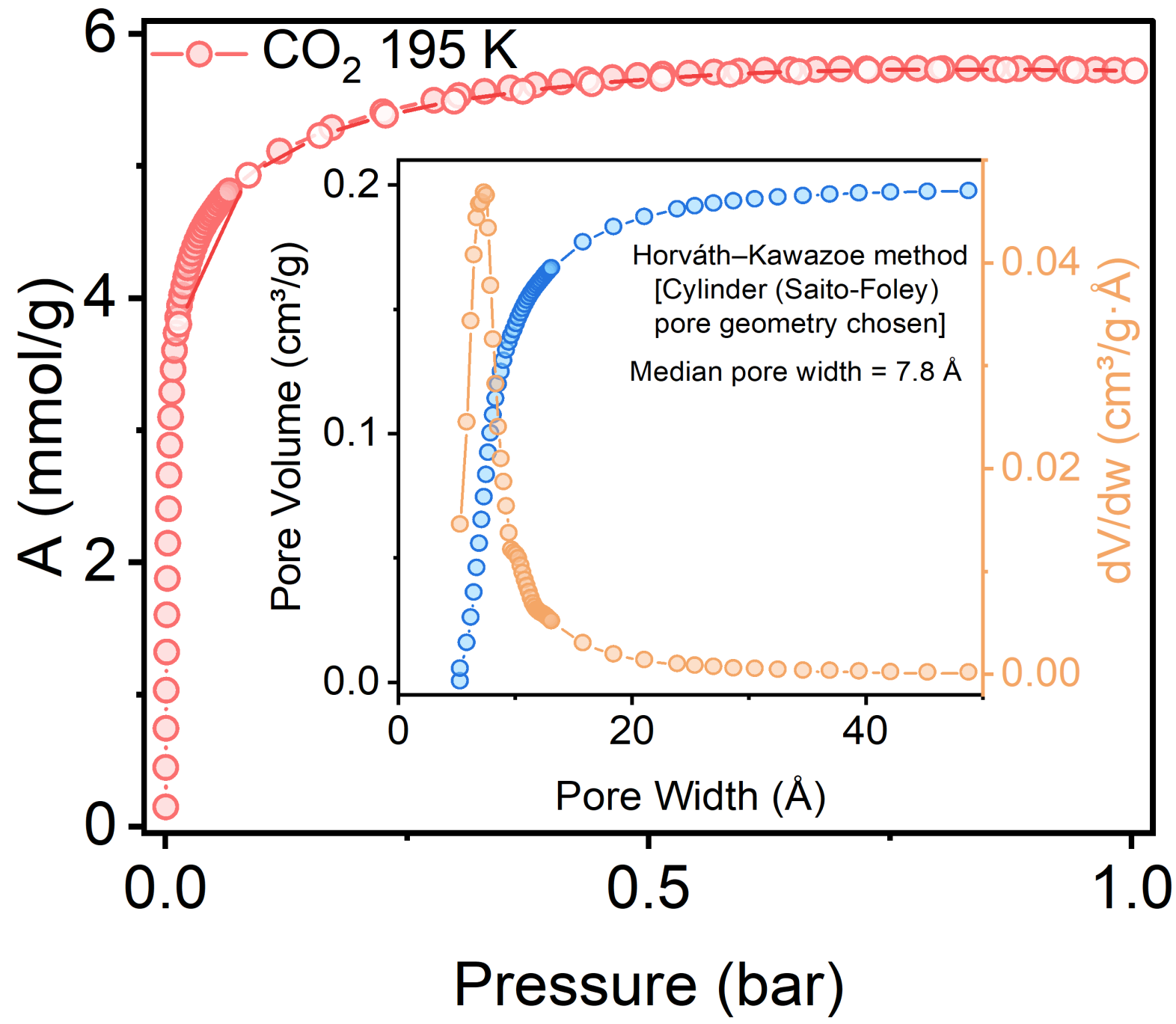


**Figure S22:** $CO_2$ adsorption isotherm at 195 K for activated **UoB-116($Dy_1La_0$)**. **Inset:** Cumulative pore volume (blue) and differential pore size distribution (orange) obtained using the Horváth–Kawazoe (Saito–Foley cylindrical pore) model, yielding a median pore width of 7.8 Å.

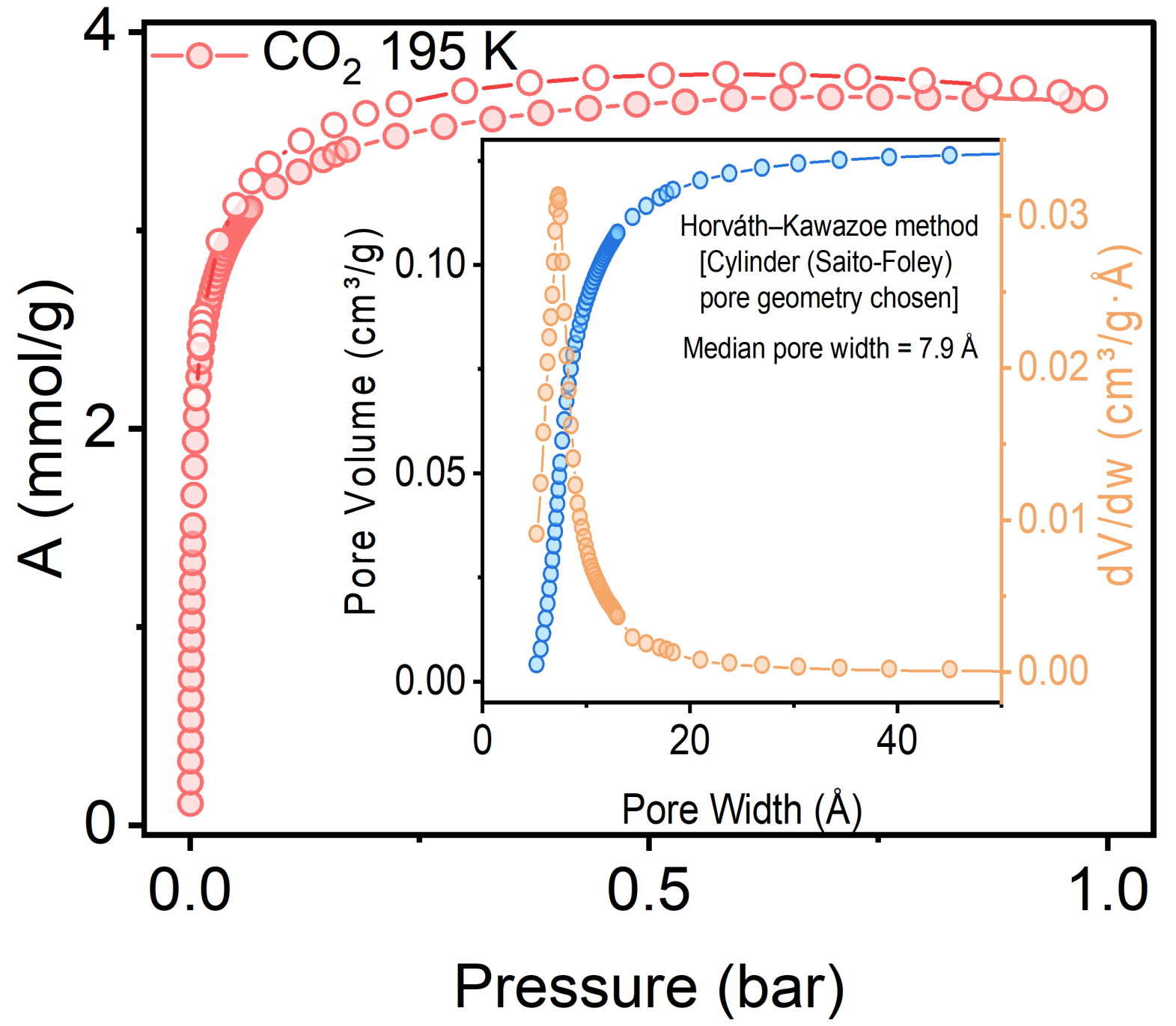


**Figure S23:** $CO_2$ adsorption isotherm at 195 K for activated **UoB-116($Dy_{0.82}La_{0.18}$)**. Inset: Cumulative pore volume (blue) and differential pore size distribution (orange) obtained using the Horváth–Kawazoe (Saito–Foley cylindrical pore) model, yielding a median pore width of 7.9 Å.

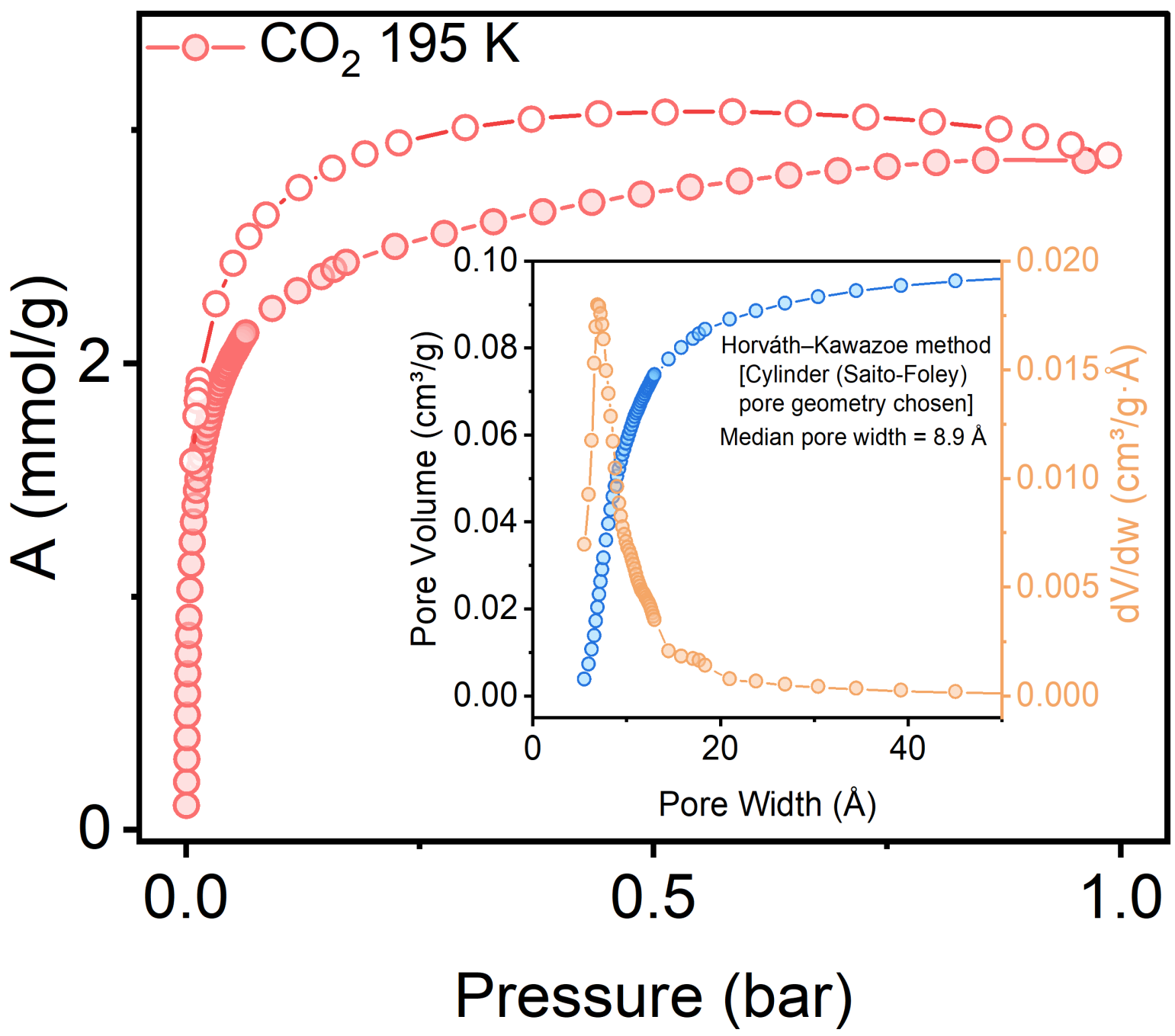


**Figure S24:** $CO_2$ adsorption isotherm at 195 K for activated **UoB-116($Dy_{0.64}La_{0.36}$)**. Inset: Cumulative pore volume (blue) and differential pore size distribution (orange) obtained using the Horváth–Kawazoe (Saito–Foley cylindrical pore) model, yielding a median pore width of 8.9 Å.

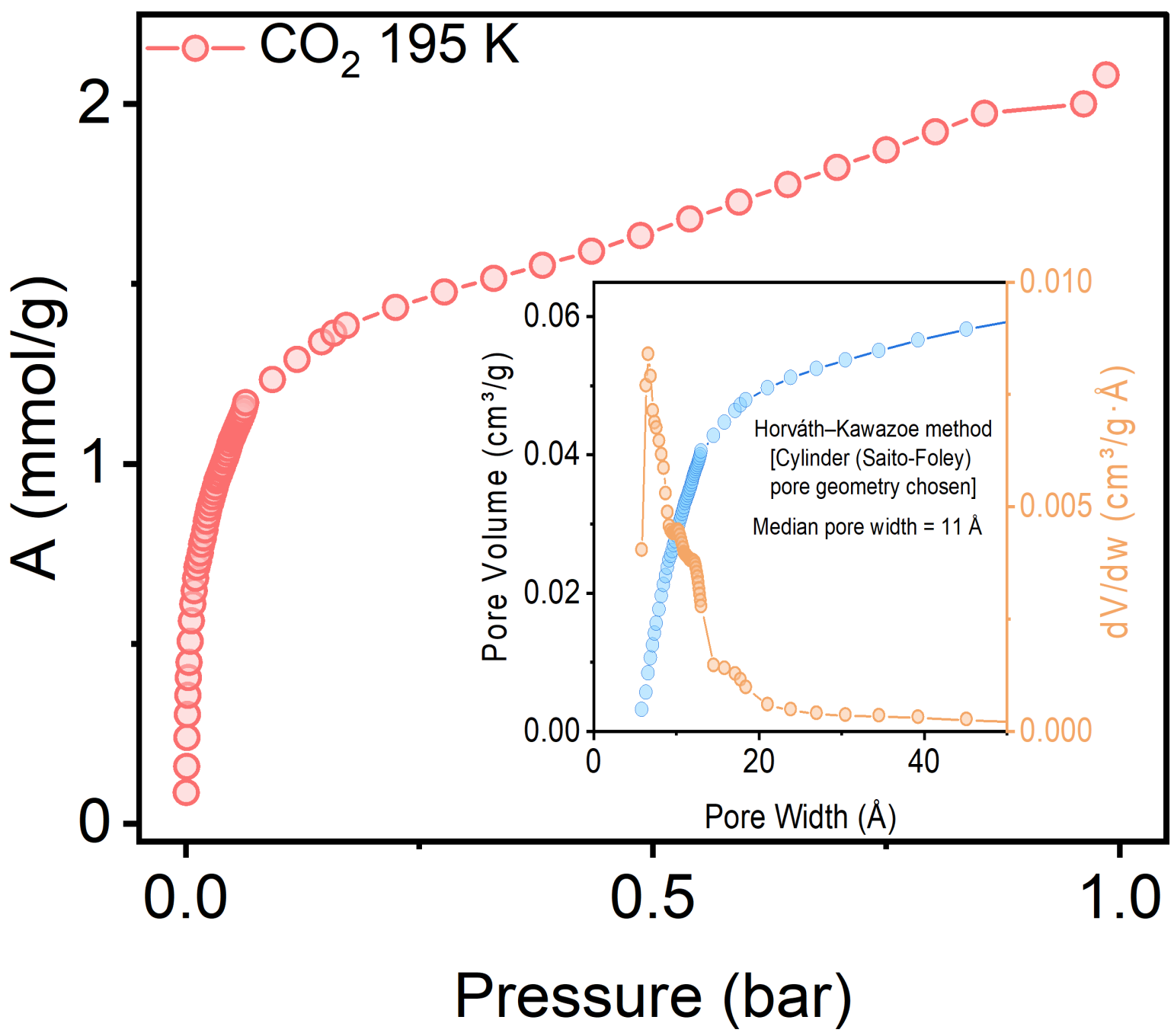


**Figure S25:** $CO_2$ adsorption isotherm at 195 K for activated **UoB-116($Dy_{0.36}La_{0.64}$)**. Inset: Cumulative pore volume (blue) and differential pore size distribution (orange) obtained using the Horváth–Kawazoe (Saito–Foley cylindrical pore) model, yielding a median pore width of 11 Å.

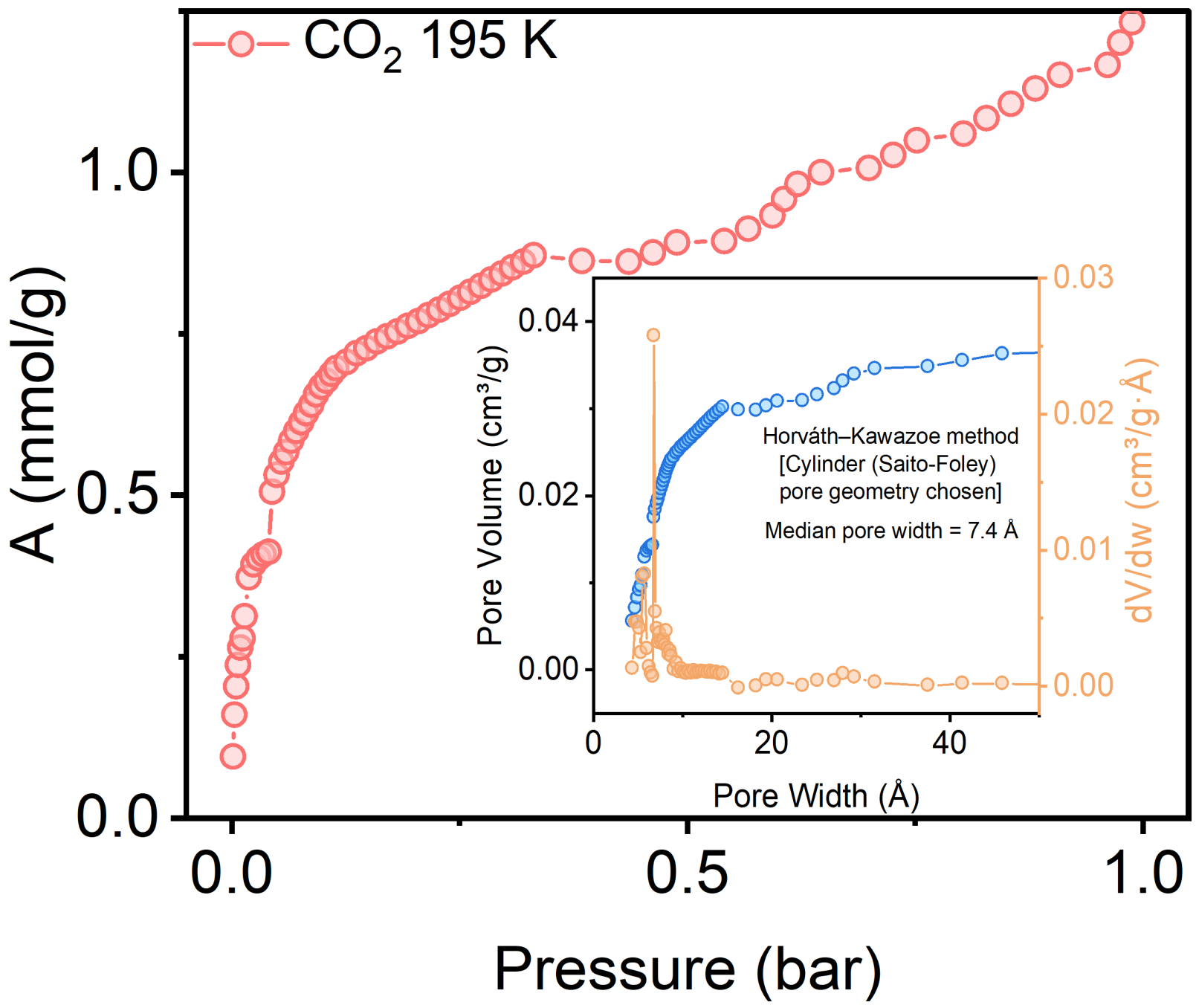


**Figure S26:** $CO_2$ adsorption isotherm at 195 K for activated **UoB-116($Dy_0La_1$)**. The relatively low $CO_2$ uptake suggests limited microporosity. Inset: Cumulative pore volume (blue) and differential pore size distribution (orange) obtained using the Horváth–Kawazoe (Saito–Foley cylindrical pore) model, yielding a median pore width of ~7.4 Å. Owing to the low pore volume and weak adsorption isotherm , the derived pore size distribution should be interpreted with caution.

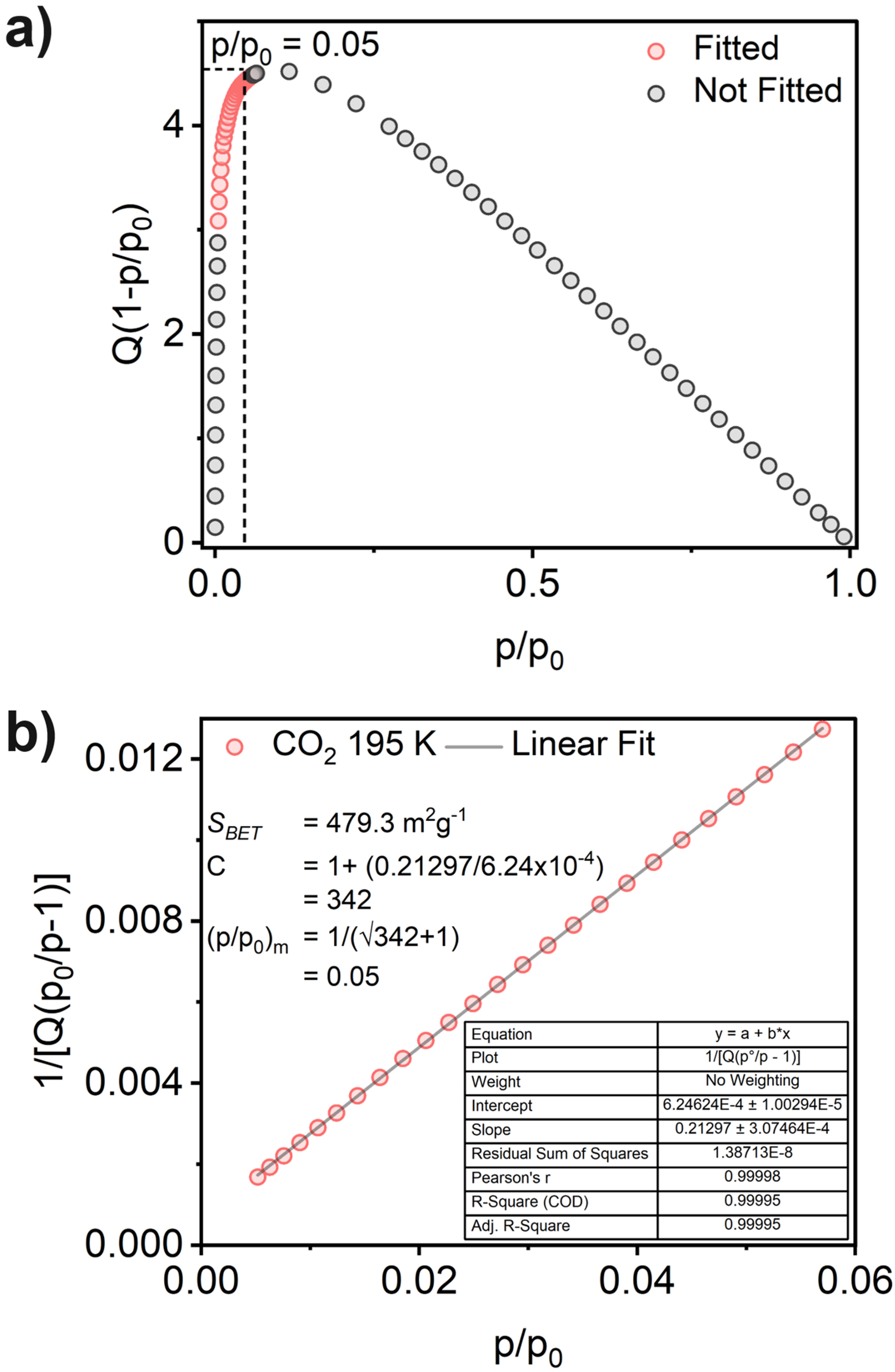


**Figure S27**: (**a)** Rouquerol consistency plot used to identify the appropriate relative pressure range for Brunauer–Emmett–Teller (BET) surface area analysis from the $CO_2$ adsorption isotherm at 195 K of **UoB-116(Dy$_1$La$_0$)**. Data within the valid fitting region ($p/p_0 \leq 0.05$) are highlighted in red, while excluded points are shown in grey. **(b)** Linear BET plot constructed from the selected pressure range, yielding a BET surface area of 479.3 m$^2$ g$^{-1}$ with a BET constant (**C**) of 342, indicating an excellent linear fit ($R^2$ = 0.99995).

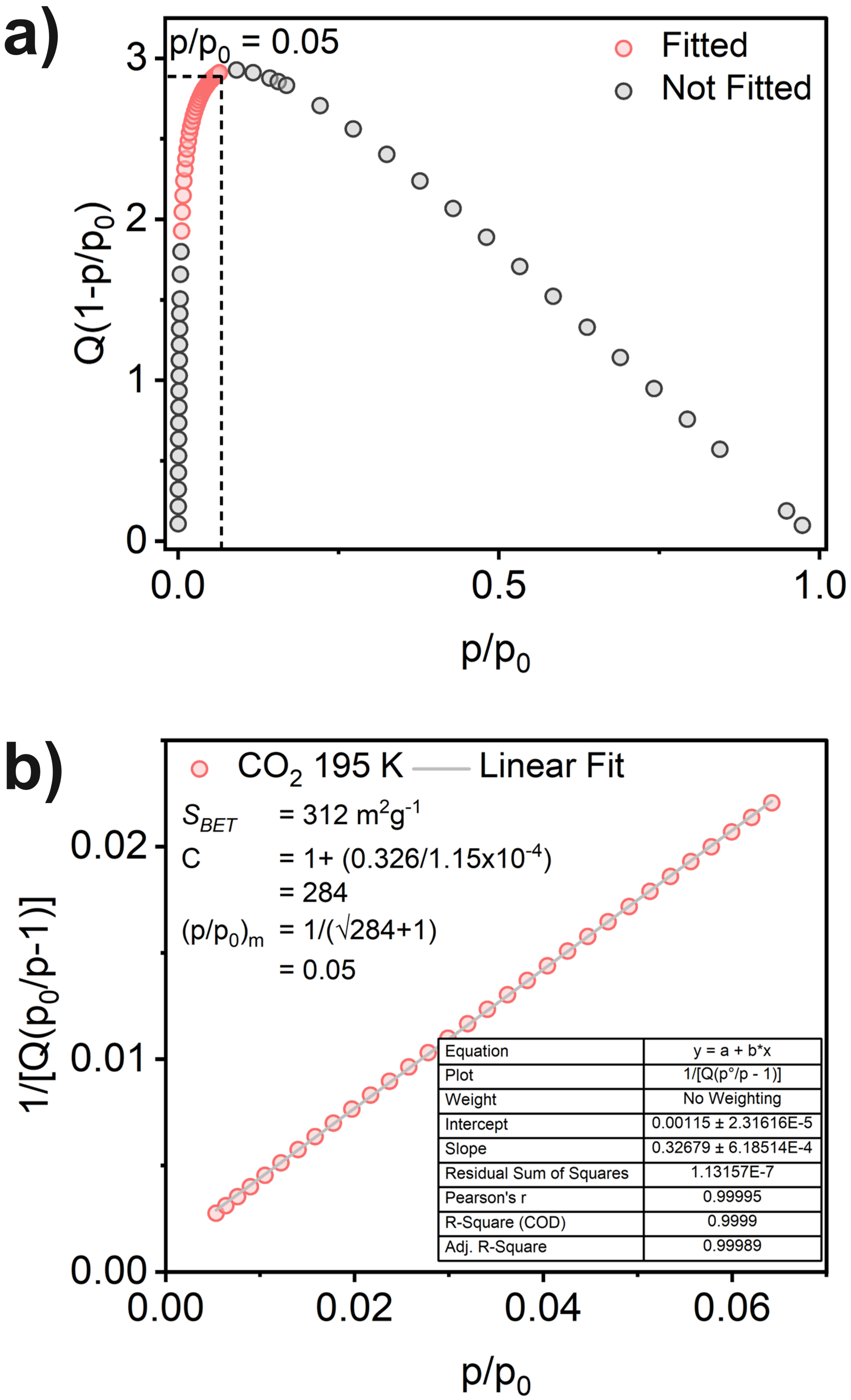


**Figure S28**: **(a)** Rouquerol consistency plot used to identify the appropriate relative pressure range for Brunauer–Emmett–Teller (BET) surface area analysis from the $CO_2$ adsorption isotherm at 195 K of **UoB-116($Dy_{0.82}La_{0.18}$)**. Data within the valid fitting region ($p/p_0 \leq 0.05$) are highlighted in red, while excluded points are shown in grey. **(b)** Linear BET plot constructed from the selected pressure range, yielding a BET surface area of 312 $m^2\ g^{-1}$ with a BET constant (**C**) of 284, indicating an excellent linear fit ($R^2$ = 0.99989).

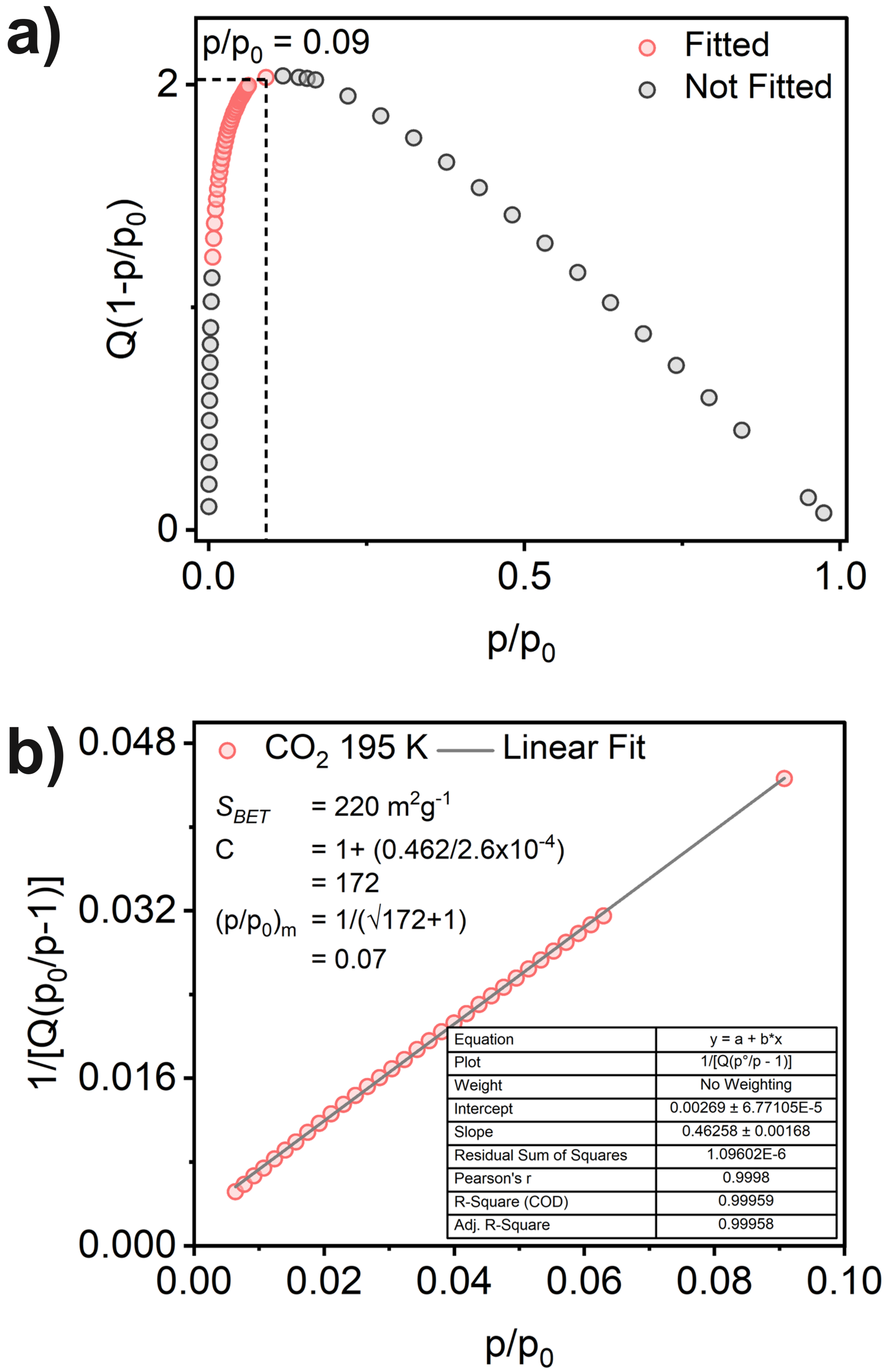


**Figure S29**: (**a)** Rouquerol consistency plot used to identify the appropriate relative pressure range for Brunauer–Emmett–Teller (BET) surface area analysis from the $CO_2$ adsorption isotherm at 195 K of **UoB-116($Dy_{0.64}La_{0.36}$)**. Data within the valid fitting region ($p/p_0 \leq 0.09$) are highlighted in red, while excluded points are shown in grey. **(b)** Linear BET plot constructed from the selected pressure range, yielding a BET surface area of 220 $m^2\ g^{-1}$ with a BET constant (**C**) of 172, indicating an excellent linear fit ($R^2$ = 0.99958).

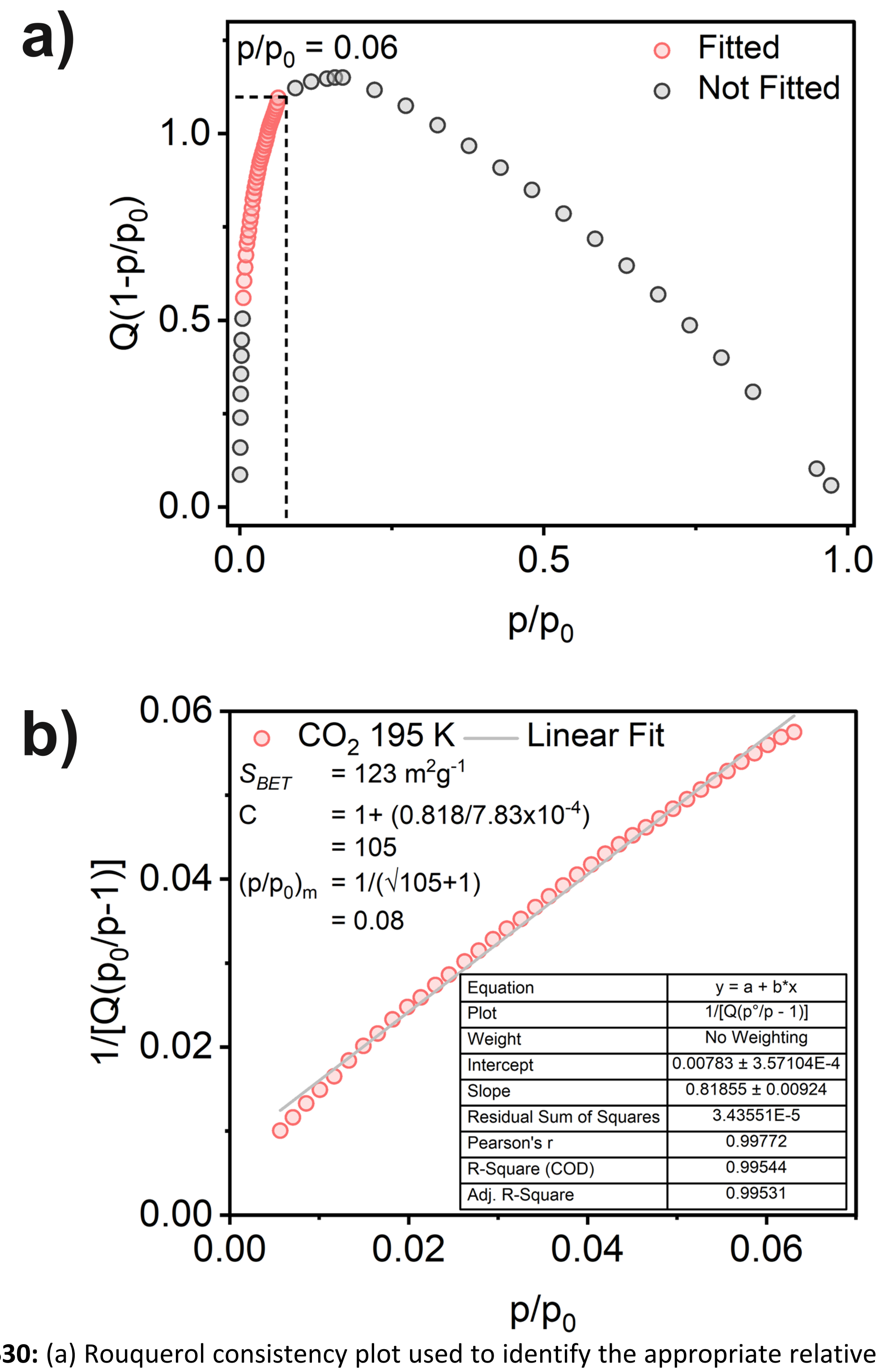


**Figure S30:** (a) Rouquerol consistency plot used to identify the appropriate relative pressure range for Brunauer–Emmett–Teller (BET) surface area analysis from the $CO_2$ adsorption isotherm at 195 K of **UoB-116($Dy_{0.36}La_{0.64}$)**. Data within the valid fitting region ($p/p_0 \leq 0.06$) are highlighted in red, while excluded points are shown in grey. (b) Linear BET plot constructed from the selected pressure range, yielding a BET surface area of 123 $m^2\ g^{-1}$ with a BET constant (C) of 105, indicating an excellent linear fit ($R^2 = 0.99531$).

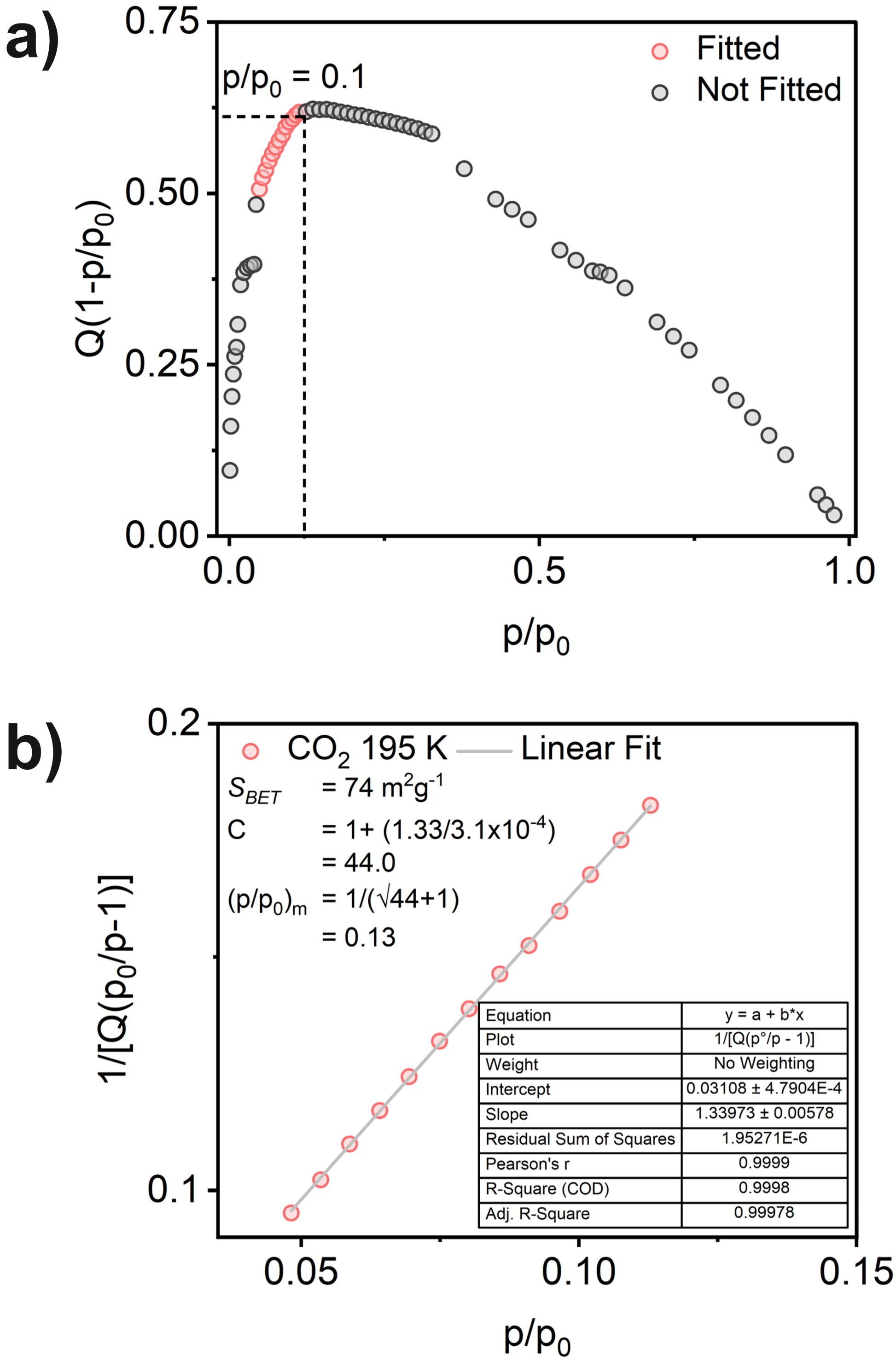


**Figure S31:** (a) Rouquerol consistency plot used to identify the appropriate relative pressure range for Brunauer–Emmett–Teller (BET) surface area analysis from the $CO_2$ adsorption isotherm at 195 K of **UoB-116(Dy$_0$La$_1$)**. Data within the valid fitting region ($p/p_0 \leq 0.1$) are highlighted in red, while excluded points are shown in grey. (b) Linear BET plot constructed from the selected pressure range, yielding a BET surface area of 74 $m^2\ g^{-1}$ with a BET constant (C) of 44, indicating an excellent linear fit ($R^2 = 0.99978$). Although the selected fitting region satisfies the Rouquerol consistency criteria, the relatively low porosity of the material warrants cautious interpretation of the derived BET surface area.

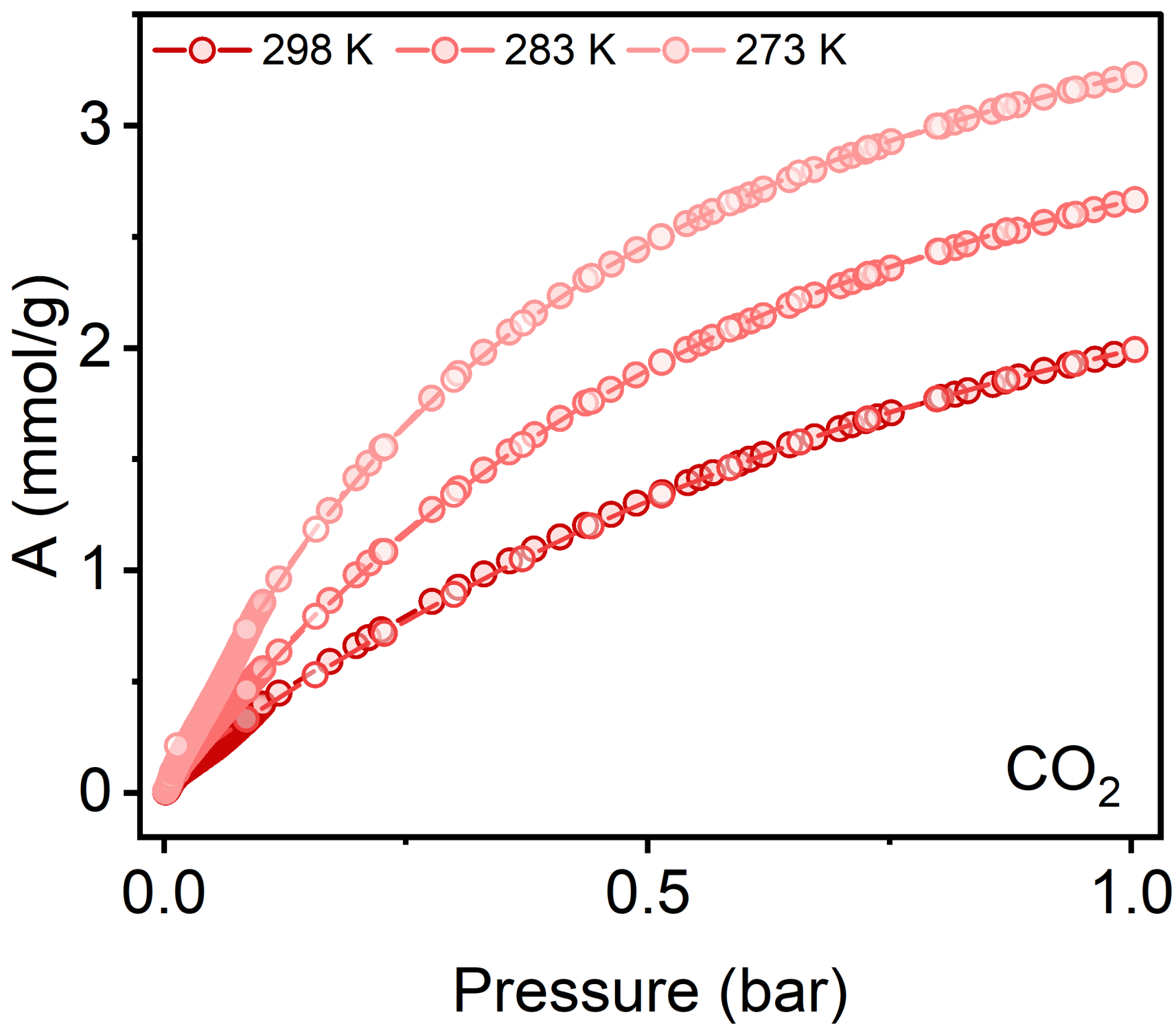


**Figure S32:** $CO_2$ sorption isotherms of **UoB-116(Dy$_1$La$_0$)** recorded at 273, 283, and 298 K over the pressure range of 0–1 bar. Filled symbol: adsorption; open symbol: desorption.

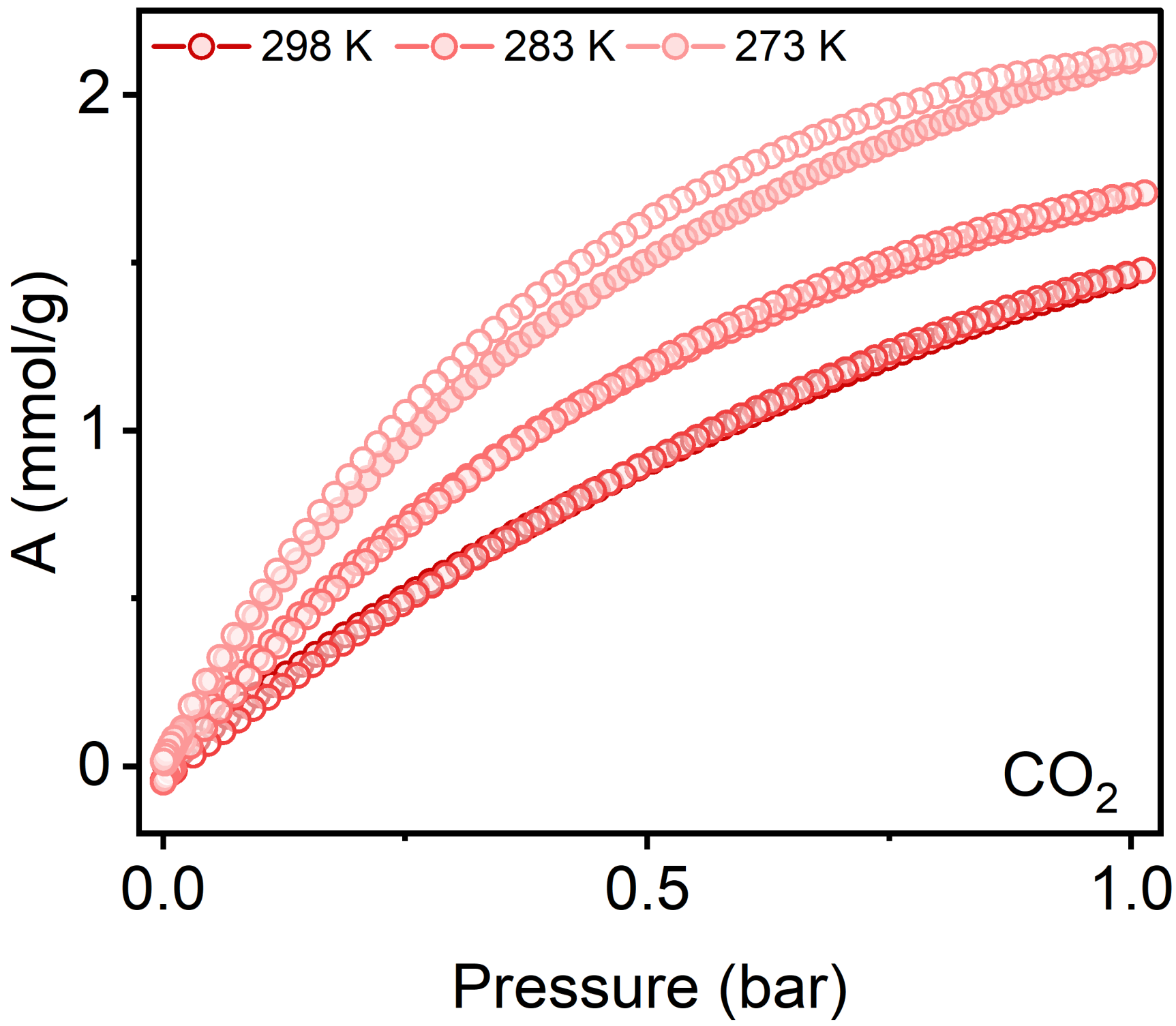


**Figure S33:** $CO_2$ sorption isotherms of **UoB-116(Dy$_{0.82}$La$_{0.18}$)** recorded at 273, 283, and 298 K over the pressure range of 0–1 bar. Filled symbol: adsorption; open symbol: desorption.

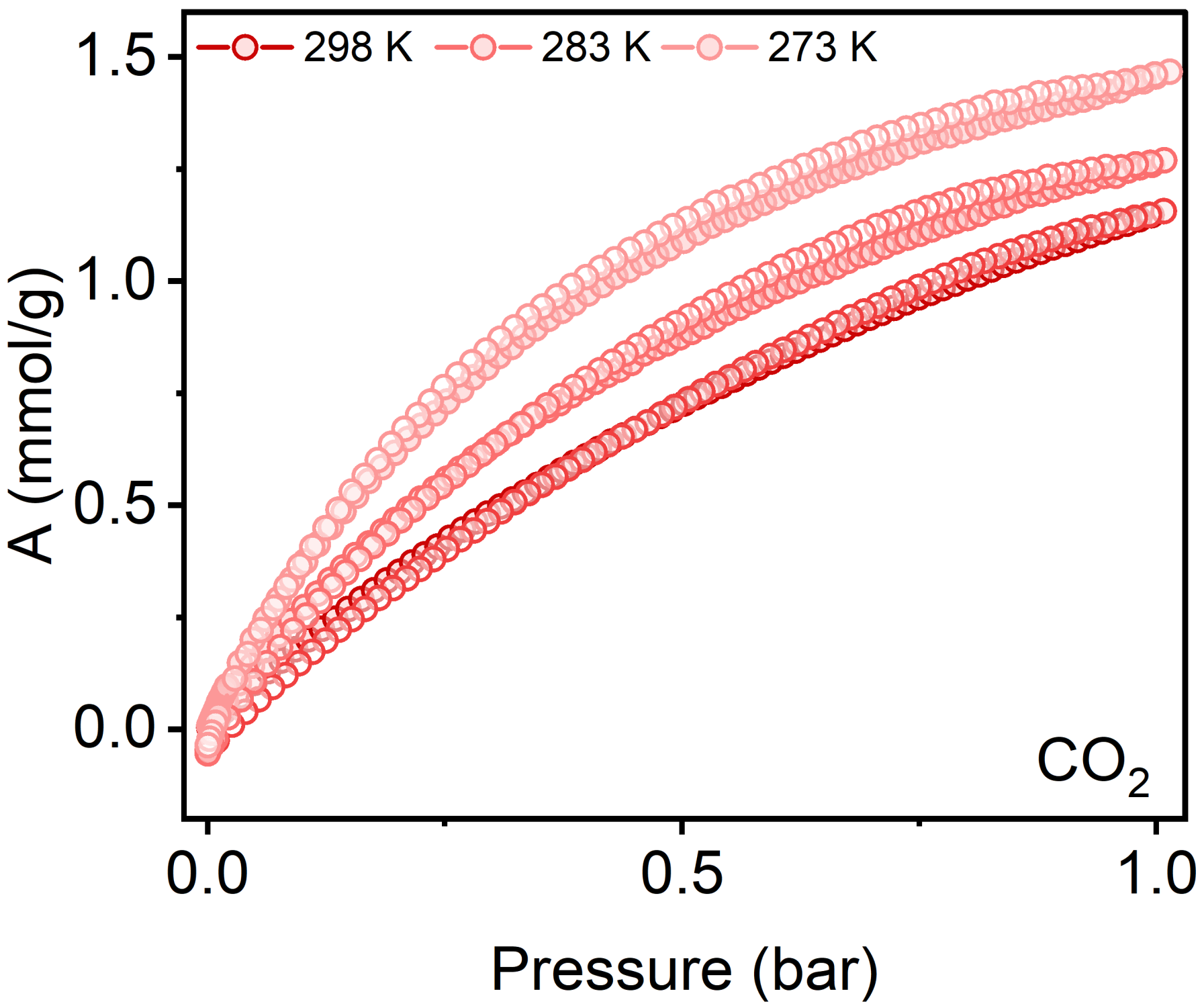


**Figure S34:** $CO_2$ sorption isotherms of **UoB-116(Dy$_{0.64}$La$_{0.36}$)** recorded at 273, 283, and 298 K over the pressure range of 0–1 bar. Filled symbol: adsorption; open symbol: desorption.

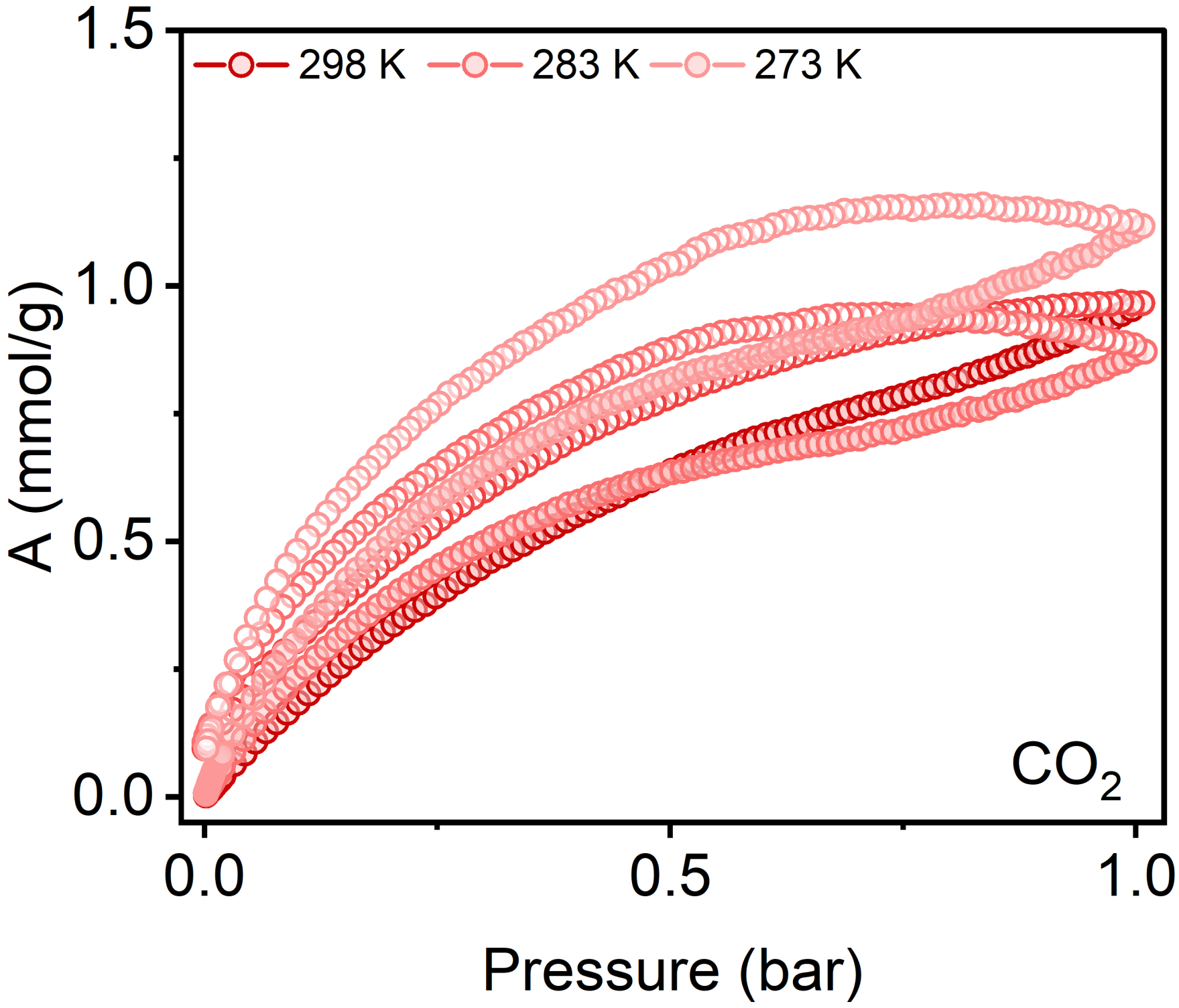


**Figure S35:** $CO_2$ sorption isotherms of **UoB-116(Dy$_{0.36}$La$_{0.64}$)** recorded at 273, 283, and 298 K over the pressure range of 0–1 bar. Filled symbol: adsorption; open symbol: desorption.

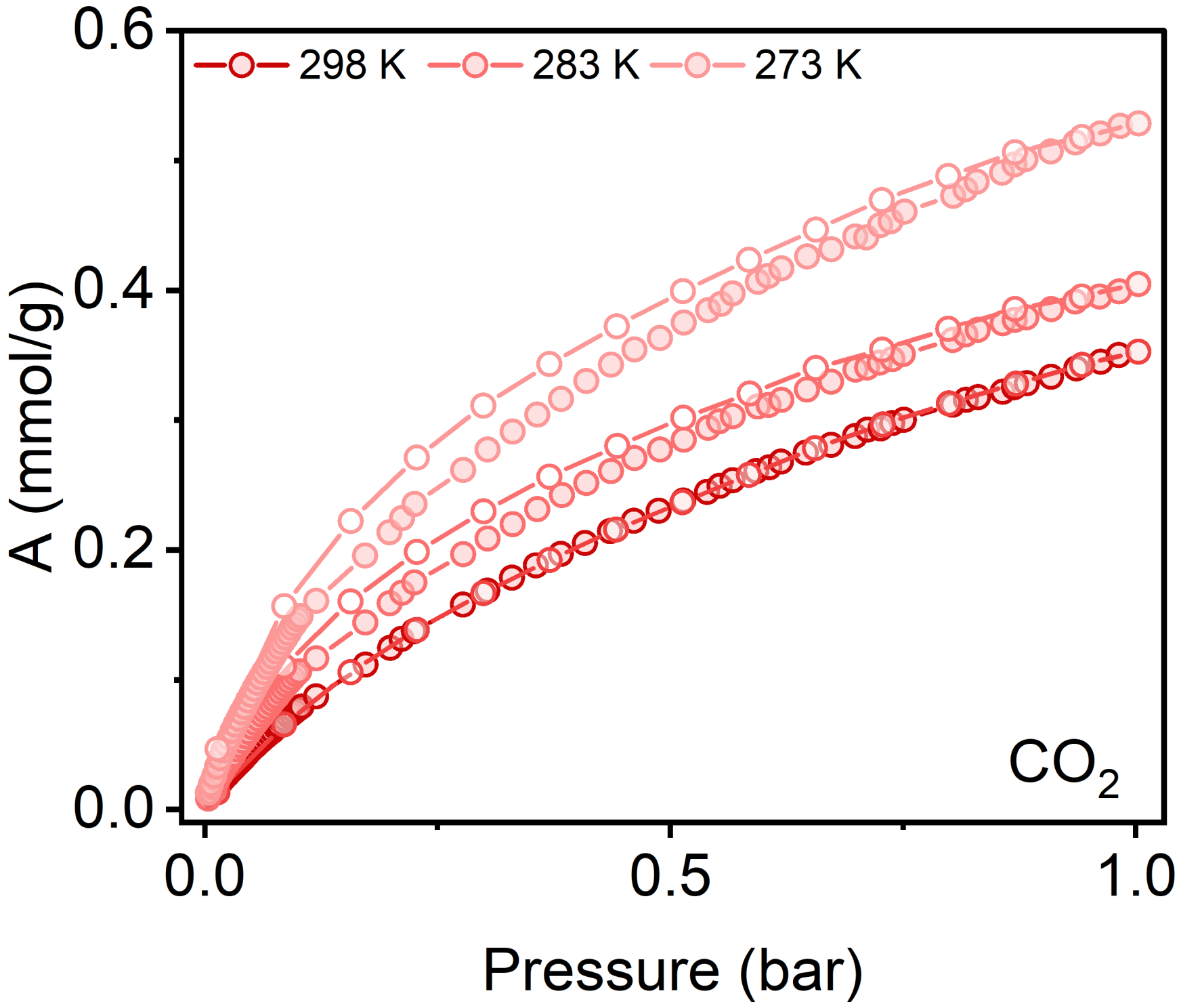


**Figure S36:** $CO_2$ sorption isotherms of **UoB-116($Dy_1La_0$)** recorded at 273, 283, and 298 K over the pressure range of 0–1 bar. Filled symbol: adsorption; open symbol: desorption.

**Adsorption energy calculations**

Isosteric heat of adsorption ($Q_{st}$) values was determined from isotherms recorded at 273 K, 283 K and 298 K for $CO_2$, using Micromeritics MicroActive® software. The adsorption data at the three temperatures were fitted to generate adsorption isosteres, where the equilibrium pressure at a fixed adsorbed amount was extracted for each temperature. The $Q_{st}$ values were then obtained using the Clausius–Clapeyron equation:

$$\ln(P) = -\frac{Q_{st}}{RT} + C$$

where P is the equilibrium pressure, R is the gas constant, T is the absolute temperature, and C is a constant.

For each fixed loading, ln(P) was plotted against 1/T . The slope of the linear fit is equal to:

$$m = -\frac{Q_{st}}{R}$$

Therefore, $Q_{st}$ was calculated as:

$$Q_{st} = -mR$$

where m is the slope obtained from the isostere fit. The resulting $Q_{st}$ values were plotted as a function of $CO_2$ loading.

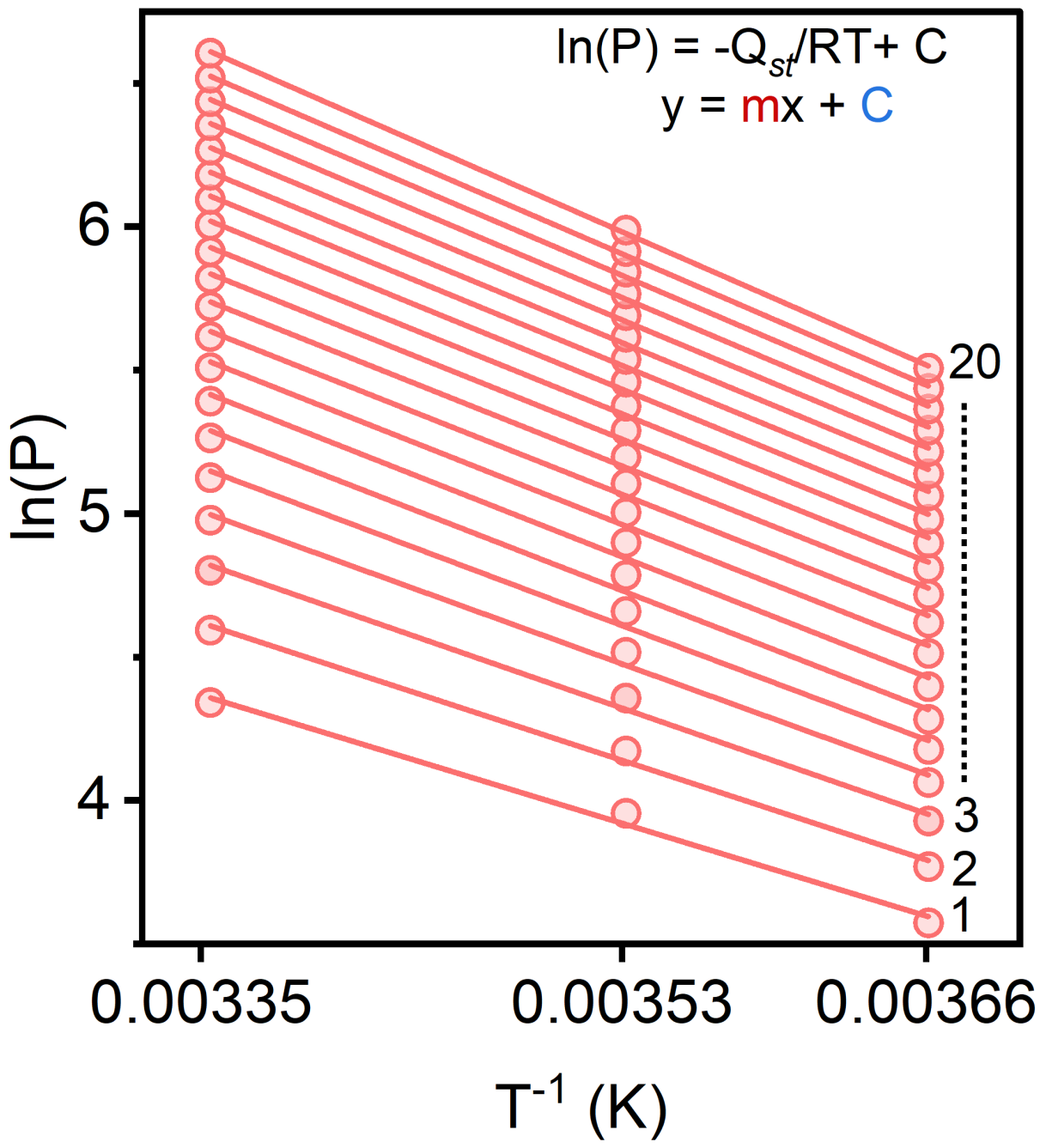


| Isosteres Linear Fit | | | |
|---|---|---|---|
| | m | C | Adj R square |
| 1 | -2483.9318 | 12.68912 | 0.98476 |
| 2 | -2665.5136 | 13.54935 | 0.98802 |
| 3 | -2827.5405 | 14.30339 | 0.98744 |
| 4 | -2955.6437 | 14.90964 | 0.98514 |
| 5 | -3061.0958 | 15.41562 | 0.98027 |
| 6 | -3166.3141 | 15.909 | 0.97835 |
| 7 | -3214.2261 | 16.19565 | 0.98155 |
| 8 | -3221.1313 | 16.33257 | 0.9862 |
| 9 | -3228.5273 | 16.46394 | 0.98907 |
| 10 | -3250.1626 | 16.63917 | 0.99134 |
| 11 | -3274.0685 | 16.81699 | 0.9927 |
| 12 | -3296.2787 | 16.98373 | 0.99377 |
| 13 | -3329.2223 | 17.18594 | 0.99488 |
| 14 | -3352.9443 | 17.35254 | 0.99595 |
| 15 | -3376.4409 | 17.5148 | 0.99673 |
| 16 | -3411.7215 | 17.7187 | 0.99755 |
| 17 | -3446.3127 | 17.91874 | 0.99826 |
| 18 | -3481.9348 | 18.12171 | 0.99842 |
| 19 | -3517.6453 | 18.32276 | 0.99892 |
| 20 | -3570.074 | 18.58468 | 0.99925 |

**Figure S37:** Clausius–Clapeyron plots and corresponding linear fitting parameters derived from the $CO_2$ adsorption isotherms measured at 273, 283, and 298 K for **UoB-116(Dy$_1$La$_0$)**, used to calculate the isosteric heat of adsorption ($Q_{st}$).

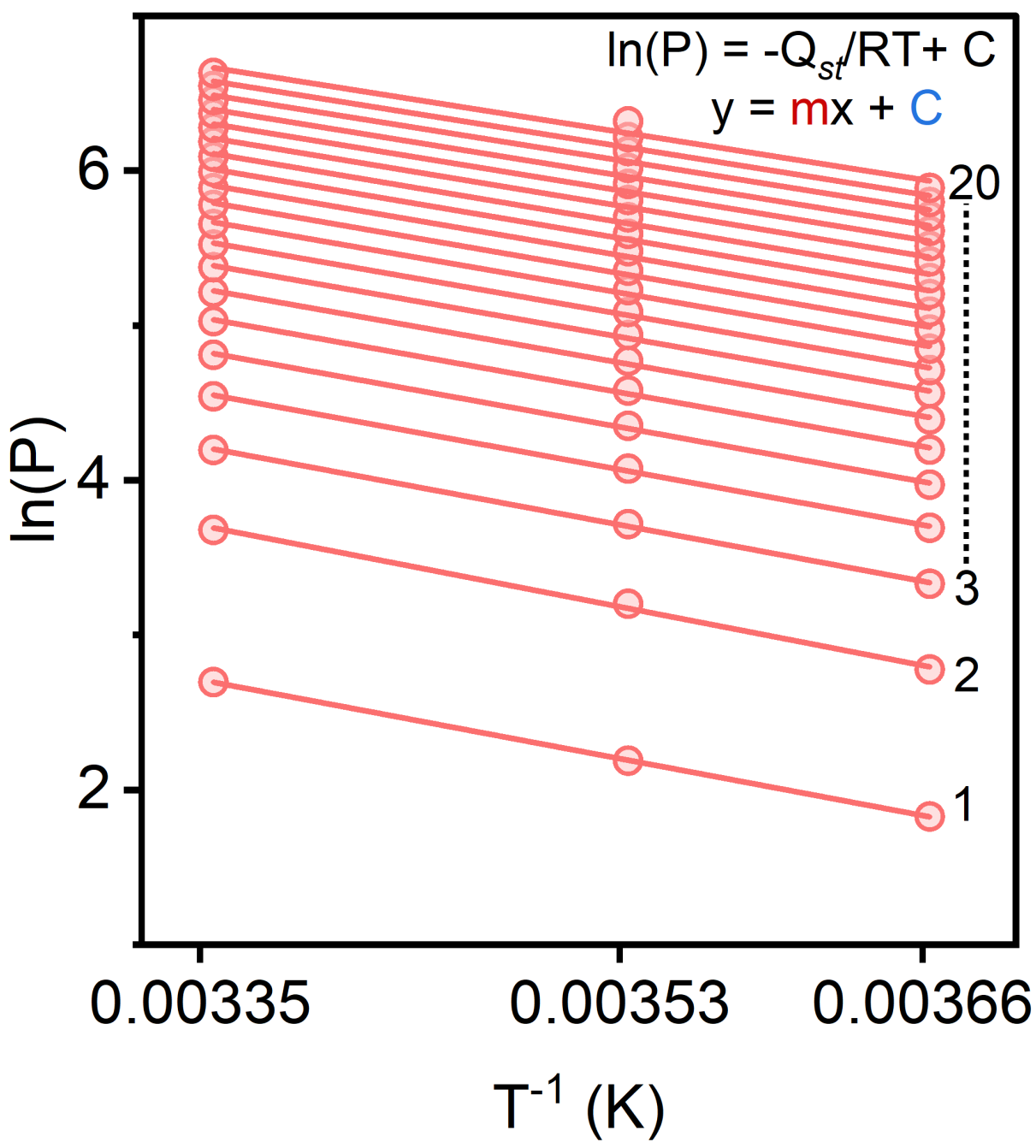


| Isosteres Fit | | | |
|---|---|---|---|
| | m | C | Adj R square |
| 1 | -2827.68 | 12.1843 | 0.99985 |
| 2 | -2919.24 | 13.4884 | 0.99343 |
| 3 | -2806.94 | 13.6211 | 0.99835 |
| 4 | -2754.19 | 13.7917 | 0.9979 |
| 5 | -2720.26 | 13.9469 | 0.99742 |
| 6 | -2691.29 | 14.0682 | 0.99603 |
| 7 | -2662.46 | 14.1585 | 0.99618 |
| 8 | -2635.20 | 14.2290 | 0.99567 |
| 9 | -2624.36 | 14.3390 | 0.99522 |
| 10 | -2608.90 | 14.4211 | 0.99375 |
| 11 | -2606.43 | 14.5372 | 0.99309 |
| 12 | -2579.71 | 14.5595 | 0.98775 |
| 13 | -2550.05 | 14.5655 | 0.98664 |
| 14 | -2531.29 | 14.6029 | 0.98412 |
| 15 | -2497.00 | 14.5869 | 0.97766 |
| 16 | -2460.61 | 14.5575 | 0.97211 |
| 17 | -2440.54 | 14.5838 | 0.96447 |
| 18 | -2409.28 | 14.5710 | 0.94912 |
| 19 | -2403.43 | 14.6417 | 0.95009 |
| 20 | -2376.54 | 14.6391 | 0.93411 |

**Figure S38:** Clausius–Clapeyron plots and corresponding linear fitting parameters derived from the $CO_2$ adsorption isotherms measured at 273, 283, and 298 K for **UoB-116(Dy$_{0.82}$La$_{0.18}$)**, used to calculate the isosteric heat of adsorption ($Q_{st}$).

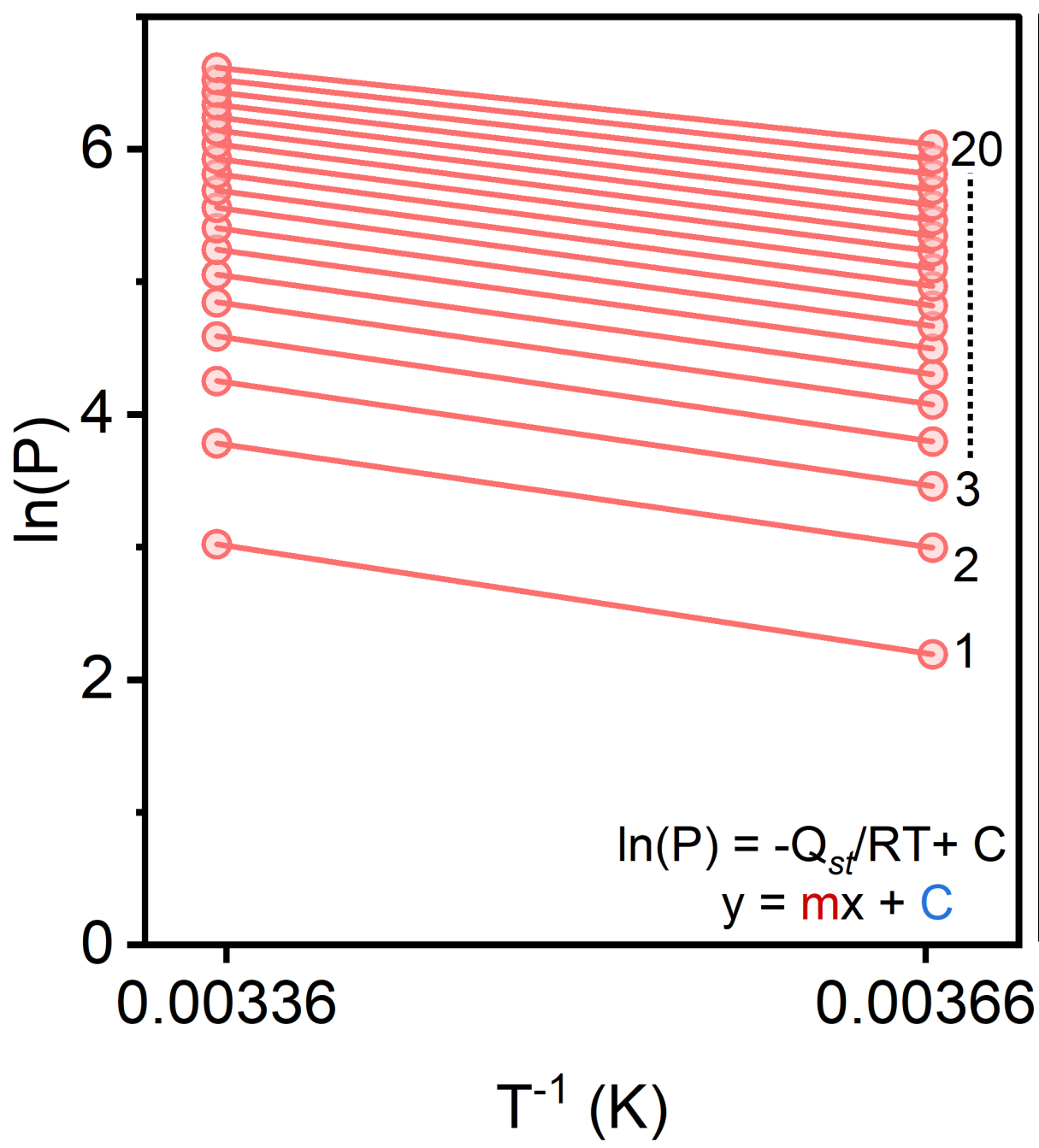


| Isosteres Fit | | | |
|---|---|---|---|
| | m | C | R-Square (COD) |
| 1 | -2702.8 | 12.09 | 1 |
| 2 | -2559.3 | 12.37 | 1 |
| 3 | -2581.0 | 12.91 | 1 |
| 4 | -2584.3 | 13.26 | 1 |
| 5 | -2512.4 | 13.27 | 1 |
| 6 | -2449.0 | 13.27 | 1 |
| 7 | -2431.3 | 13.40 | 1 |
| 8 | -2404.6 | 13.47 | 1 |
| 9 | -2404.5 | 13.62 | 1 |
| 10 | -2349.8 | 13.57 | 1 |
| 11 | -2314.4 | 13.57 | 1 |
| 12 | -2265.6 | 13.53 | 1 |
| 13 | -2252.0 | 13.59 | 1 |
| 14 | -2201.2 | 13.52 | 1 |
| 15 | -2156.5 | 13.47 | 1 |
| 16 | -2084.9 | 13.33 | 1 |
| 17 | -2012.4 | 13.18 | 1 |
| 18 | -1953.7 | 13.08 | 1 |
| 19 | -1886.6 | 12.94 | 1 |
| 20 | -- ± -- | -- ± -- | |

**Figure S39:** Clausius–Clapeyron plots and corresponding linear fitting parameters derived from the $CO_2$ adsorption isotherms measured at 273 and 298 K for **UoB-116($Dy_{0.64}La_{0.36}$)**, used to calculate the isosteric heat of adsorption ($Q_{st}$).

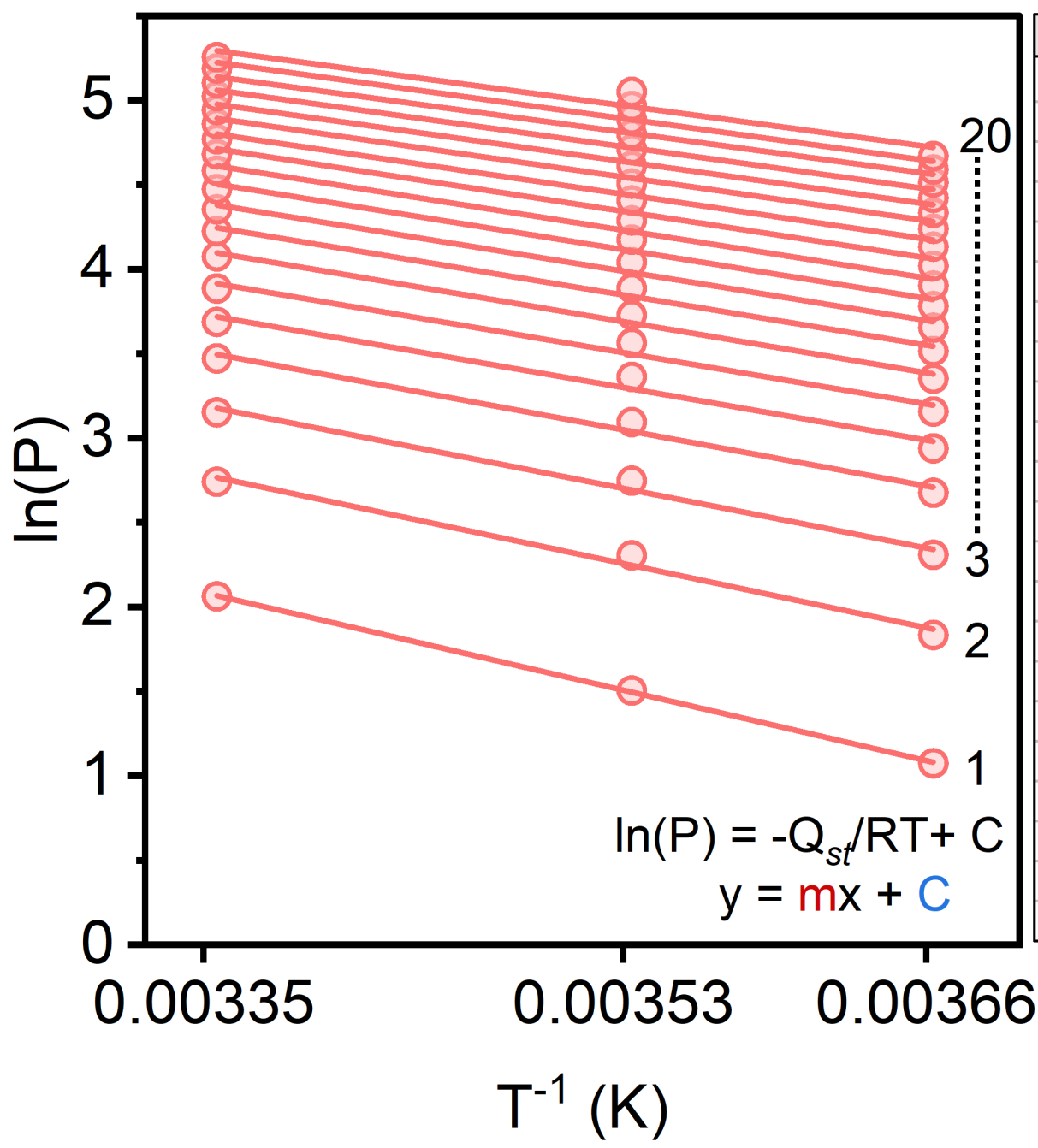


| Isosteres Fit | | | |
|---|---|---|---|
| | m | C | Adj R s |
| 1 | -3209.4071 ± 5 | 12.8364 | 0.9994 |
| 2 | -2922.53171 ± | 12.5733 | 0.9748 |
| 3 | -2718.82059 ± | 12.2995 | 0.974 |
| 4 | -2559.29296 ± | 12.0831 | 0.9710 |
| 5 | -2398.19244 ± | 11.7652 | 0.9461 |
| 6 | -2340.32998 ± | 11.7672 | 0.9508 |
| 7 | -2330.61475 ± | 11.9165 | 0.9745 |
| 8 | -2285.4603 ± 2 | 11.9147 | 0.9714 |
| 9 | -2245.69621 ± | 11.9154 | 0.9535 |
| 10 | -2220.68386 ± | 11.9546 | 0.9434 |
| 11 | -2179.19246 ± | 11.9233 | 0.9432 |
| 12 | -2118.01677 ± | 11.8189 | 0.9290 |
| 13 | -2038.00281 ± | 11.6380 | 0.9289 |
| 14 | -1984.06181 ± | 11.5492 | 0.9158 |
| 15 | -1936.25028 ± | 11.4728 | 0.8992 |
| 16 | -1916.48346 ± | 11.4903 | 0.8923 |
| 17 | -1878.10276 ± | 11.4408 | 0.8842 |
| 18 | -1902.41946 ± | 11.607 | 0.8891 |
| 19 | -1858.67215 ± | 11.5294 | 0.8658 |

**Figure S40:** Clausius–Clapeyron plots and corresponding linear fitting parameters derived from the $CO_2$ adsorption isotherms measured at 273, 283, and 298 K for **UoB-116($Dy_{0.36}La_{0.64}$)**, used to calculate the isosteric heat of adsorption ($Q_{st}$).

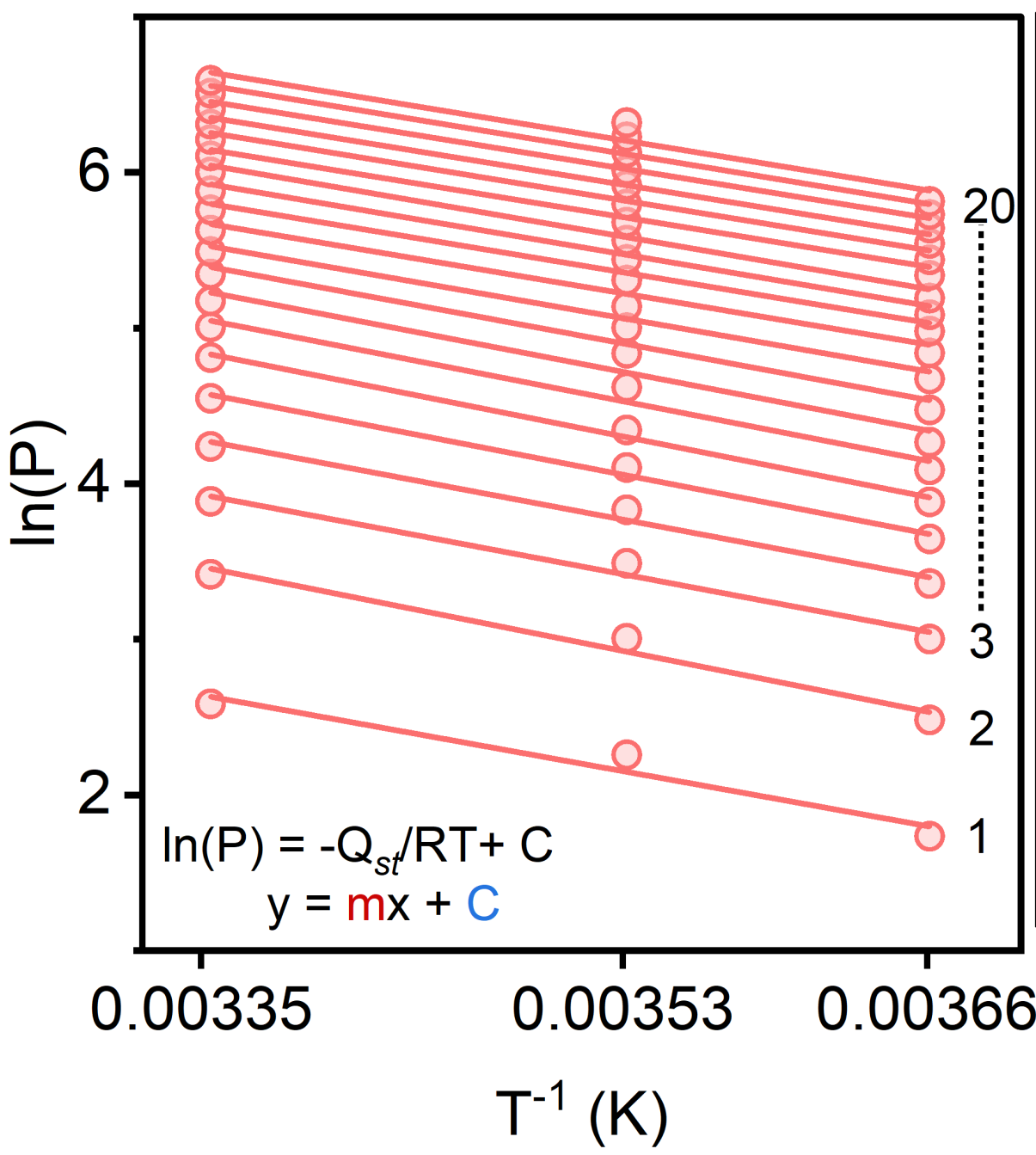


| Isosteres Fit | | | |
|---|---|---|---|
| | m | C | Adj R sq |
| 1 | -2713.99782 | 11.73415 | 0.90229 |
| 2 | -3003.45243 | 13.52817 | 0.94844 |
| 3 | -2842.06181 | 13.4513 ± | 0.95488 |
| 4 | -2838.01366 | 13.78794 | 0.96511 |
| 5 | -2916.54752 | 14.35359 | 0.98044 |
| 6 | -2994.21719 | 14.87356 | 0.98407 |
| 7 | -2939.68631 | 14.90708 | 0.93515 |
| 8 | -2895.91 ± 69 | 14.94136 | 0.88985 |
| 9 | -2789.63176 | 14.74887 | 0.91072 |
| 10 | -2624.35378 | 14.32737 | 0.94217 |
| 11 | -2525.91298 | 14.13974 | 0.92238 |
| 12 | -2495.02382 | 14.16556 | 0.92517 |
| 13 | -2548.86635 | 14.47211 | 0.9153 |
| 14 | -2592.83078 | 14.74075 | 0.91567 |
| 15 | -2451.20891 | 14.36556 | 0.91819 |
| 16 | -2462.49242 | 14.51318 | 0.89788 |
| 17 | -2446.43357 | 14.55717 | 0.90411 |
| 18 | -2438.50849 | 14.63315 | 0.88171 |
| 19 | -2478.5204 ± | 14.86961 | 0.87579 |
| 20 | -2471.12424 | 14.92983 | 0.86453 |

**Figure S41:** Clausius–Clapeyron plots and corresponding linear fitting parameters derived from the $CO_2$ adsorption isotherms measured at 273, 283, and 298 K for **UoB-116(Dy$_0$La$_1$)**, used to calculate the isosteric heat of adsorption ($Q_{st}$).

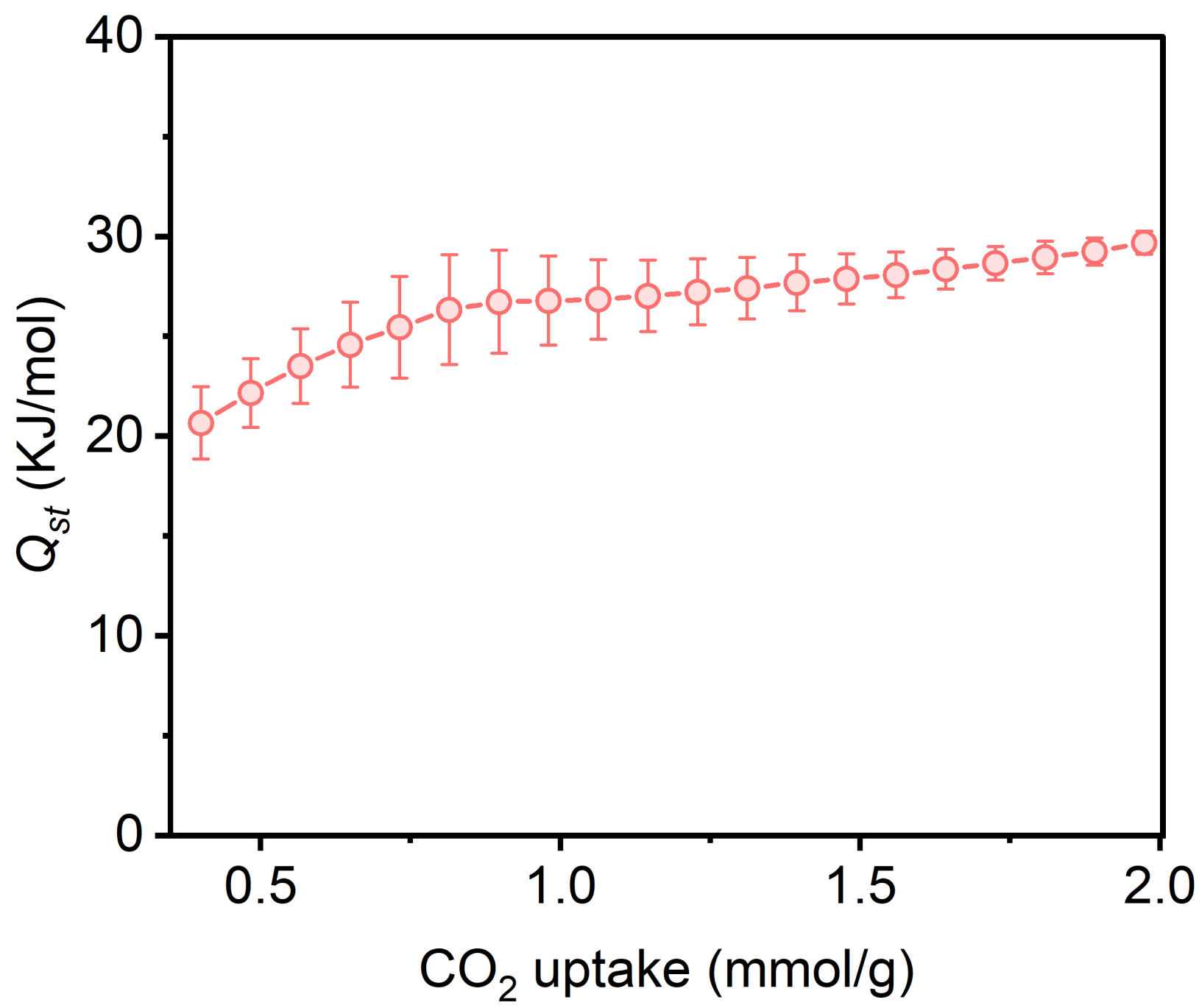


**Figure S42:** $Q_{st}(CO_2)$ as a function of $CO_2$ uptake for **UoB-116(Dy$_1$La$_0$)**, calculated from adsorption isotherms measured at 273, 283, and 298 K using the Clausius–Clapeyron equation. Error bars represent the uncertainty propagated from the linear fitting of the adsorption isosteres.

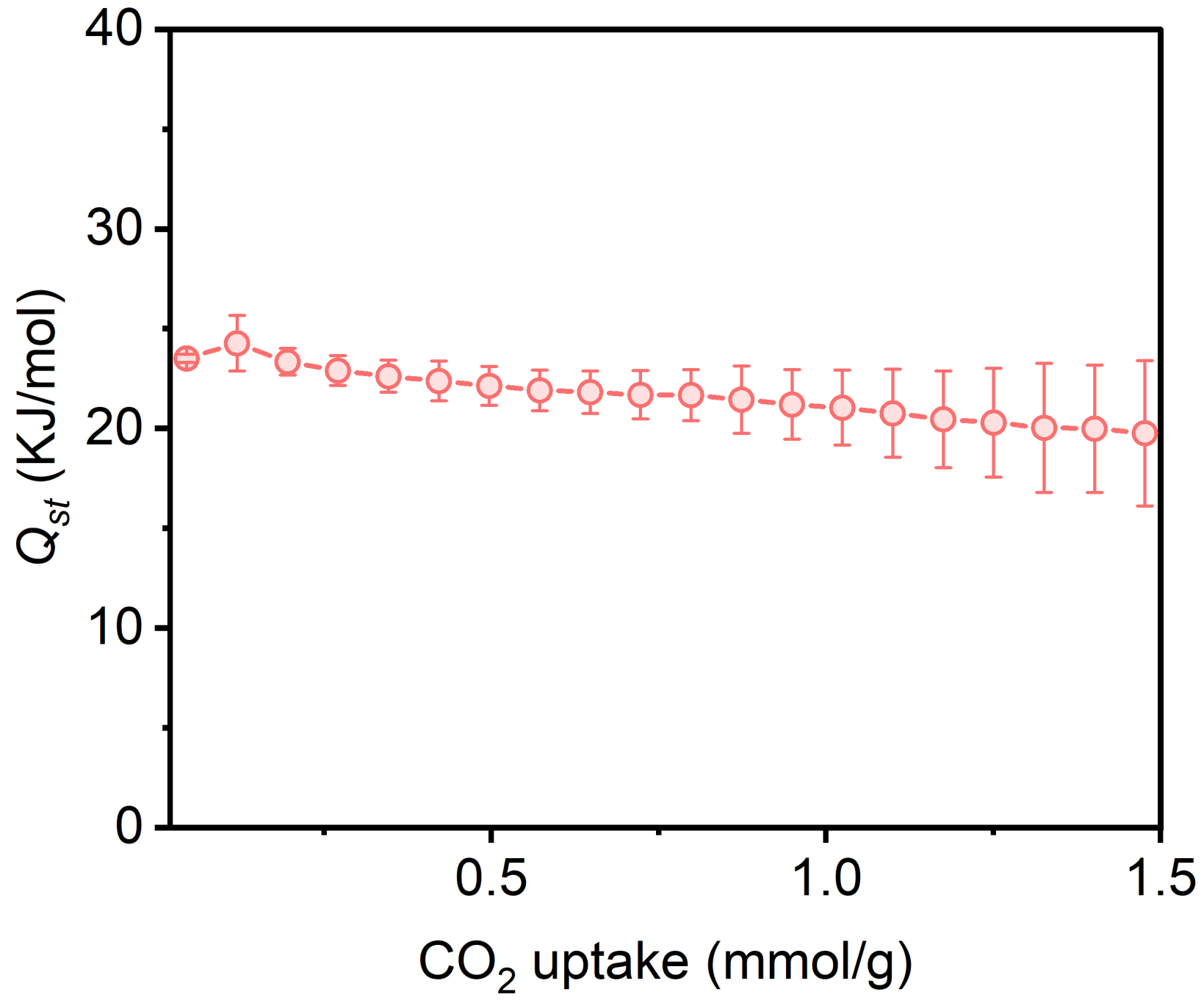


**Figure S43:** $Q_{st}(CO_2)$ as a function of $CO_2$ uptake for **UoB-116($Dy_{0.82}La_{0.18}$)**, calculated from adsorption isotherms measured at 273, 283, and 298 K using the Clausius–Clapeyron equation. Error bars represent the uncertainty propagated from the linear fitting of the adsorption isosteres.

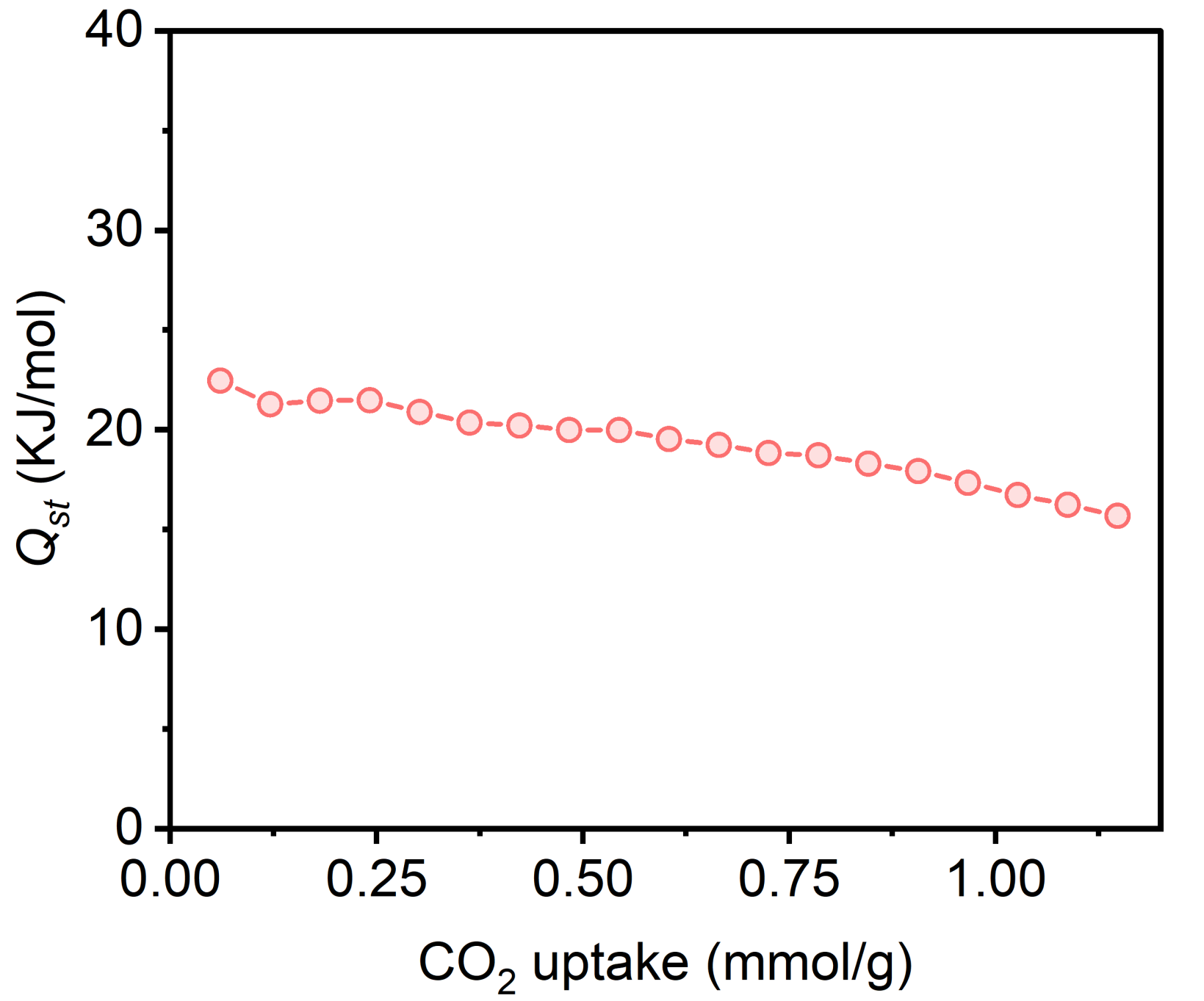


**Figure S44:** $Q_{st}(CO_2)$ as a function of $CO_2$ uptake for **UoB-116($Dy_{0.64}La_{0.36}$)**, calculated from adsorption isotherms measured at 273, and 298 K using the Clausius–Clapeyron equation.

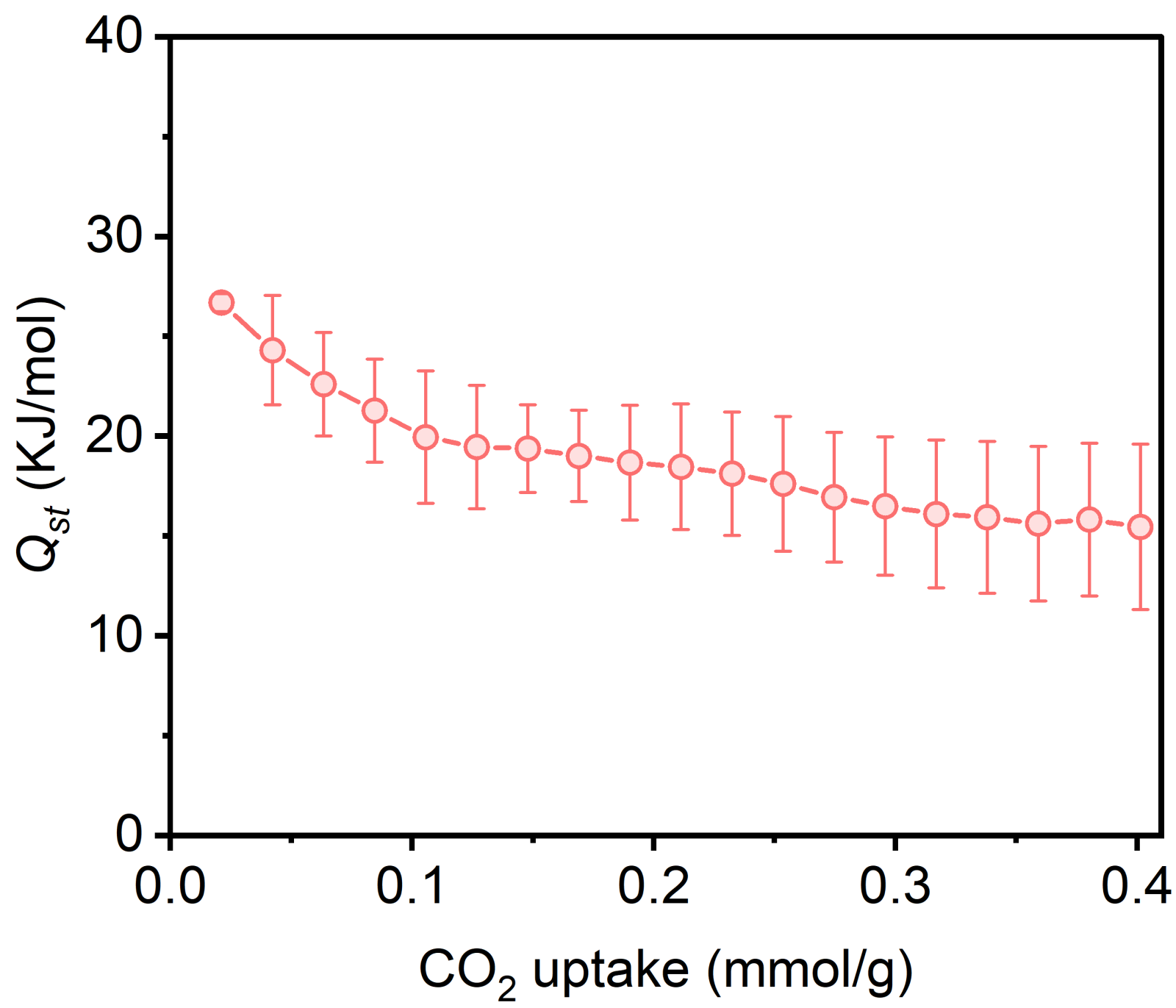


**Figure S45:** $Q_{st}(CO_2)$ as a function of $CO_2$ uptake for **UoB-116($Dy_{0.36}La_{0.64}$)**, calculated from adsorption isotherms measured at 273, 283, and 298 K using the Clausius–Clapeyron equation. Error bars represent the uncertainty propagated from the linear fitting of the adsorption isosteres.

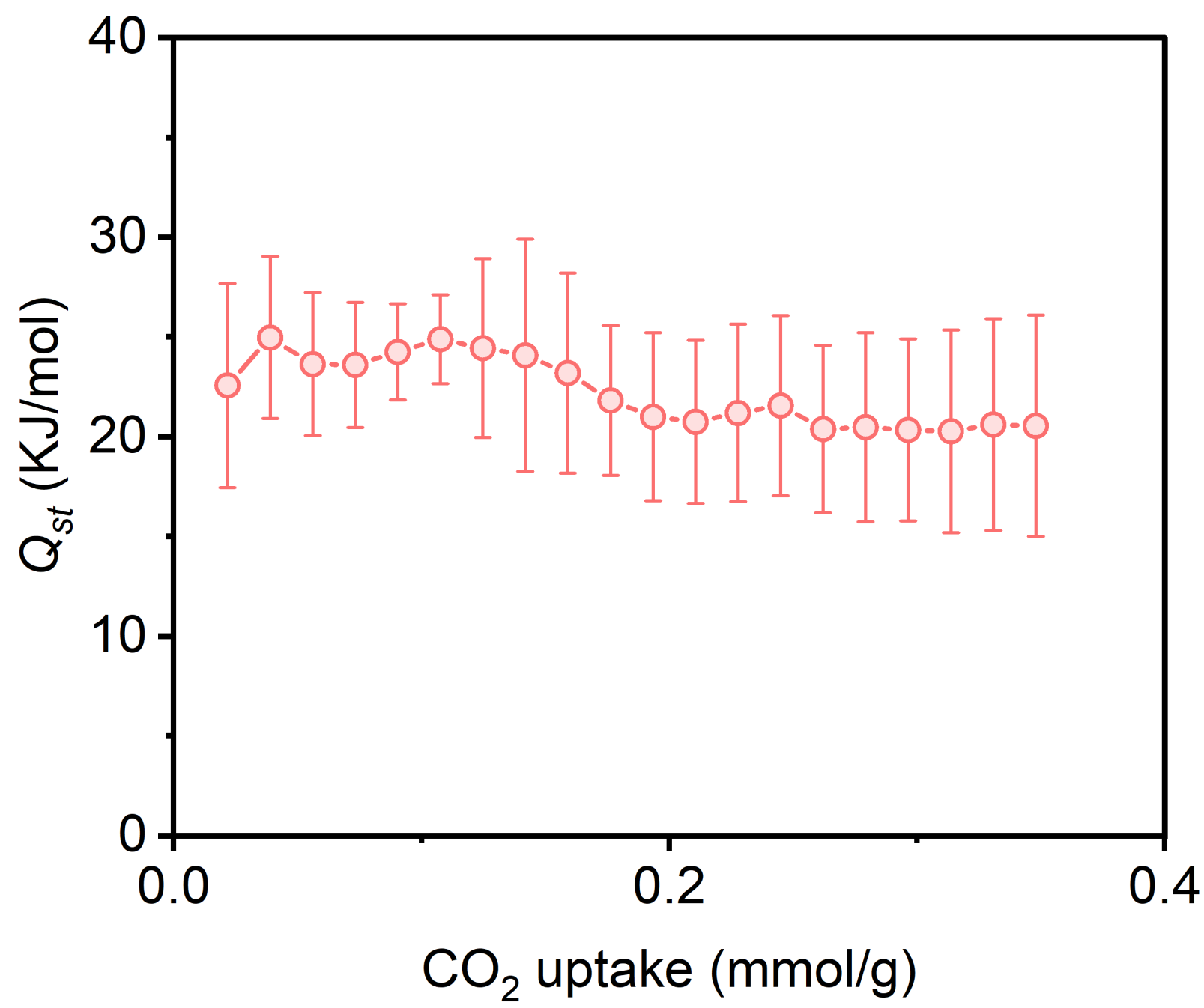


**Figure S46:** $Q_{st}(CO_2)$ as a function of $CO_2$ uptake for **UoB-116($Dy_0La_1$)**, calculated from adsorption isotherms measured at 273, 283, and 298 K using the Clausius–Clapeyron equation. Error bars represent the uncertainty propagated from the linear fitting of the adsorption isosteres.

**UV-visible Spectroscopy and Photochromism Studies**

Photochromism studies were conducted on MOF crystals which were first vacuum filtered and washed with 10mL acetone to remove surface DMF. The samples were irradiated for various periods of time using a 365 nm UV lamp before the UV-Vis spectra were collected between 250 and 900 nm using a Shimadzu UV-3600i spectrometer fitted with an integrating sphere for diffuse reflectance measurements. All measurements were referenced to a $BaSO_4$ standard.

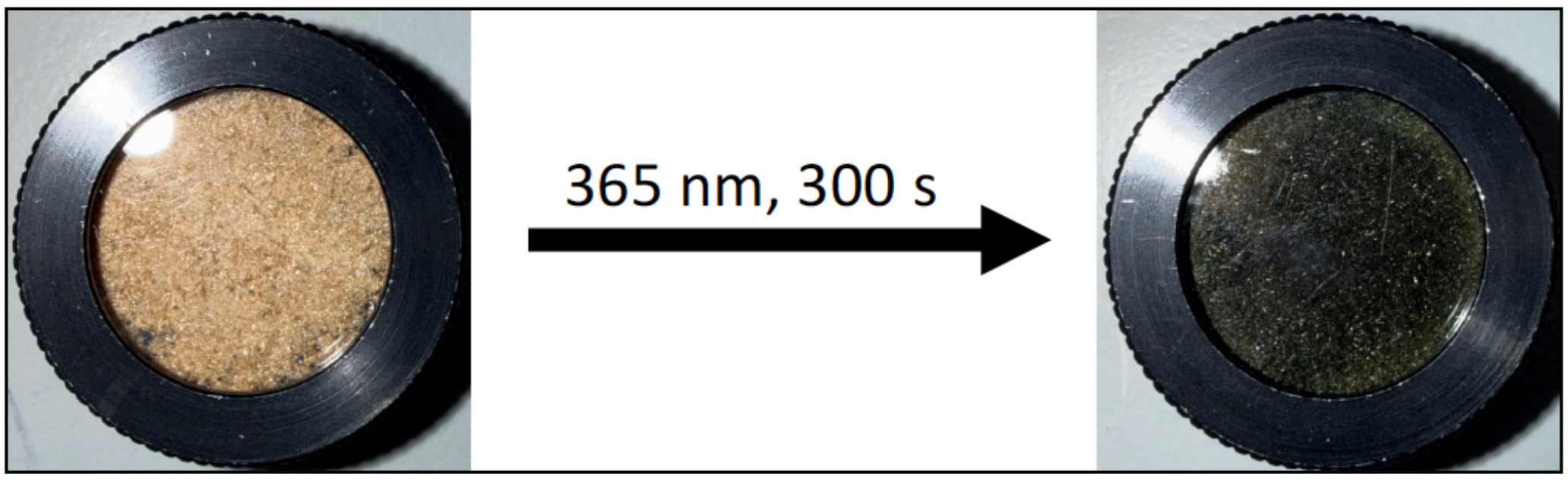


**Figure S47:** Photochromic response of **UoB-116($Dy_{0.64}La_{0.36}$)** following exposure with 365nm light for 300 s. Note the colour change of the sample which is replicated for all of the **UoB-116($Dy_{1-x}La_x$)** samples.

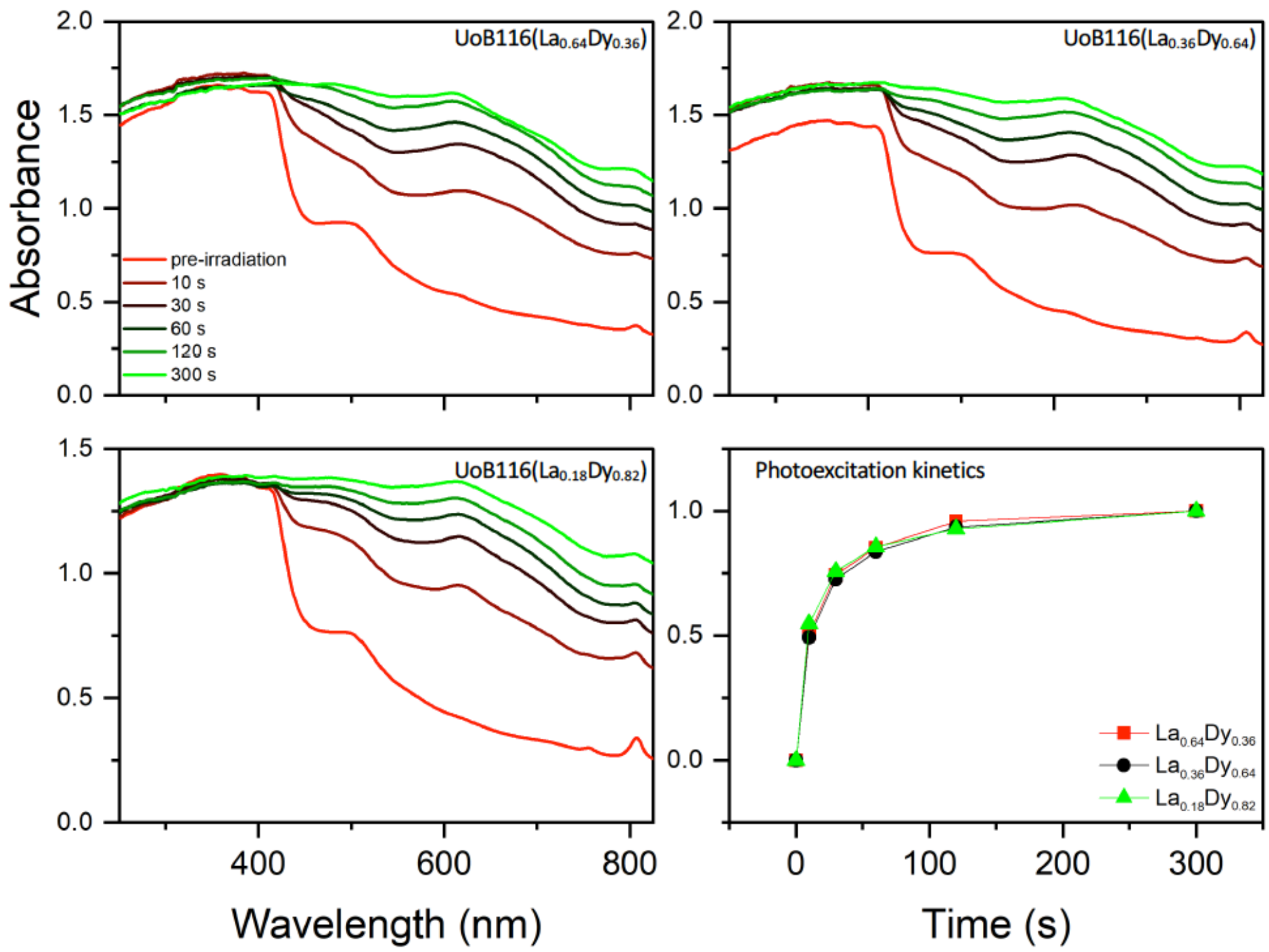


**Figure S48:** Solid-state UV-visible spectra of **UoB-116($Dy_{0.36}La_{0.64}$)**, **UoB-116($Dy_{0.64}La_{0.36}$)**, **UoB-116($Dy_{0.82}La_{0.18}$)** following exposure with 365nm light for 300 s. Photoexcitation kinetics are shown in the bottom right.

**Magnetic Measurements**

*Methods*: Magnetic susceptibility measurements were performed using a Quantum Design MPMS3 SQUID magnetometer. Each polycrystalline sample (10 – 17 mg) was ground to a powder using an agate mortar, loaded into a gelatin capsule, and mounted in a standard MPMS3 straw sample holder. DC magnetic susceptibility data were collected under both zero-field-cooled (ZFC) and field-cooled (FC) conditions upon warming from 2 to 300 K in an applied magnetic field of 0.01 T.

The molar magnetic susceptibility, $\chi_m$, was normalised per mole of total lanthanide (Ln = La + Dy). Curie–Weiss analysis was performed over the temperature range 25 – 300 K by fitting the susceptibility to $\chi_m = C/(T - \theta)$, where $C$ is the Curie constant and $\theta$ is the Weiss temperature. Experimental effective magnetic moments were calculated from the fitted Curie constants using $\mu_{eff} = 2.828\sqrt{C}$, with $C$ expressed in emu K Ln-mol$^{-1}$ Oe$^{-1}$, to give $\mu_{eff}$ in $\mu_B$ per mole of Ln.

The precise solvent content of the MOF samples could not be determined owing to variation in the amount of solvent retained within the pores of the structures. To account for the resulting uncertainty in formula mass, the molar susceptibility was calculated assuming lower and upper limits of one and three DMF molecules per formula unit, respectively. Curie–Weiss fits were performed using both limits, providing corresponding ranges for $C$ and $\mu_{eff}$. No diamagnetic correction was applied because the uncertain solvent content prevents an accurate determination of the total diamagnetic contribution to the measured susceptibility.

**Table S2.** Summary of the magnetic properties obtained from Curie–Weiss fits to the temperature-dependent magnetic susceptibility of the Dy-containing MOFs. The Dy content was determined by EDX and is expressed as a percentage of the total lanthanide content. The calculated effective magnetic moments, $\mu_{calc}$, assume free-ion $Dy^{3+}$ ($\mu_{eff}$ =10.65 $\mu_B$) and diamagnetic $La^{3+}$, and are normalised per mole of total lanthanide (Ln). Uncertainties in $\mu_{calc}$ for the mixed La/Dy samples reflect the 3% uncertainty in the Dy content determined by EDX. Experimental effective magnetic moments, $\mu_{exp}$, were calculated from the fitted Curie constants, $C$, using $\mu_{eff} = 2.828\sqrt{C}$. Ranges in $C$ and $\mu_{exp}$ reflect the upper and lower limits of the estimated solvent content within the MOF structures. The Weiss temperatures, $\theta$, are obtained from the same Curie–Weiss fits.

| **% Dy (EDX)** | **$\mu_{calc}$ ($\mu_B$ / Ln-mol)** | ***C* (emu K / Ln-mol-Oe)** | **$\mu_{exp}$ ($\mu_B$ / Ln-mol)** | **$\theta$ (K)** |
|---|---|---|---|---|
| 100.0 | 10.65 | 9.76(4) | 8.83(2) | –2.6(3) |
| 82.3(3) | 9.66(14) | 8.88(3) – 9.51(3) | 8.43(1) – 8.72(1) | –4.1(3) |
| 63.9(3) | 8.51(13) | 6.80(2) – 7.29(3) | 7.37(1) – 7.64(2) | –4.3(3) |
| 35.9(3) | 6.38(10) | 3.65(1) – 3.91(2) | 5.40(1)–5.59(1) | –3.5(3) |

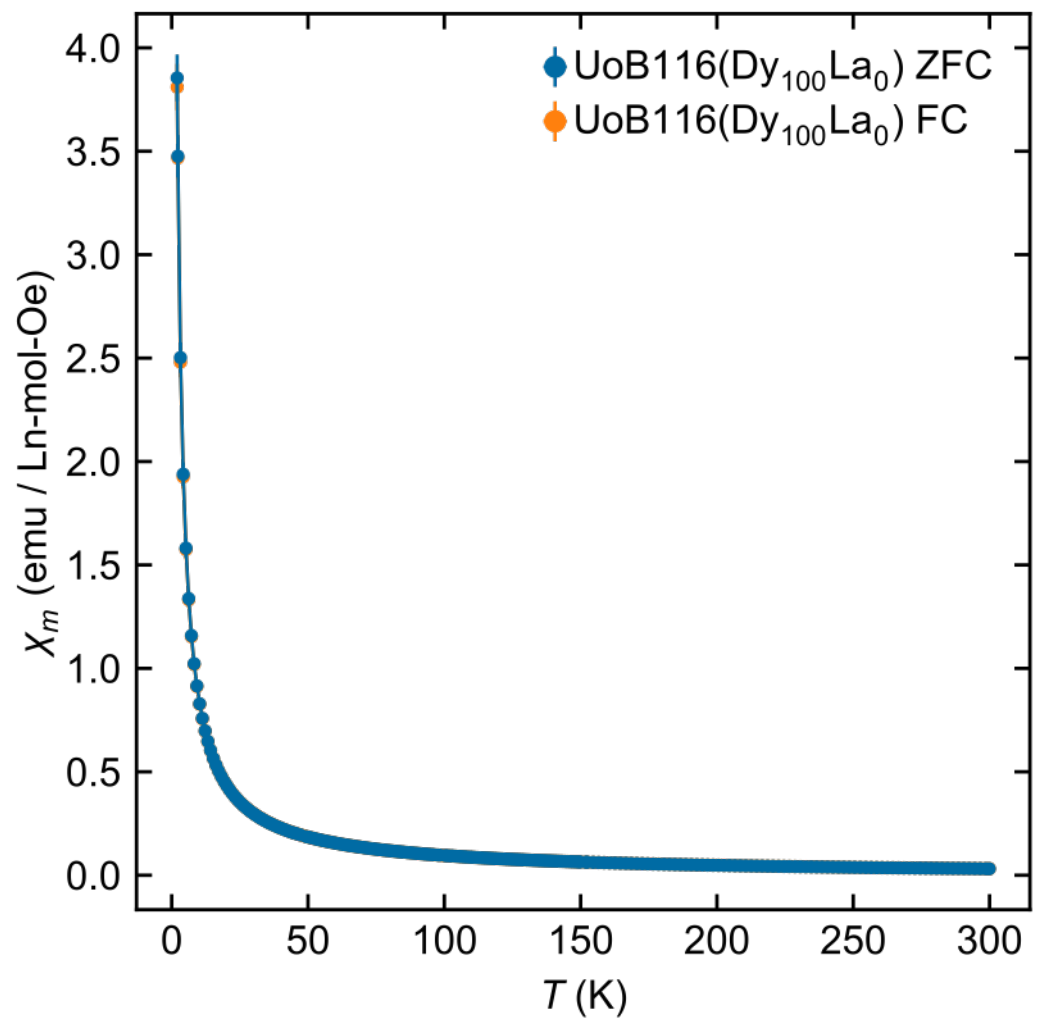


**Figure S49.** Temperature dependence of the magnetic susceptibility of **UoB-116($Dy_1La_0$)** measured under an applied magnetic field of 100 Oe. Magnetic susceptibility, $\chi_m$, of the **UoB-116($Dy_1La_0$)** sample measured under zero-field-cooled (ZFC) and field-cooled (FC) conditions.

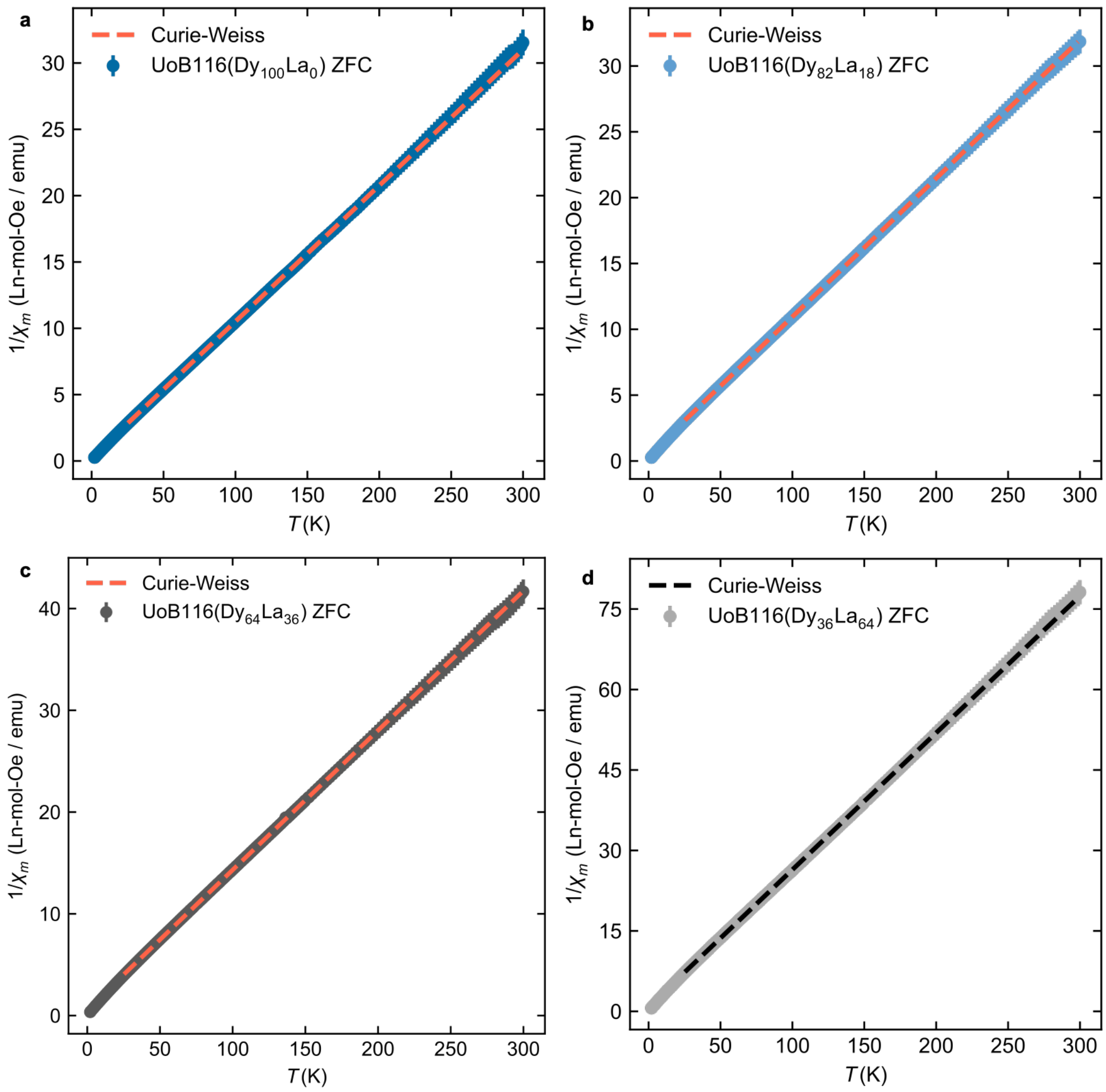


**Figure S50.** Curie–Weiss analysis of the temperature-dependent magnetic susceptibility of MOFs containing a) **UoB-116($Dy_1La_0$)**, b) **UoB-116($Dy_{0.82}La_{0.18}$)**, c) **UoB-116($Dy_{0.64}La_{0.36}$)**, and d)

**UoB-116($Dy_{0.36}La_{0.64}$)**, as determined by EDX. The inverse magnetic susceptibility, $1/\chi_m$, is normalised per mole of total lanthanide (Ln). Dashed lines show fits to the Curie–Weiss law, from which the Curie constants, effective magnetic moments, and Weiss temperatures reported in Table 1 were obtained.

**Cluster vs. Chains**

The number of possible combinations and sequences of different metals is greater in a chain than a corresponding cluster. This situation arises due to the absence of local symmetry in chains, in comparison to clusters, as illustrated in Figure S51. In the case shown 3 different metals are arranged into a chain or a cluster. In the case of the chain three different arrangements are possible, but in the cluster only one arrangement is possible (in terms of nearest neighbours). Thus, in the case of UoB-1116 the chain SBU leads to a greater number of possible combinations than in a comparable cluster SBU.

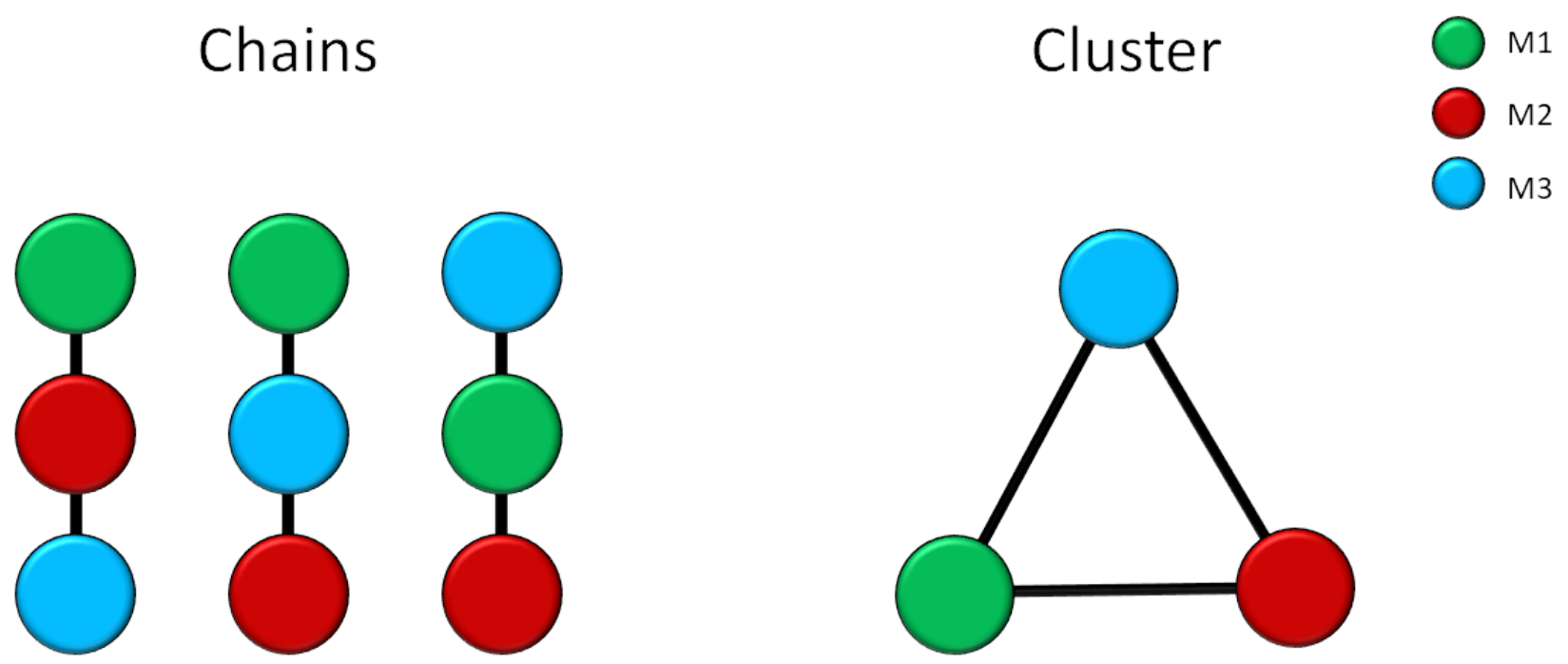


**Figure S51**. Possible combinations of different metals in a chain in comparison to a cluster for a system containing 3 different metals.

**References**

S1. O. V. Dolomanov, L. J. Bourhis, R.J. Gildea, J. A. K. Howard, H. Puschmann, *J. Appl. Cryst.* 2009, **42**, 339-341.

S2. G.M. Sheldrick, *Acta Cryst.*, 2015, **A71**, 3-8.

S3. G.M. Sheldrick, *Acta Cryst.*, 2015, **C71**, 3-8.
